# Supplementary material for: Pyridinium Salts of Dehydrated Lanthanide Polychlorides
Source: Molecules. 2022 Dec 29;28(1):283. doi: 10.3390/molecules28010283 (PMC9822437; doi:10.3390/molecules28010283)

molecules

Supporting Information

for:

Pyridinium Salts of Dehydrated Lanthanide Polychlorides

Roger E. Cramer, Esteban M. Baca, Timothy J. Boyle*

List of Figures.

Figure S1: Analytical FTIR data for **[$LaCl_6$]•py-H-py, 2(H-py) (La-6).**
Figure S2: Analytical FTIR data for **[$CeCl_6$]•py-H-py, 2(H-py) (Ce-6).**
Figure S3: Analytical FTIR data for **[$NdCl_6$]•py-H-py, 2(H-py) (Nd-6).**
Figure S4: Analytical FTIR data for **[$SmCl_6$]•py-H-py, 2(H-py) (Sm-6).**
Figure S5: Analytical FTIR data for **[$EuCl_6$]•py-H-py, 2(H-py) (Eu-6).**
Figure S6: Analytical FTIR data for **[$GdCl_6$]•py-H-py, 2(H-py) (Gd-6).**
Figure S7: Analytical FTIR data for **[$TbCl_5$(py)]•2(H-py) (Tb-5).**
Figure S8: Analytical FTIR data for **[$DyCl_5$(py)]•2(H-py) (Dy-5).**
Figure S9: Analytical FTIR data for **[$HoCl_5$(py)]•2(H-py) (Ho-5).**
Figure S10: Analytical FTIR data for **[$ErCl_5$(py)]•2(H-py) (Er-5).**
Figure S11: Analytical FTIR data for **[$TmCl_5$(py)]•2(H-py) (Tm-5).**
Figure S12: Analytical FTIR data for **[$YbCl_5$(py)]•2(H-py) (Yb-5).**
Figure S13: Analytical FTIR data for **[$LuCl_5$(py)]•2(H-py) (Lu-5).**
Figure S14: Analytical FTIR data for **[$PrCl_6$]•py-H-py, 2(H-py) (Pr-6).**
Figure S15: Theoretical PXRD data for **[$LaCl_6$]•py-H-py, 2(H-py) (La-6).**
Figure S15.1: Experimental PXRD data for **[$LaCl_6$]•py-H-py, 2(H-py) (La-6).**
Figure S16: Theoretical PXRD data for **[$CeCl_6$]•py-H-py, 2(H-py) (Ce-6).**
Figure S16.1: Experimental PXRD data for **[$CeCl_6$]•py-H-py, 2(H-py) (Ce-6).**
Figure S17: Theoretical PXRD data for **[$NdCl_6$]•py-H-py, 2(H-py) (Nd-6).**
Figure S17.1: Experimental PXRD data for **[$NdCl_6$]•py-H-py, 2(H-py) (Nd-6).**
Figure S18: Theoretical PXRD data for **[$SmCl_6$]•py-H-py, 2(H-py) (Sm-6).**
Figure S18.1: Experimental PXRD data for **[$SmCl_6$]•py-H-py, 2(H-py) (Sm-6).**
Figure S19: Theoretical PXRD data for **[$EuCl_6$]•py-H-py, 2(H-py) (Eu-6).**
Figure S19.1: Experimental PXRD data for **[$EuCl_6$]•py-H-py, 2(H-py) (Eu-6).**
Figure S20: Theoretical PXRD data for **[$GdCl_6$]•py-H-py, 2(H-py) (Gd-6).**
Figure S20.1: Experimental PXRD data for **[$GdCl_6$]•py-H-py, 2(H-py) (Gd-6).**
Figure S21: Theoretical PXRD data for **[$TbCl_5$(py)]•2(H-py) (Tb-5).**
Figure S21.1: Experimental PXRD data for **[$TbCl_5$(py)]•2(H-py) (Tb-5).**
Figure S22: Theoretical PXRD data for **[$DyCl_5$(py)]•2(H-py) (Dy-5).**
Figure S22.1: Experimental PXRD data for **[$DyCl_5$(py)]•2(H-py) (Dy-5).**
Figure S23: Theoretical PXRD data for **[$HoCl_5$(py)]•2(H-py) (Ho-5).**
Figure S23.1: Experimental PXRD data for **[$HoCl_5$(py)]•2(H-py) (Ho-5).**
Figure S24: Theoretical PXRD data for **[$ErCl_5$(py)]•2(H-py) (Er-5).**
Figure S24.1: Experimental PXRD data for **[$ErCl_5$(py)]•2(H-py) (Er-5).**
Figure S25: Theoretical PXRD data for **[$TmCl_5$(py)]•2(H-py) (Tm-5).**
Figure S25.1: Experimental PXRD data for **[$TmCl_5$(py)]•2(H-py) (Tm-5).**
Figure S26: Theoretical PXRD data for **[$YbCl_5$(py)]•2(H-py) (Yb-5).**
Figure S26.1: Experimental PXRD data for **[$YbCl_5$(py)]•2(H-py) (Yb-5).**
Figure S27: Theoretical PXRD data for **[$LuCl_5$(py)]•2(H-py) (Lu-5).**
Figure S27.1: Experimental PXRD data for **[$LuCl_5$(py)]•2(H-py) (Lu-5).**
Figure S28: Theoretical PXRD data for **[$PrCl_6$]•py-H-py, 2(H-py) (Pr-6).**
Figure S28.1: Experimental PXRD data for **[$PrCl_6$]•py-H-py, 2(H-py) (Pr-6).**

Figure S1: Analytical FTIR data for **[LaCl$_6$]•py-H-py, 2(H-py) (La-6)**

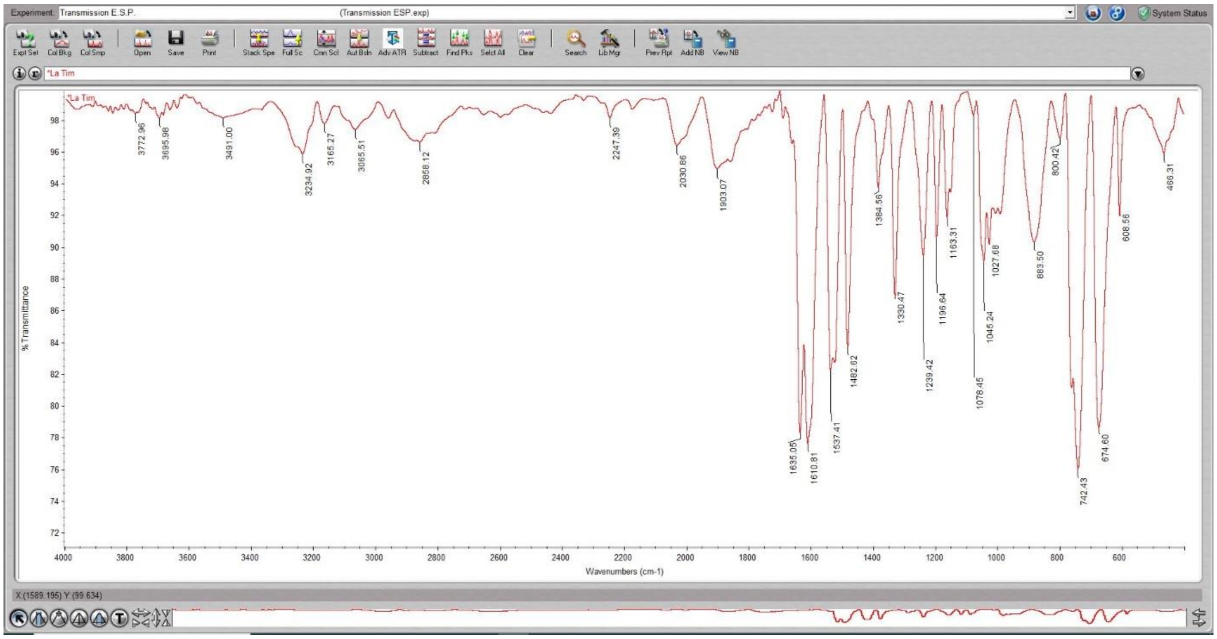

Figure S2: Analytical FTIR data for **[CeCl$_6$]•py-H-py, 2(H-py) (Ce-6)**

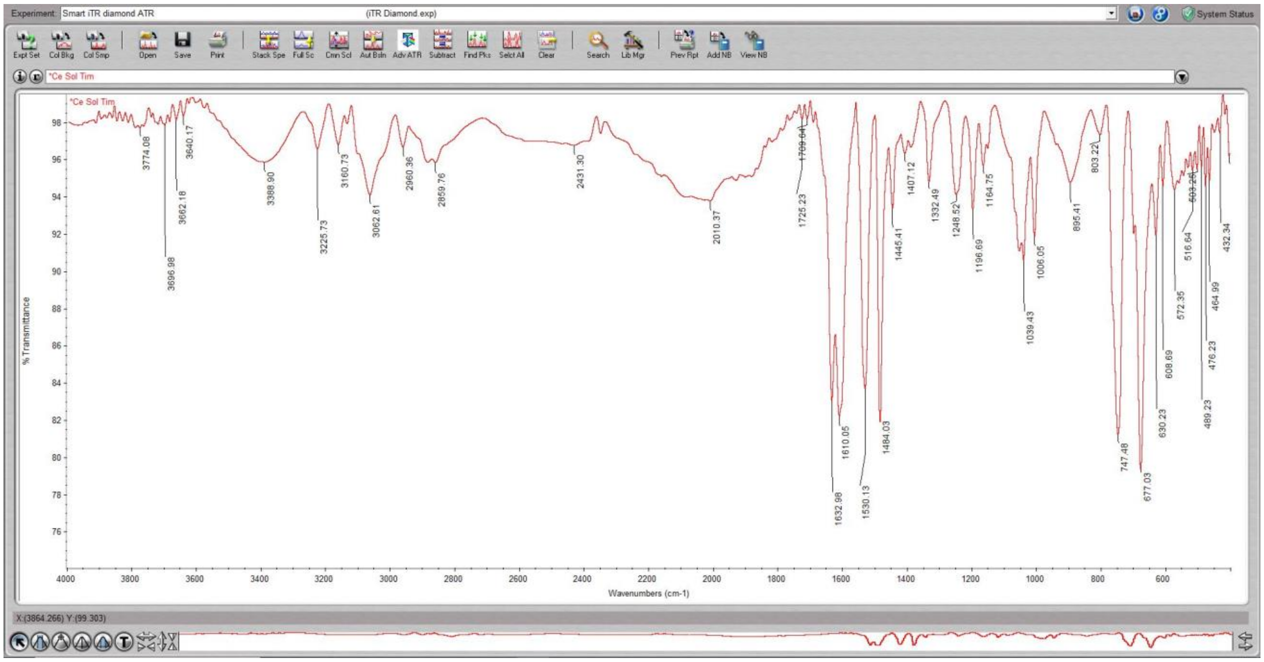

Figure S3: Analytical FTIR data for **[NdCl6]•py-H-py, 2(H-py) (Nd-6)**

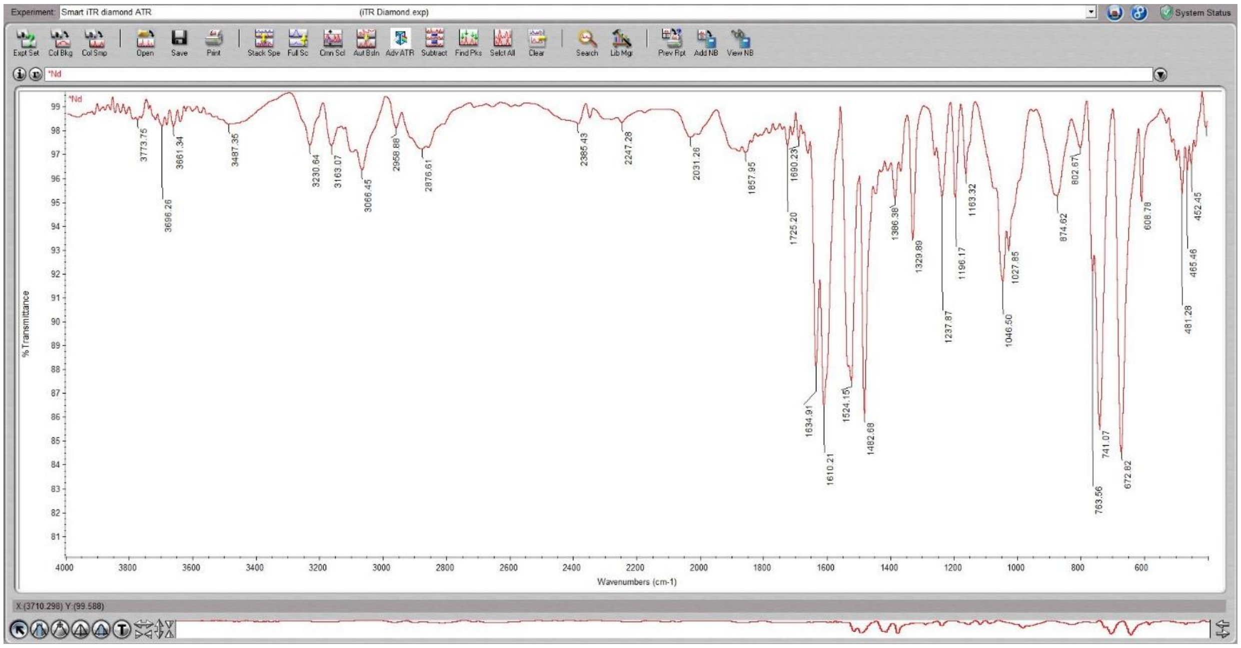

Figure S4: Analytical FTIR data for **[SmCl6]•py-H-py, 2(H-py) (Sm-6)**

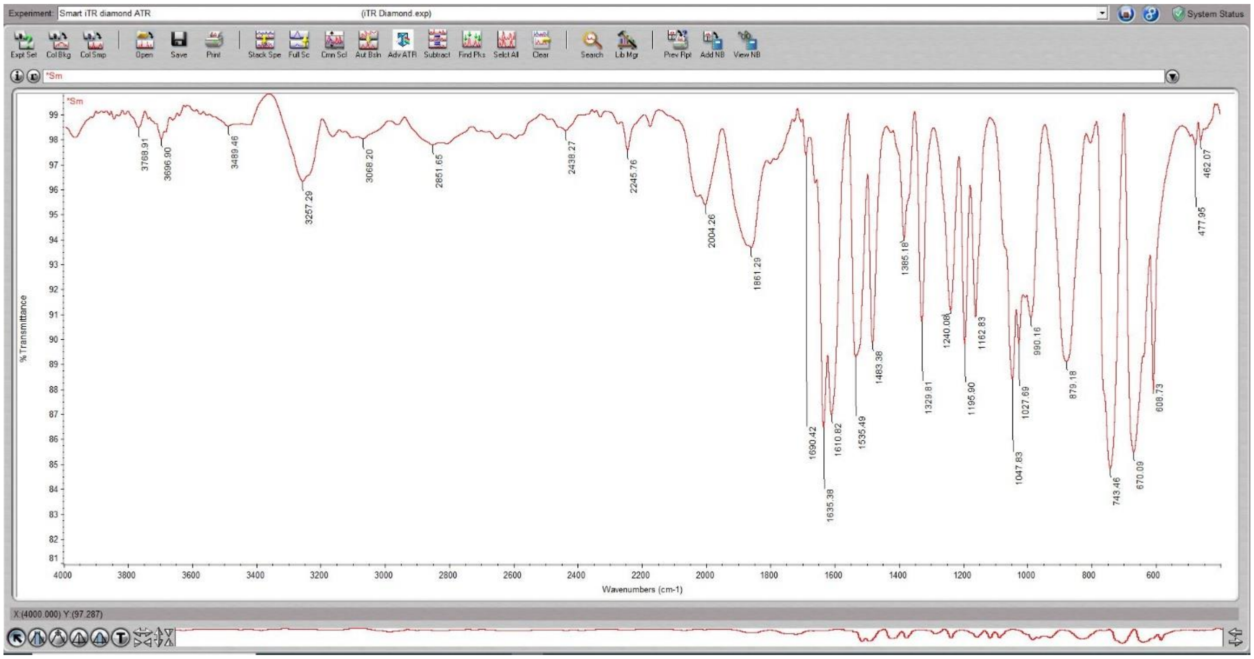

Figure S5: Analytical FTIR data for **[EuCl₆]•py-H-py, 2(H-py) (Eu-6)**

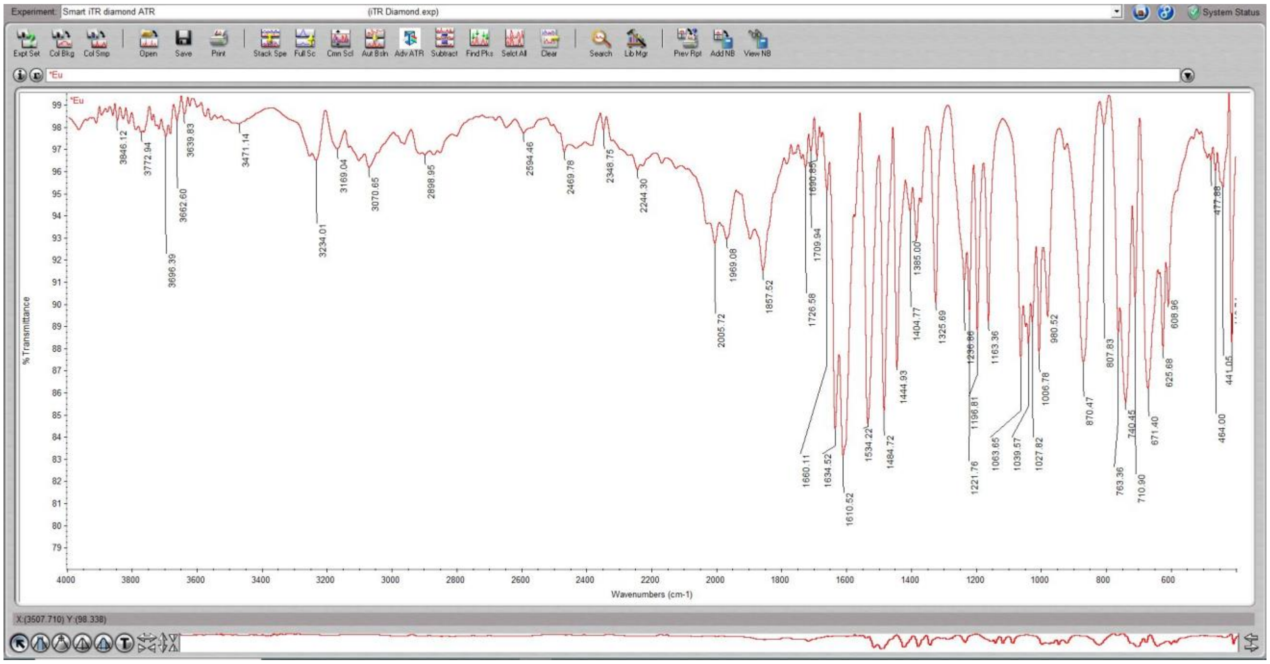

Figure S6: Analytical FTIR data for **[GdCl₆]•py-H-py, 2(H-py) (Gd-6)**

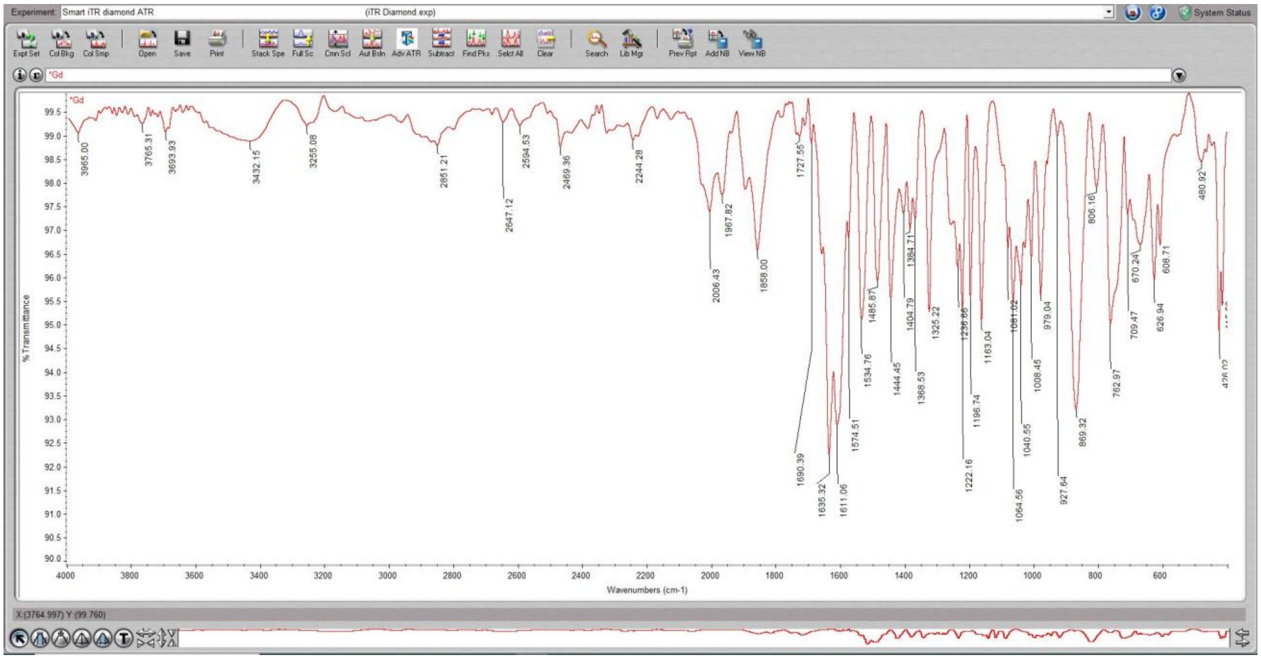

Figure S7: Analytical FTIR data for **[TbCl$_5$(py)]•2(H-py) (Tb-5)**

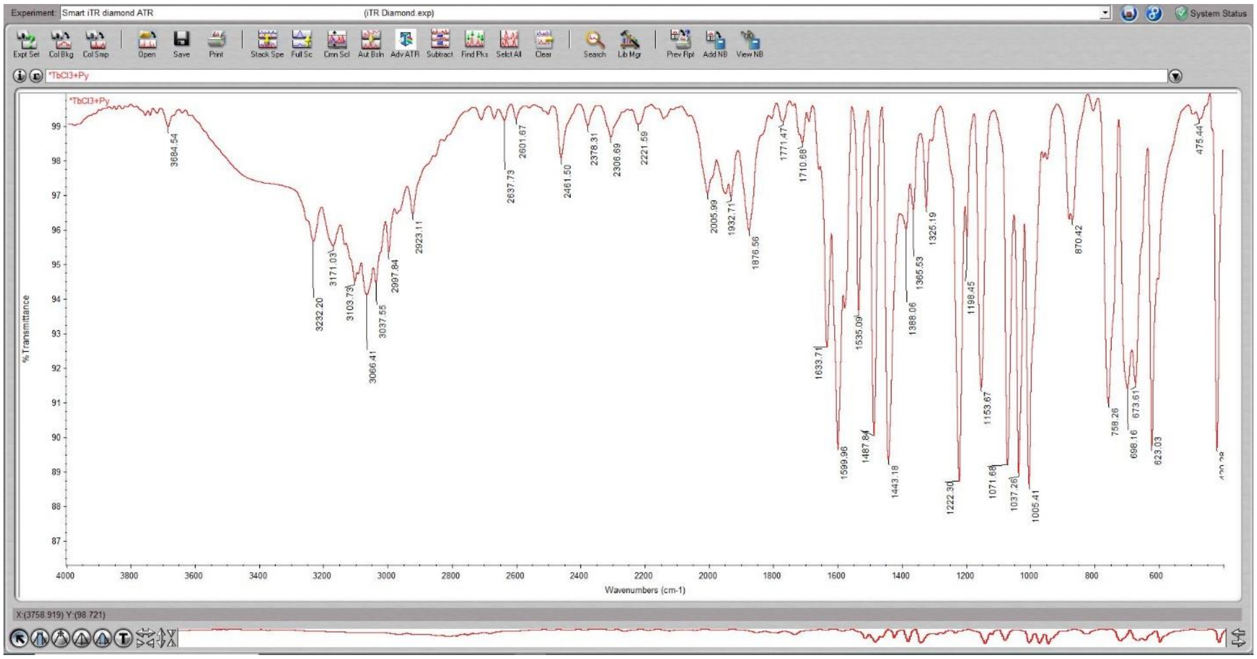

Figure S8: Analytical FTIR data for **[DyCl$_5$(py)]•2(H-py) (Dy-5)**

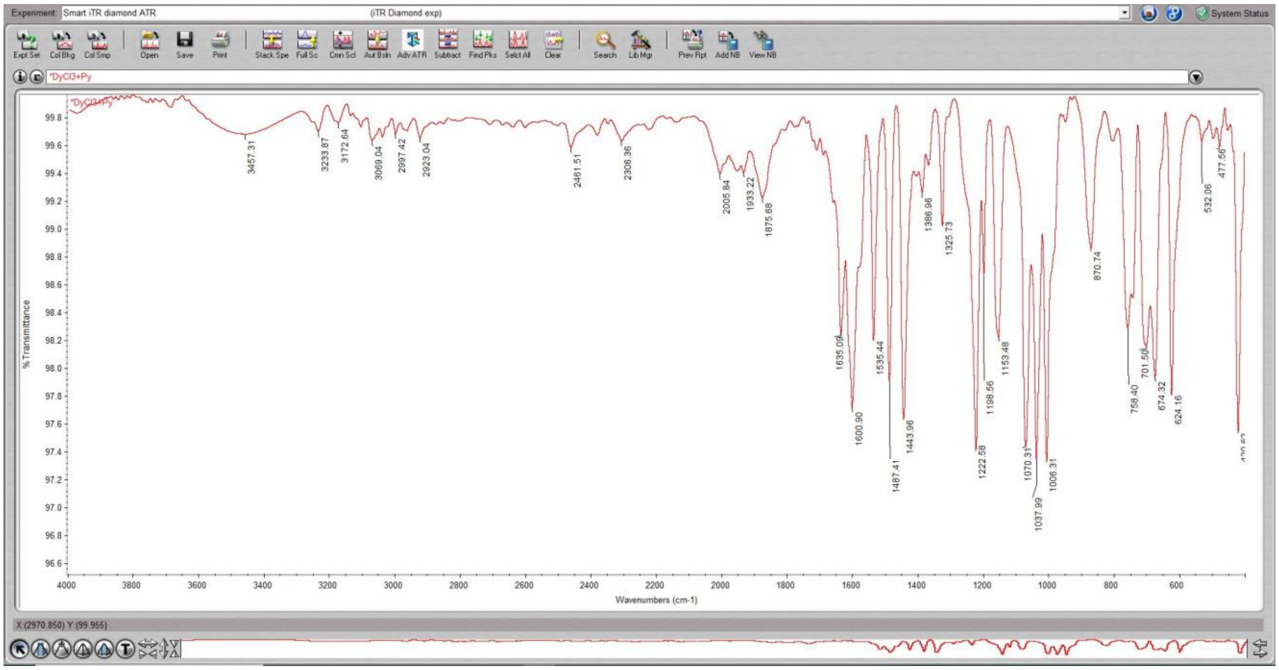

Figure S9: Analytical FTIR data for **[HoCl$_5$(py)]•2(H-py) (Ho-5)**

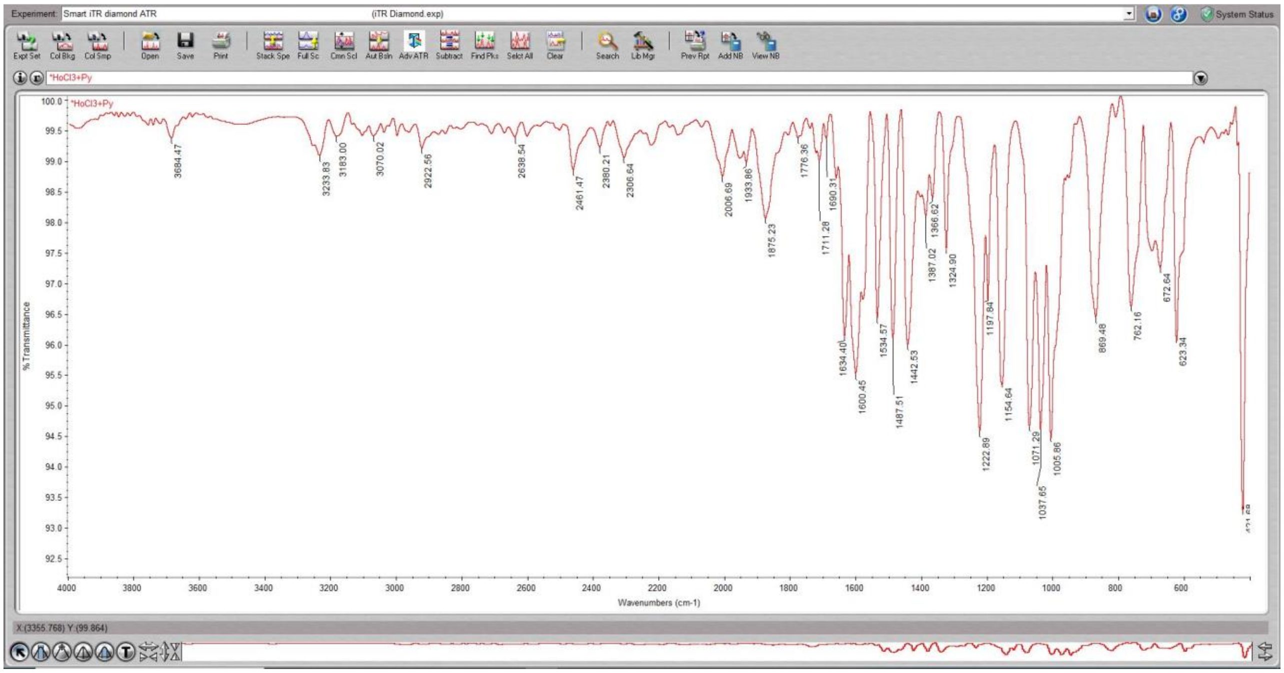

Figure S10: Analytical FTIR data for **[ErCl$_5$(py)]•2(H-py) (Er-5)**

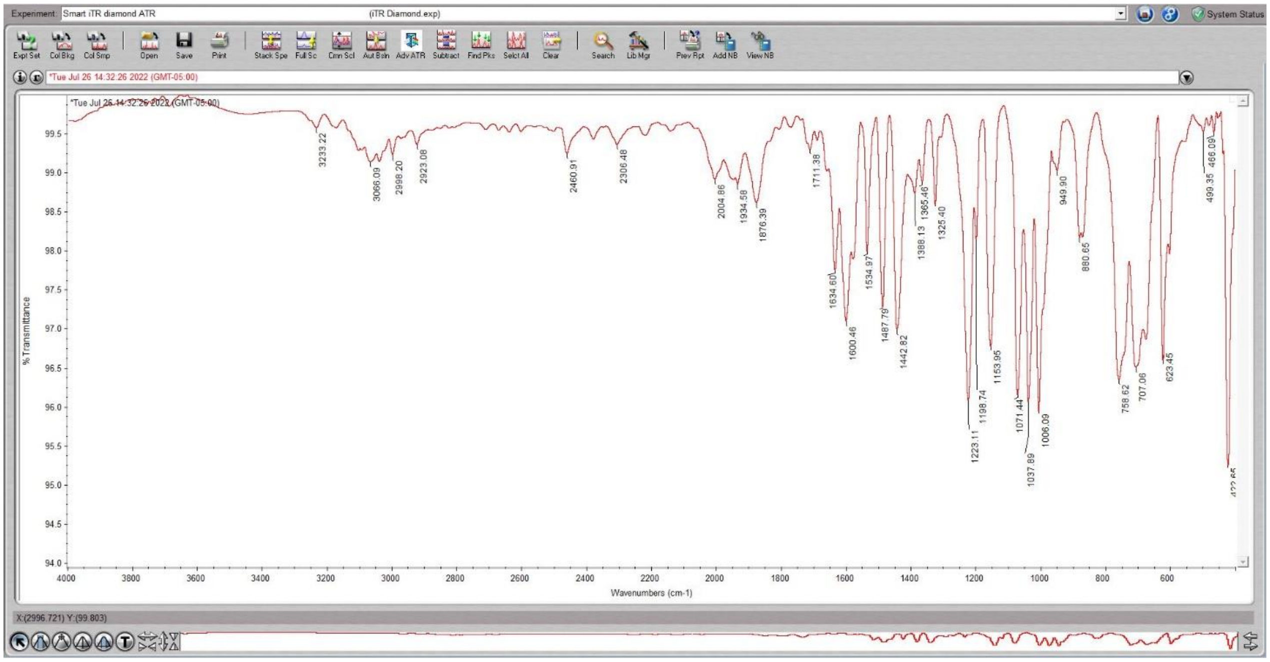

Figure S11: Analytical FTIR data for **[TmCl$_5$(py)]•2(H-py) (Tm-5)**

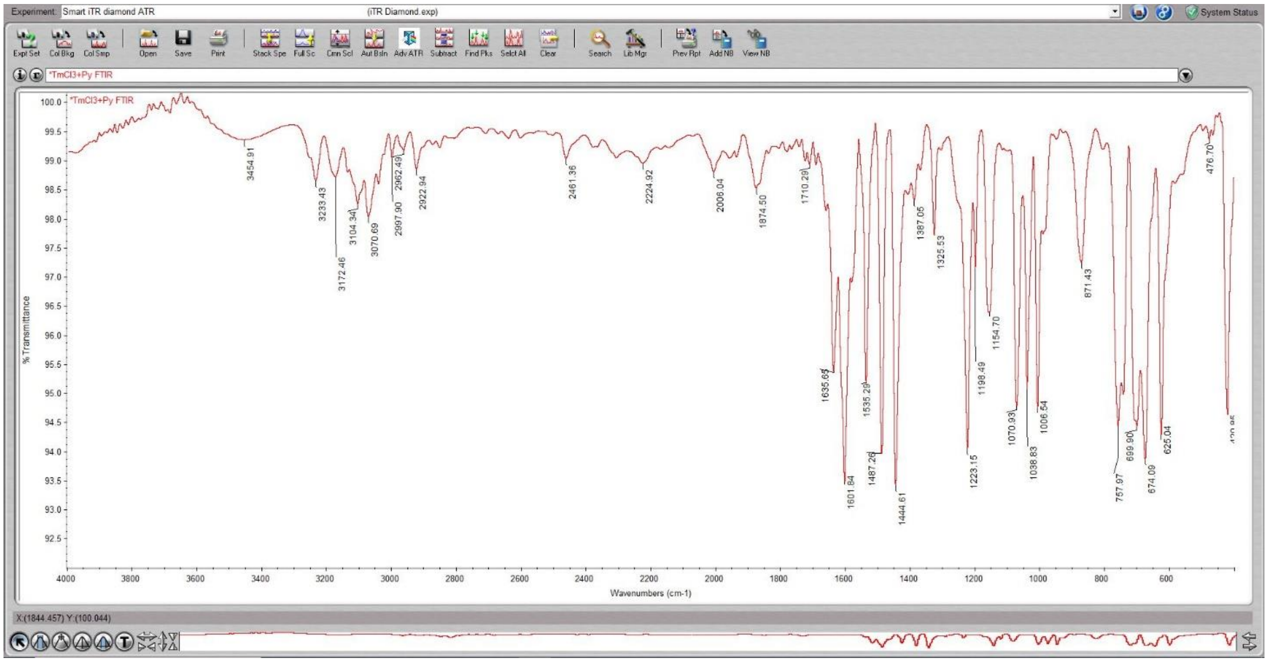

Figure S12: Analytical FTIR data for **[YbCl$_5$(py)]•2(H-py) (Yb-5)**

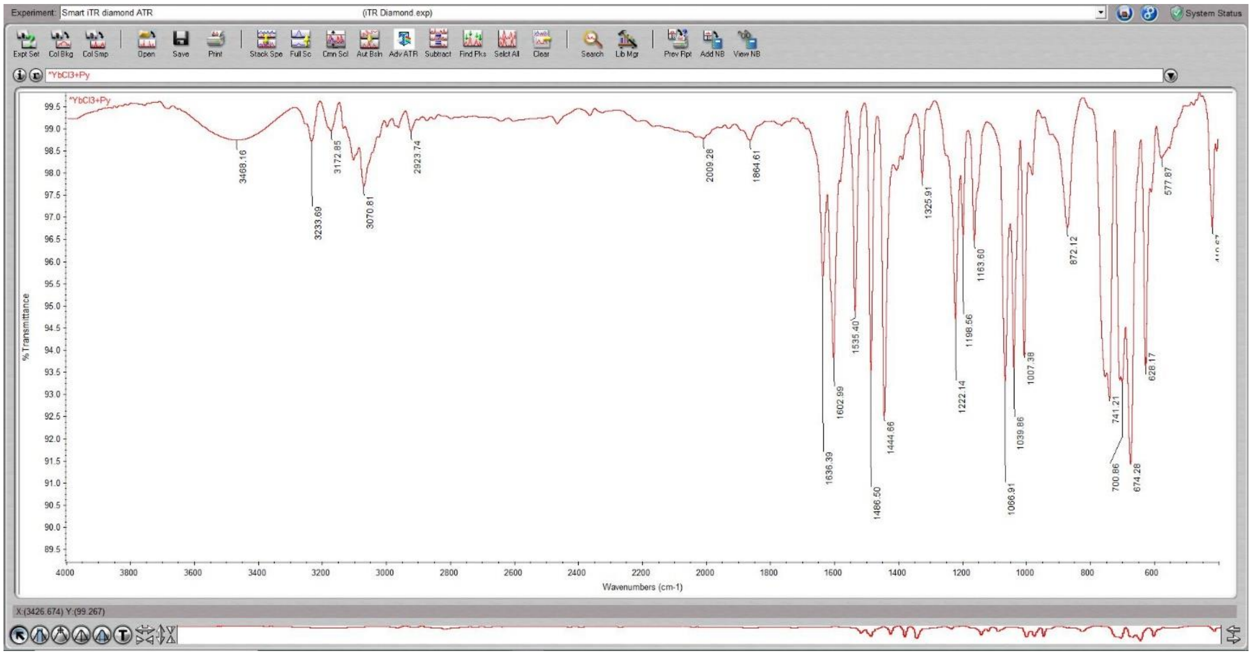

Figure S13: Analytical FTIR data for **[LuCl$_5$(py)]•2(H-py) (Lu-5)**

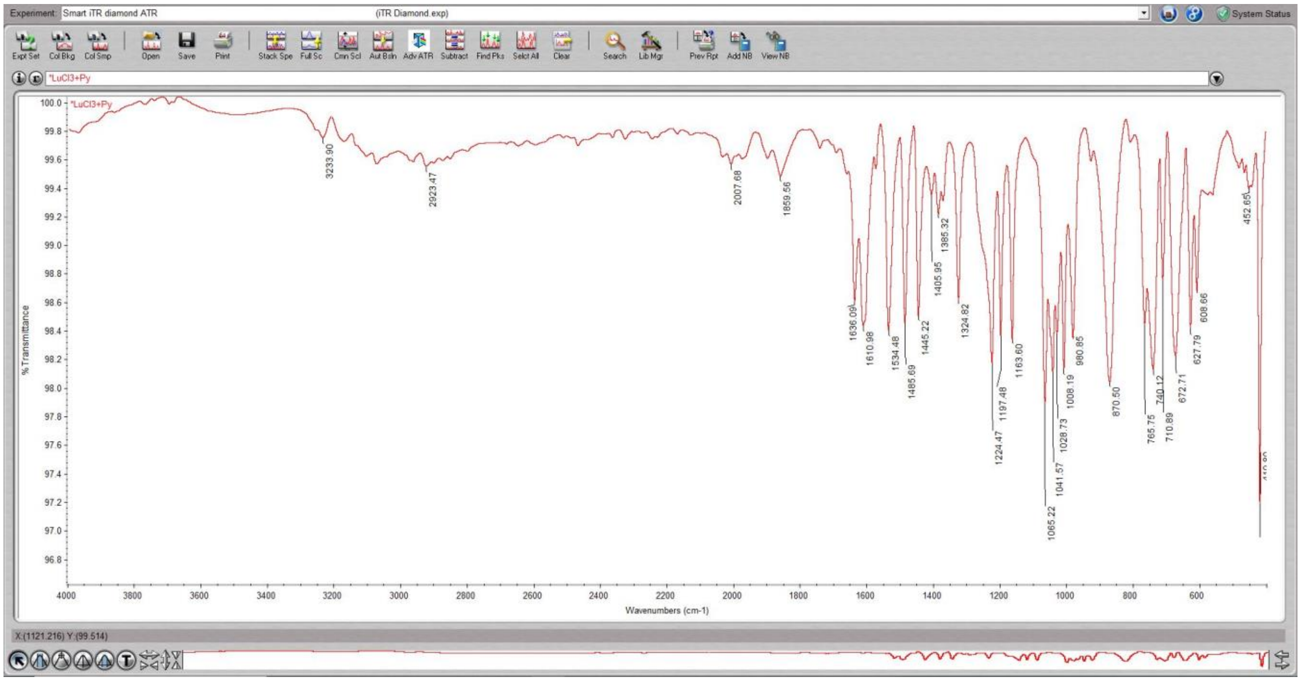

Figure S14: Analytical FTIR data for **[PrCl$_6$]•py-H-py, 2(H-py) (Pr-6).**

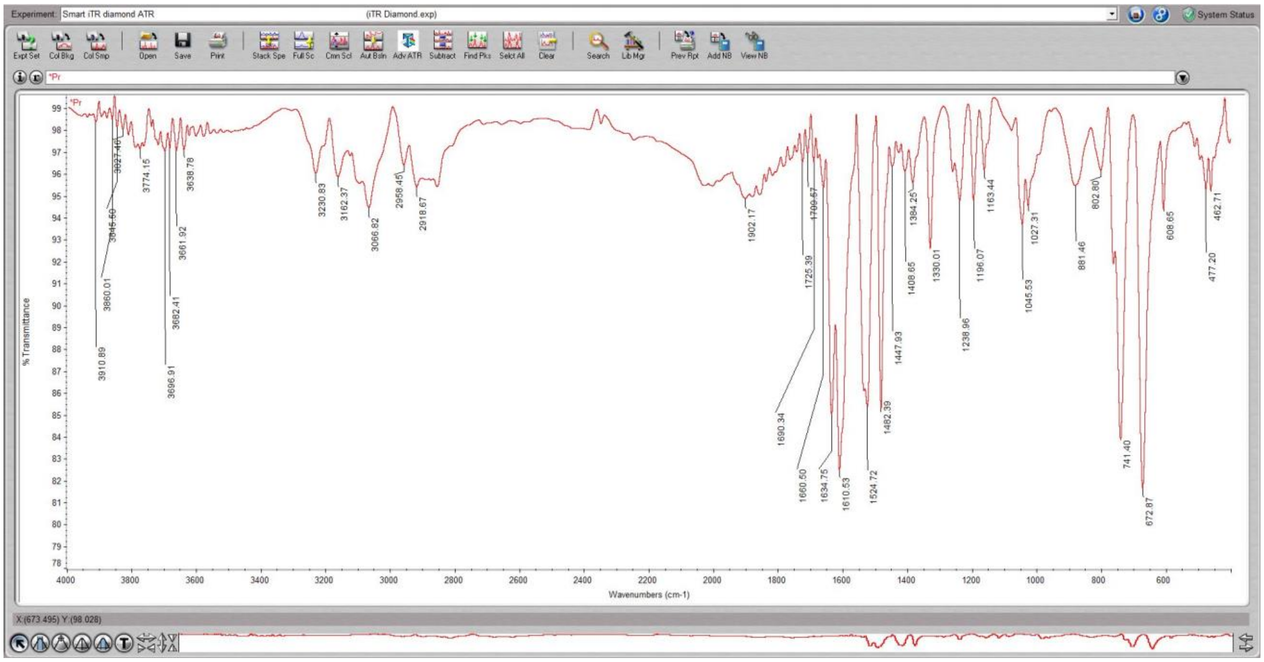

Figure S15: Theoretical PXRD data for **[LaCl₆]•py-H-py, 2(H-py) (La-6).**

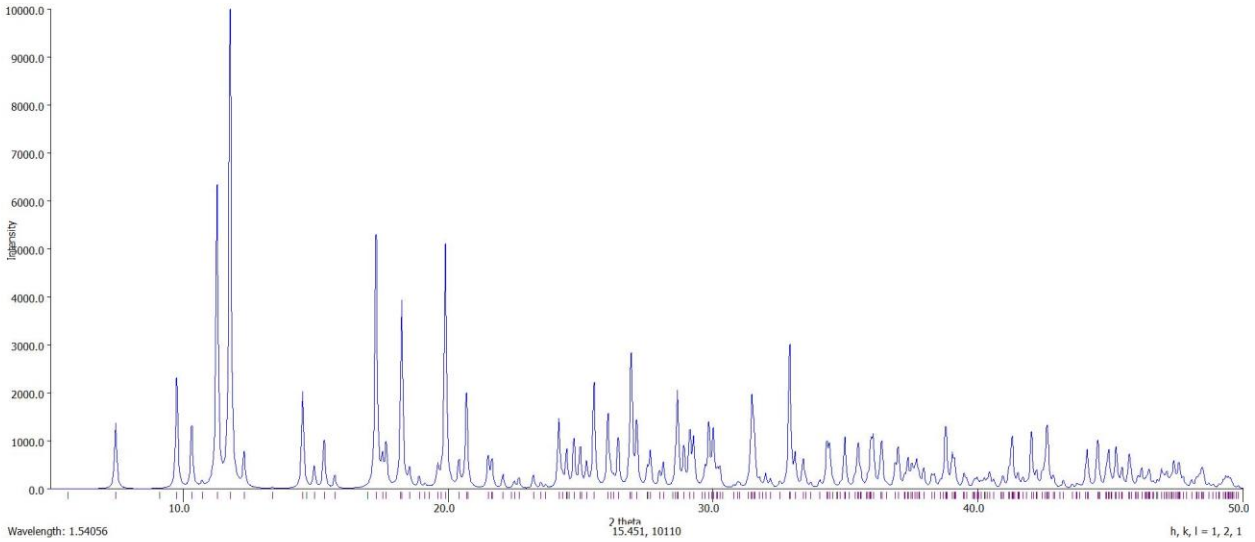

Figure S15.1: Experimental PXRD data for **[LaCl₆]•py-H-py, 2(H-py) (La-6).**

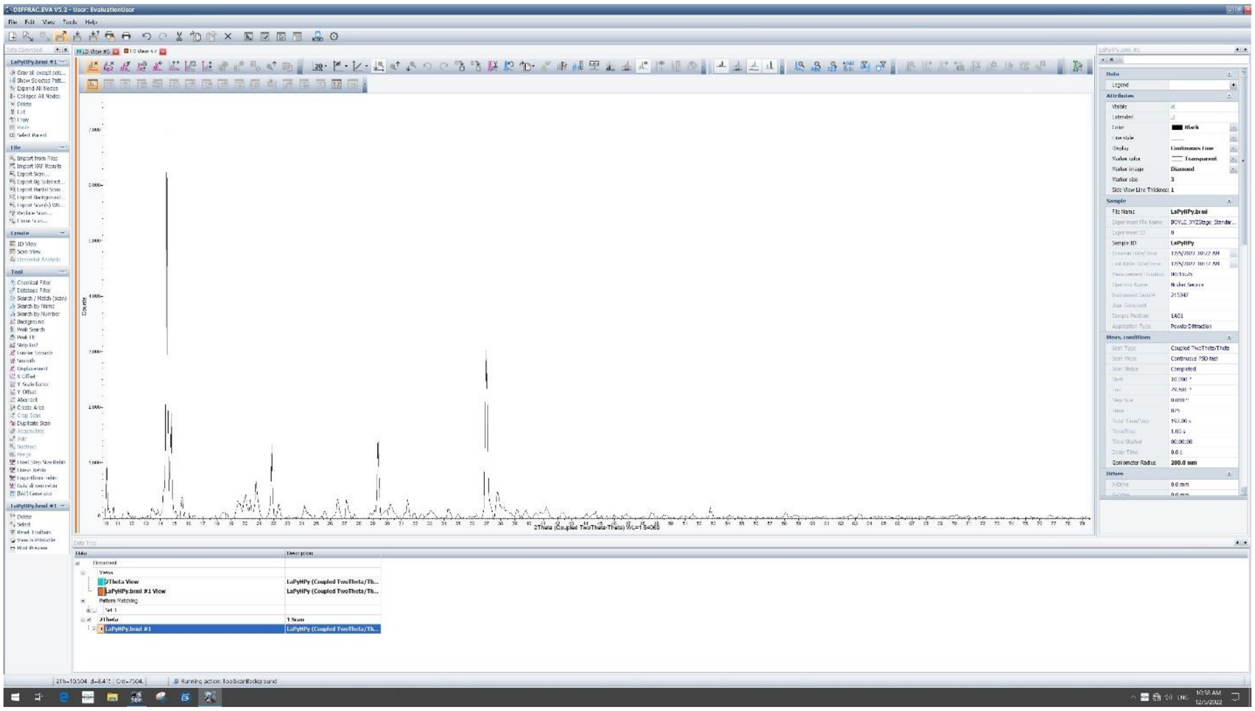

Figure S16: Theoretical PXRD data for **$[CeCl_6]$•py-H-py, 2(H-py) (Ce-6).**

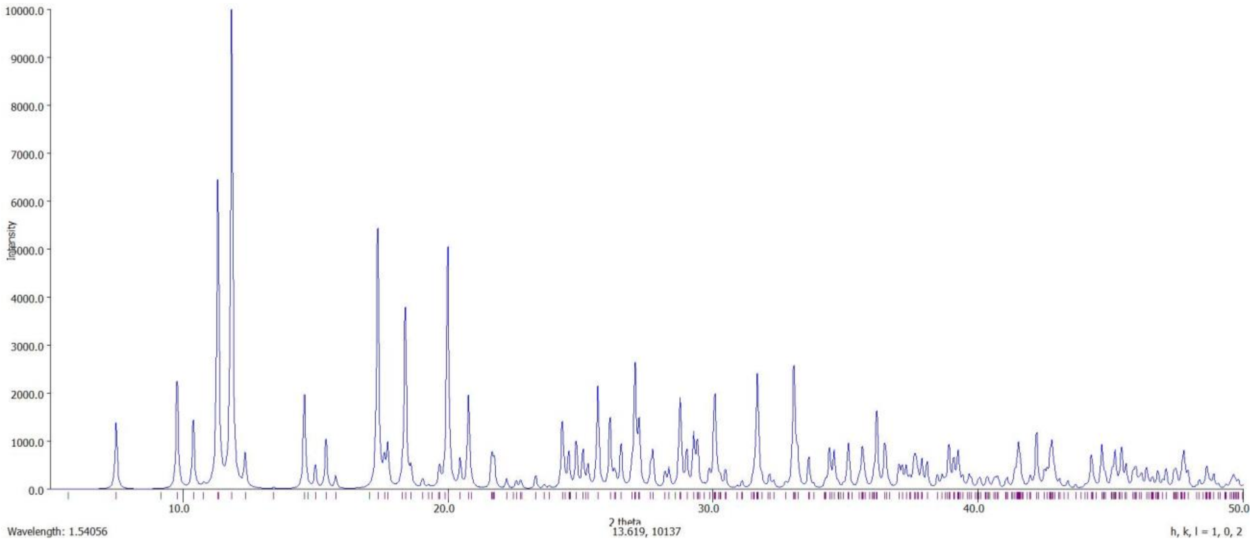

Figure S16.1: Experimental PXRD data for **$[CeCl_6]$•py-H-py, 2(H-py) (Ce-6).**

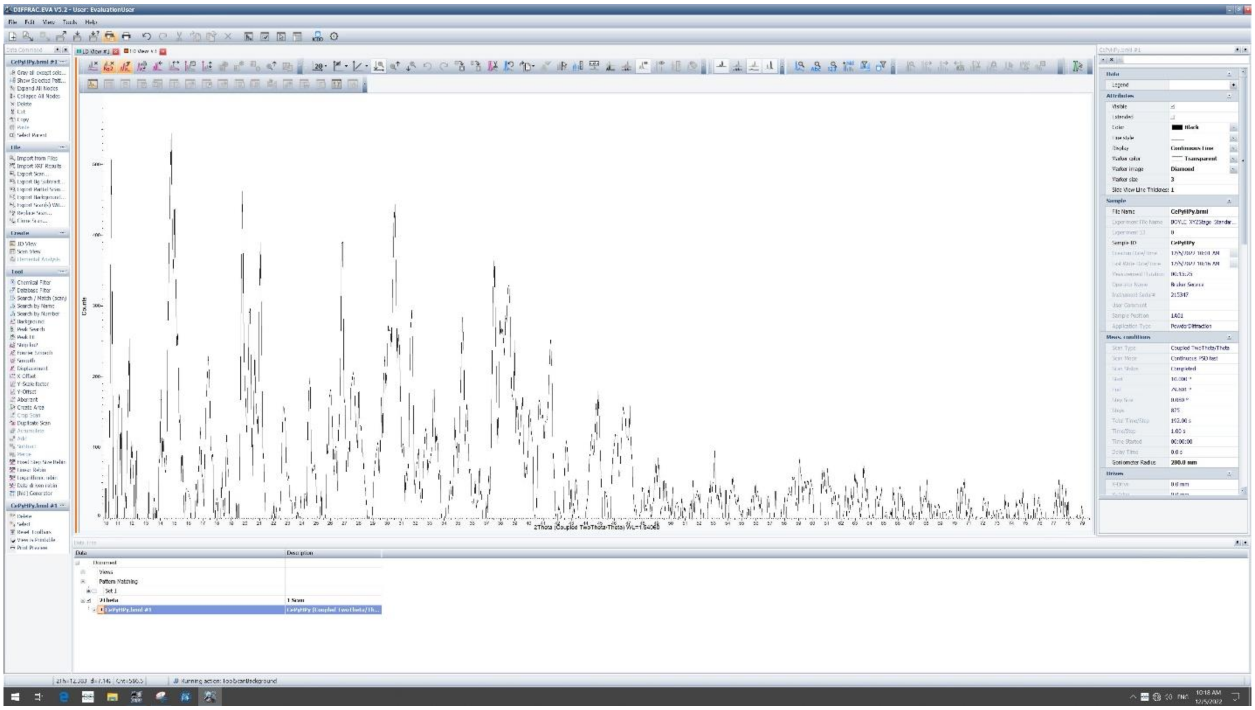

Figure S17: Theoretical PXRD data for **[NdCl$_6$]•py-H-py, 2(H-py) (Nd-6).**

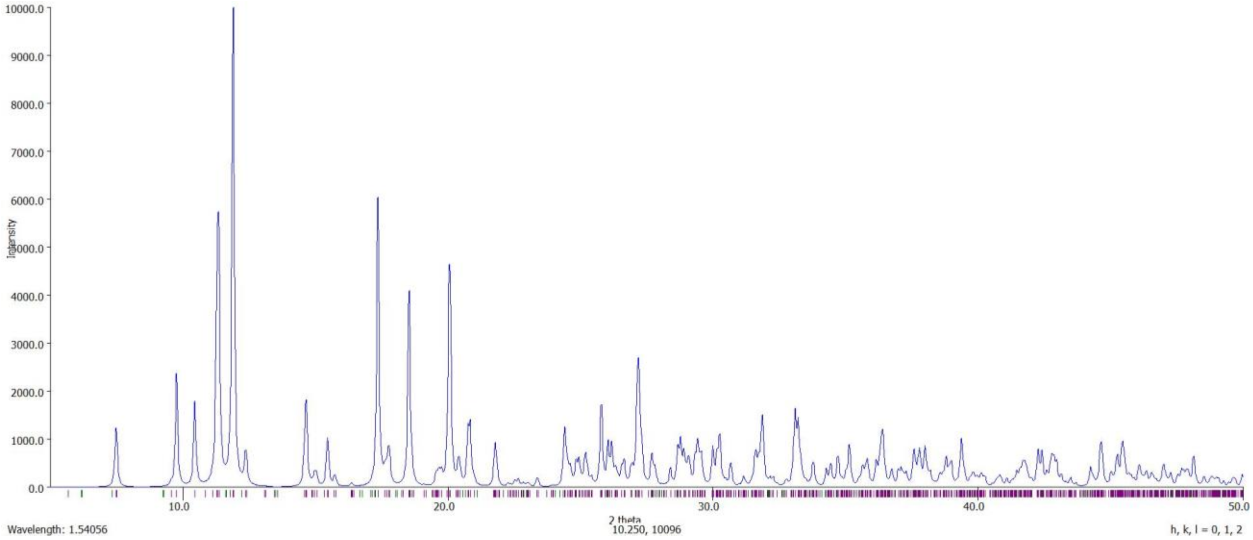

Figure S17.1: Experimental PXRD data for **[NdCl$_6$]•py-H-py, 2(H-py) (Nd-6).**

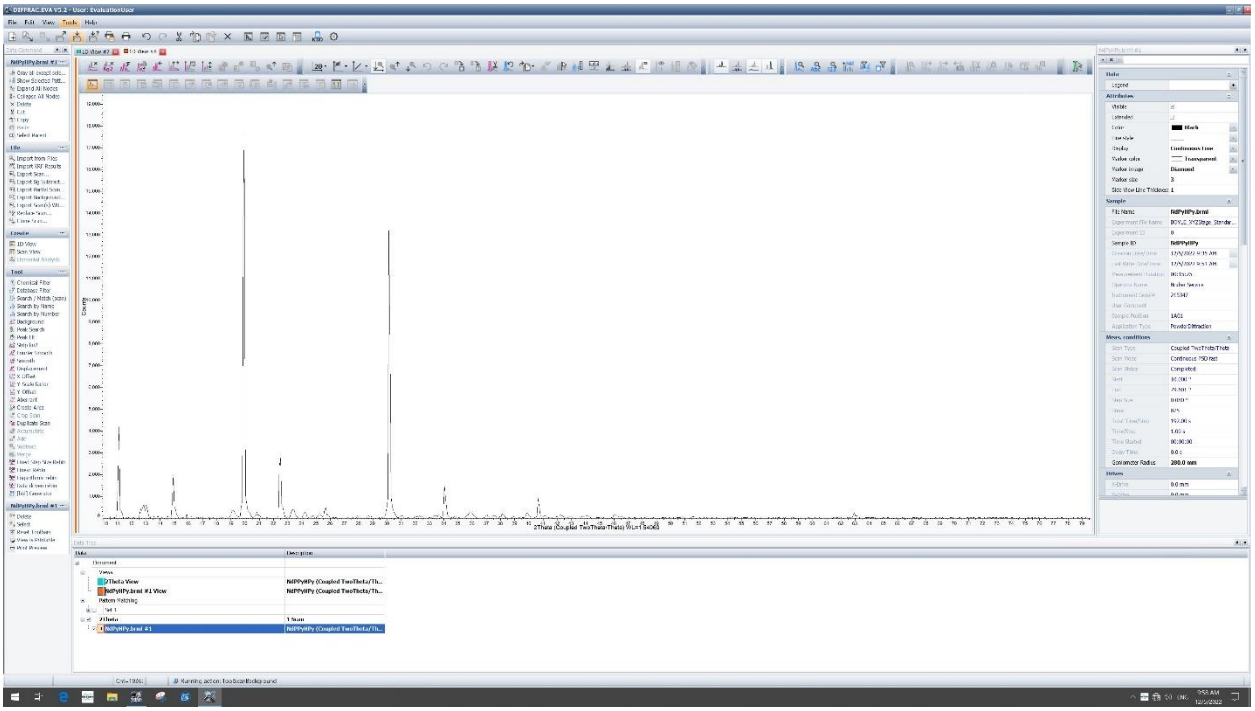

Figure S18: Theoretical PXRD data for **[SmCl$_6$]•py-H-py, 2(H-py) (Sm-6).**

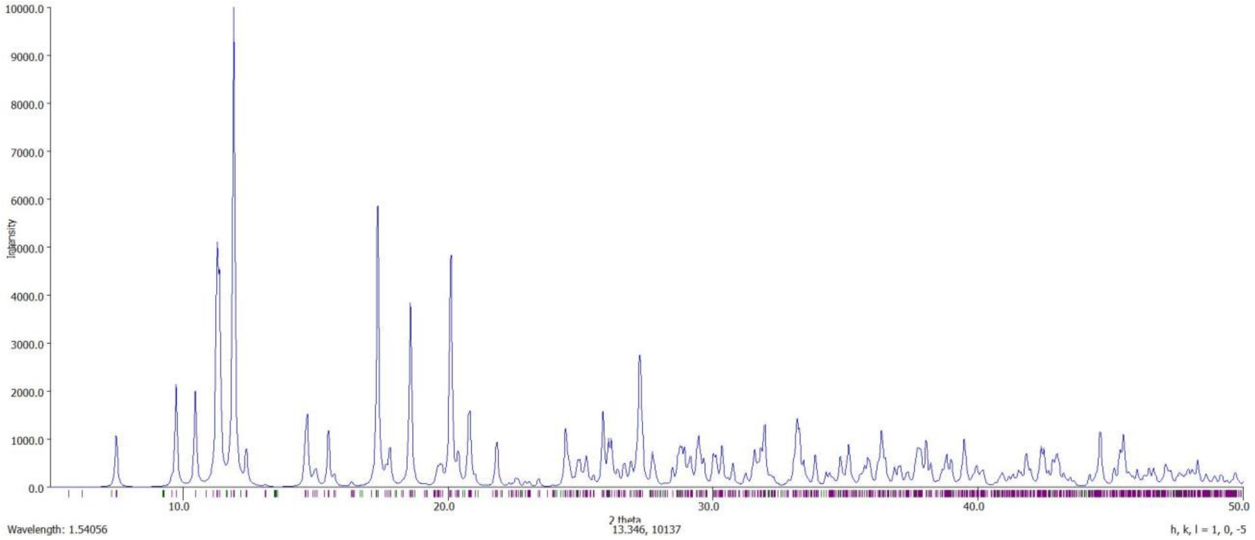

Figure S18.1: Experimental PXRD data for **[SmCl$_6$]•py-H-py, 2(H-py) (Sm-6).**

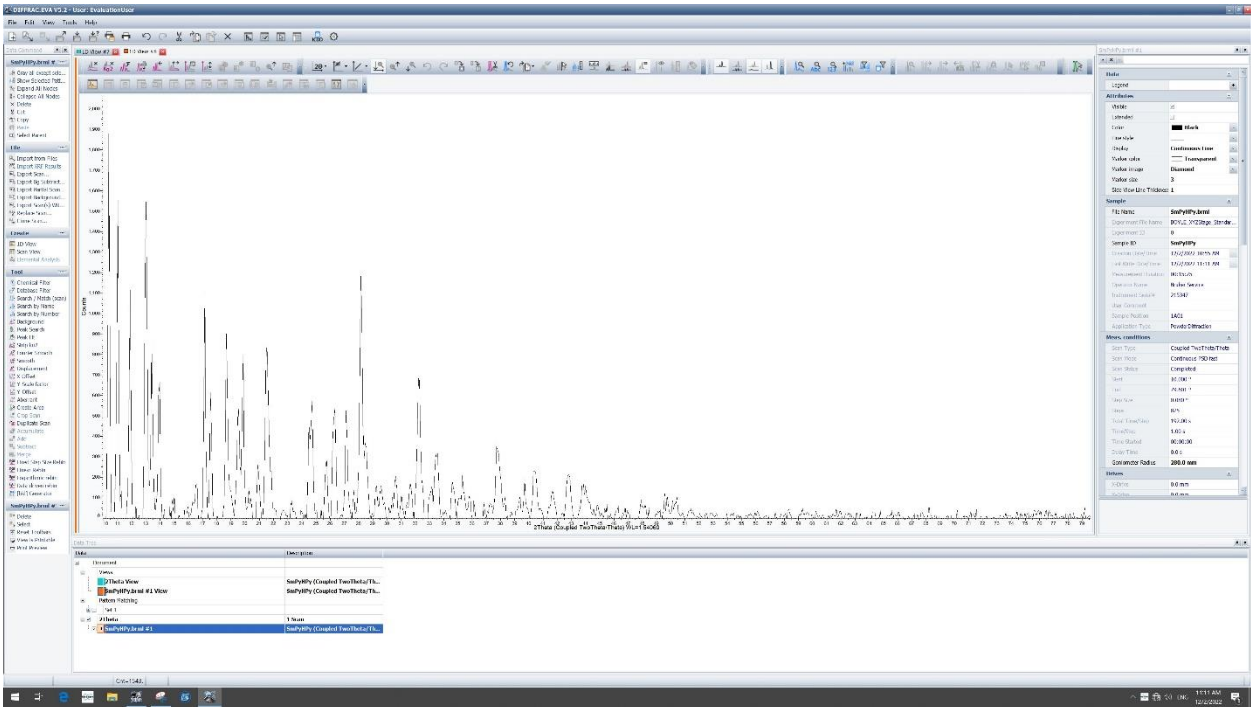

Figure S19: Theoretical PXRD data for **[EuCl$_6$]•py-H-py, 2(H-py) (Eu-6).**

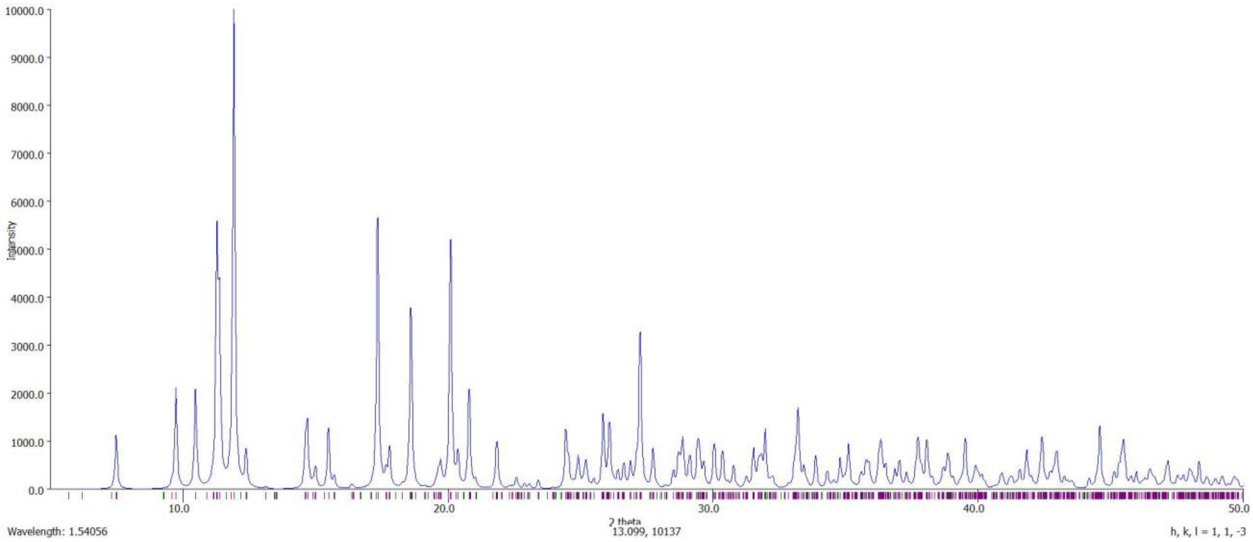

Figure S19.1: Experimental PXRD data for **[EuCl$_6$]•py-H-py, 2(H-py) (Eu-6).**

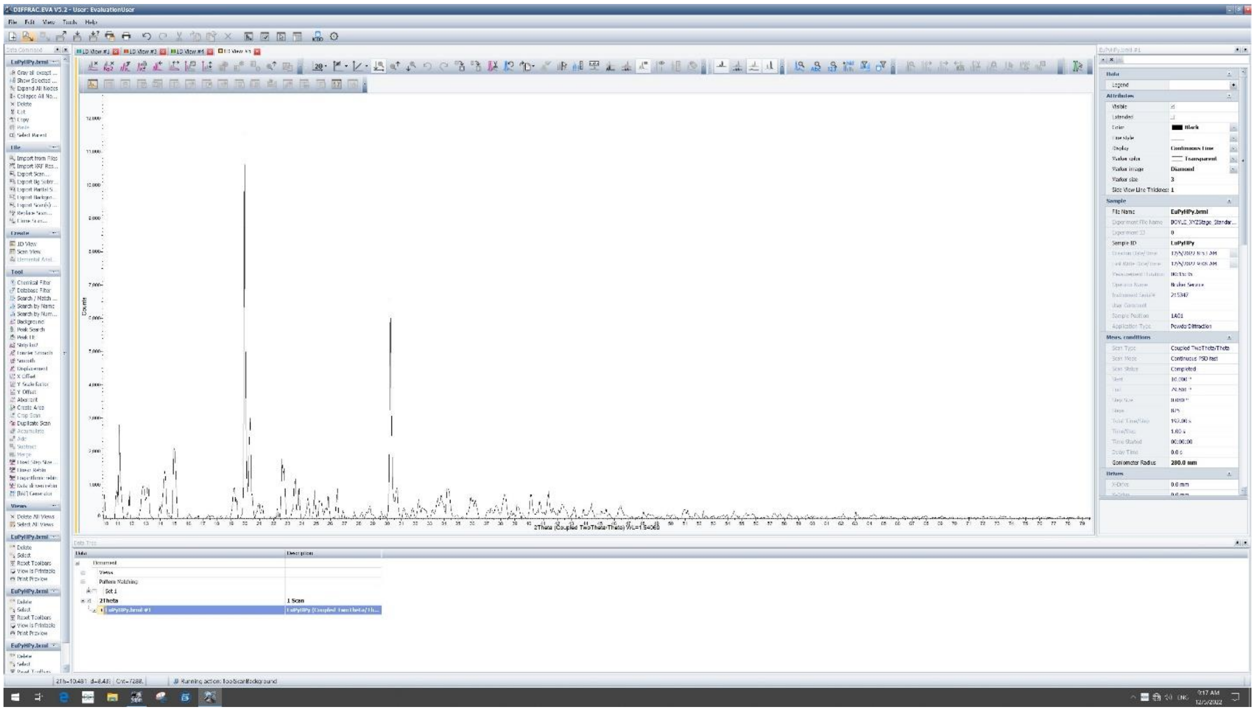

Figure S20: Theoretical PXRD data for **[GdCl$_6$]•py-H-py, 2(H-py) (Gd-6).**

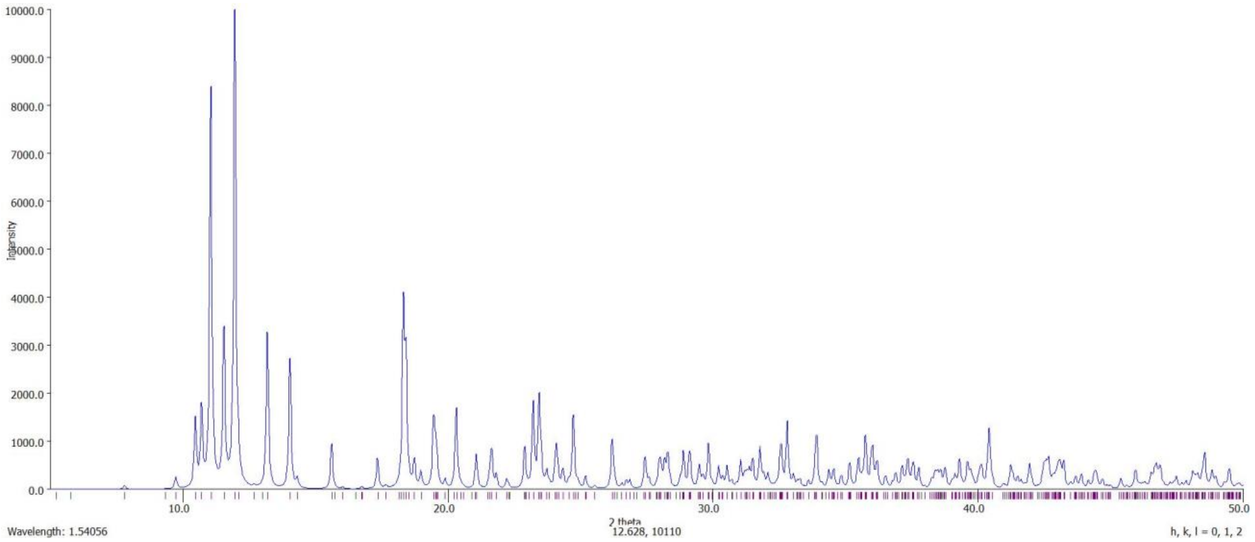

Figure S20.1: Experimental PXRD data for **[GdCl$_6$]•py-H-py, 2(H-py) (Gd-6).**

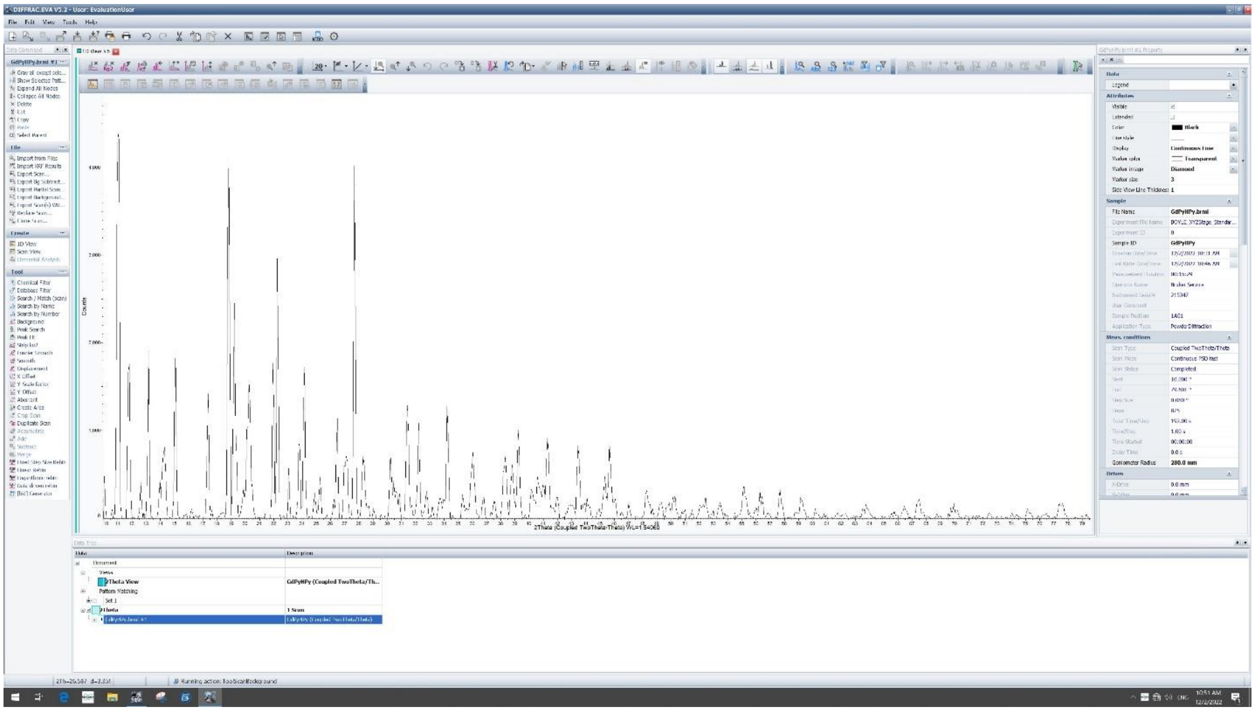

Figure S21: Theoretical PXRD data for **[TbCl$_5$(py)]•2(H-py) (Tb-5).**

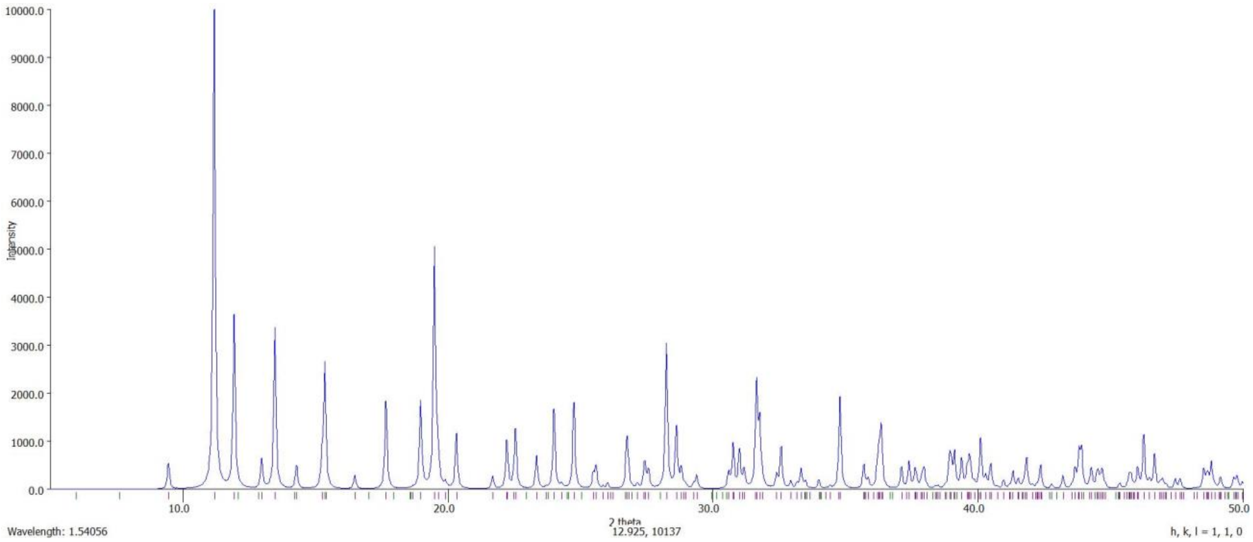

Figure S21.1: Experimental PXRD data for **[TbCl$_5$(py)]•2(H-py) (Tb-5).**

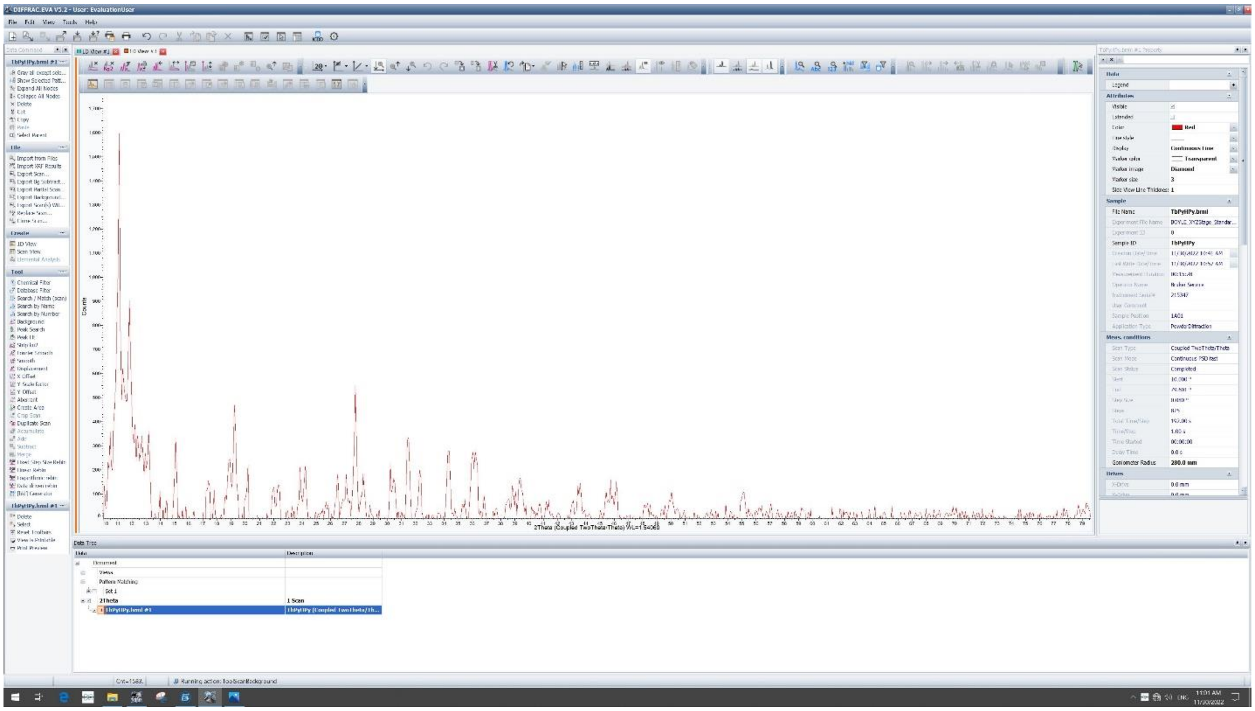

Figure S22: Theoretical PXRD data for **[DyCl5(py)]•2(H-py) (Dy-5).**

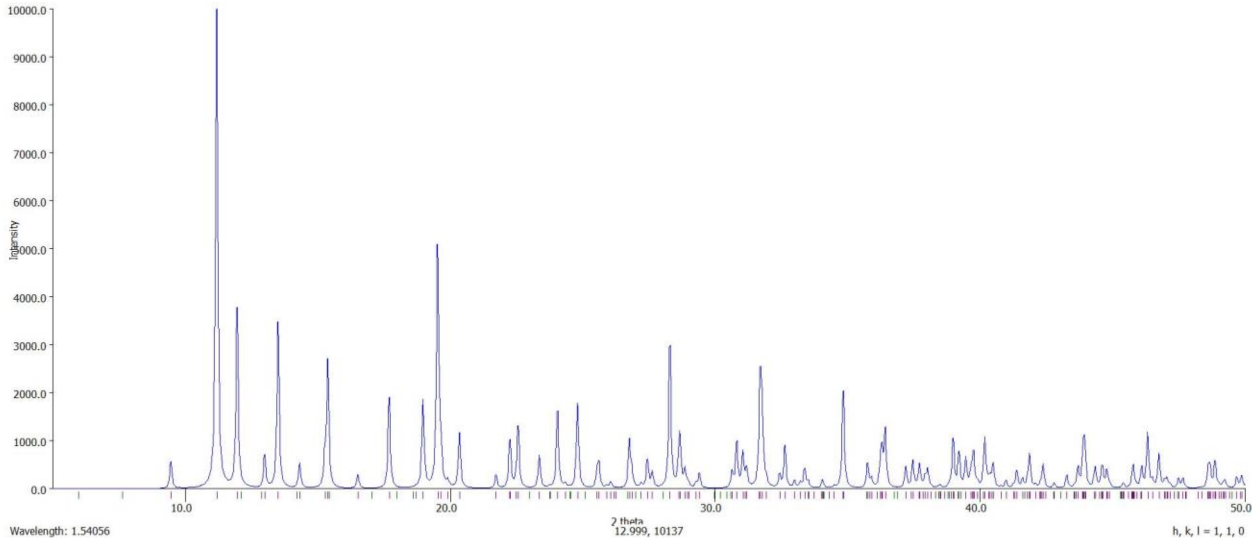

Figure S22.1: Experimental PXRD data for **[DyCl5(py)]•2(H-py) (Dy-5).**

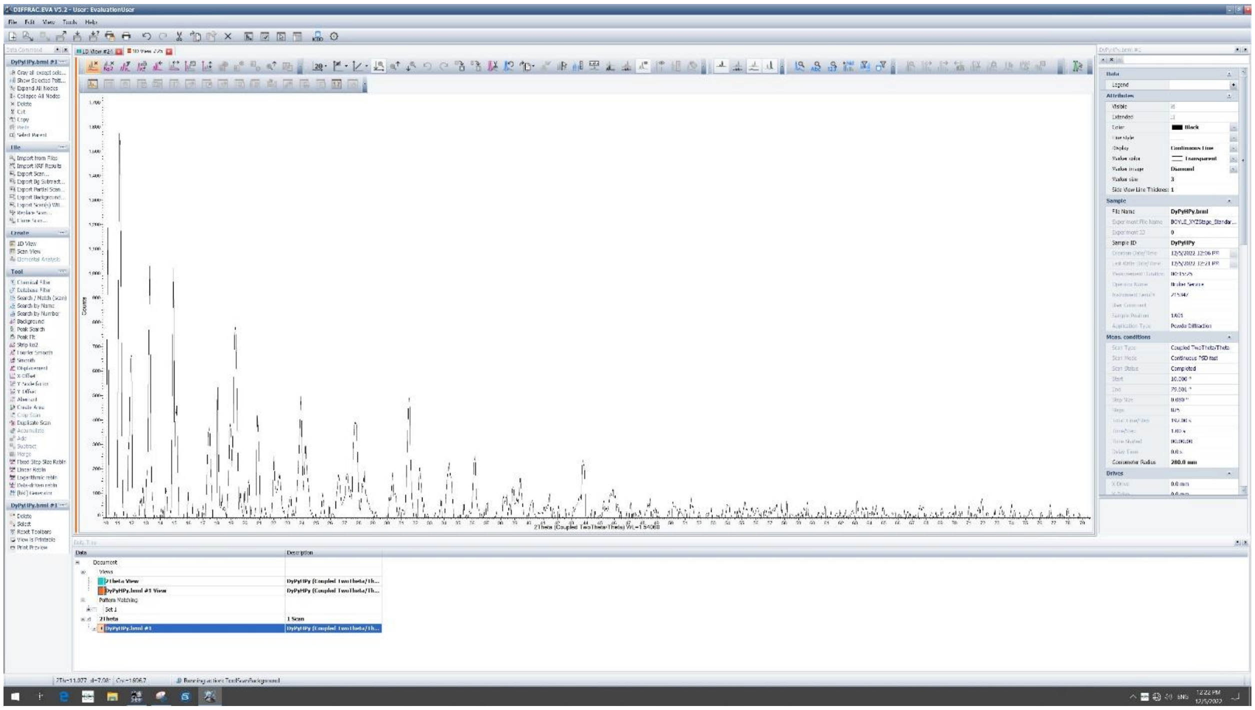

Figure S23: Theoretical PXRD data for **[HoCl₅(py)]•2(H-py) (Ho-5).**

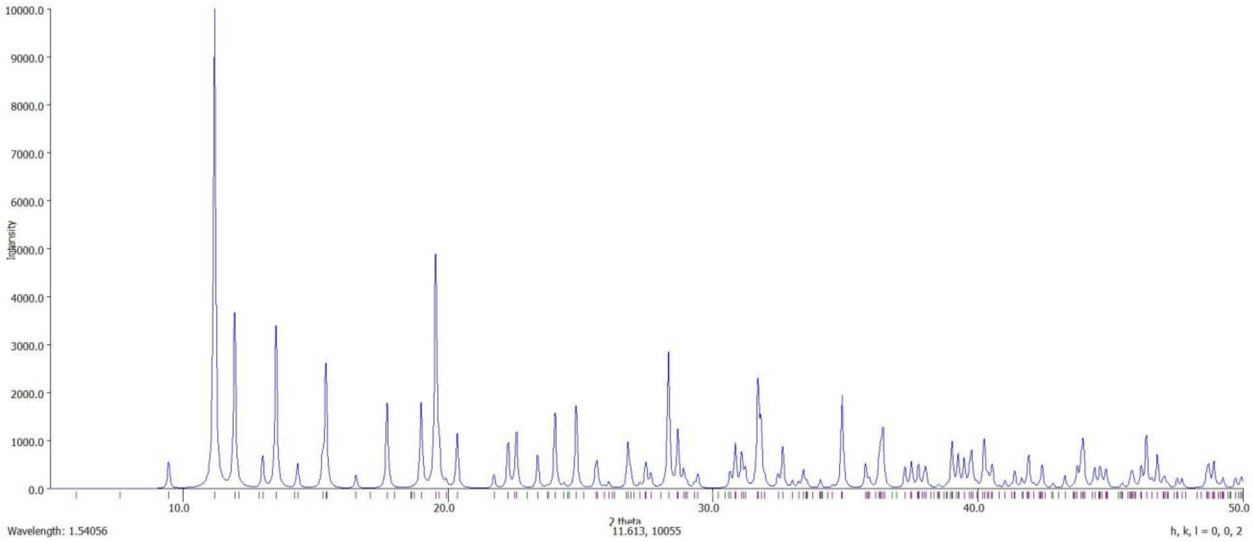

Figure S23.1: Experimental PXRD data for **[HoCl₅(py)]•2(H-py) (Ho-5).**

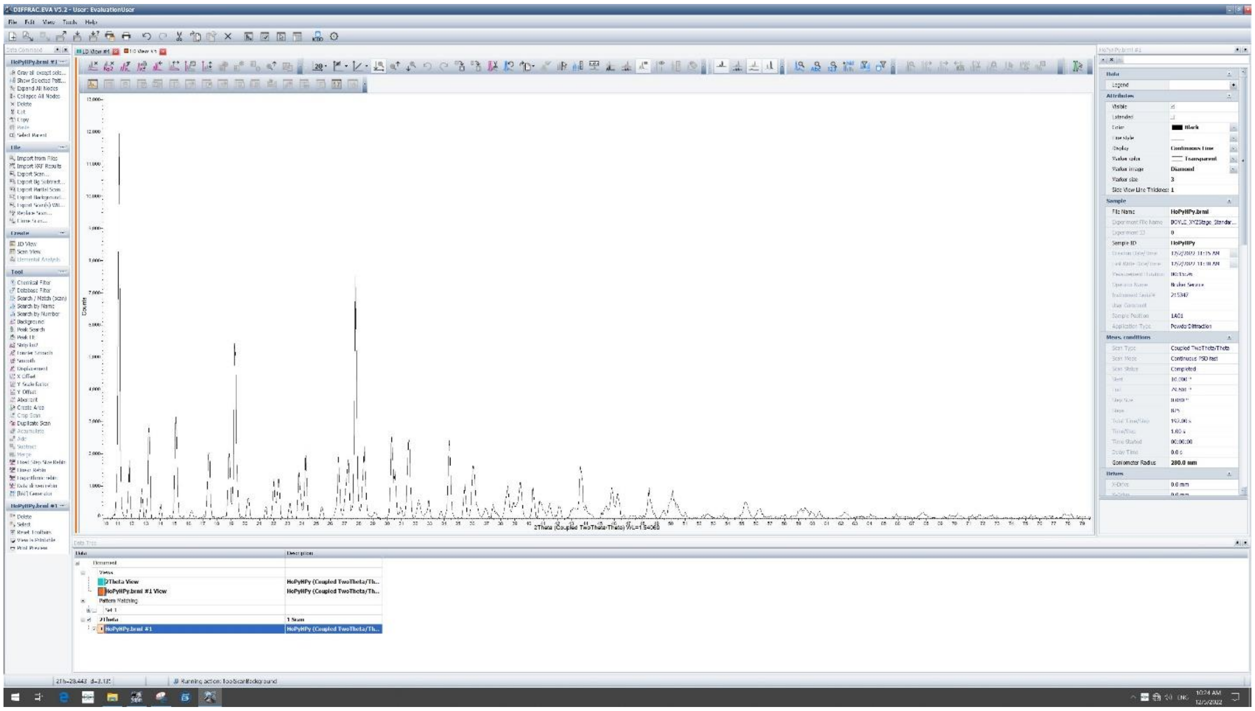

Figure S24: Theoretical PXRD data for **[ErCl$_5$(py)]•2(H-py) (Er-5).**

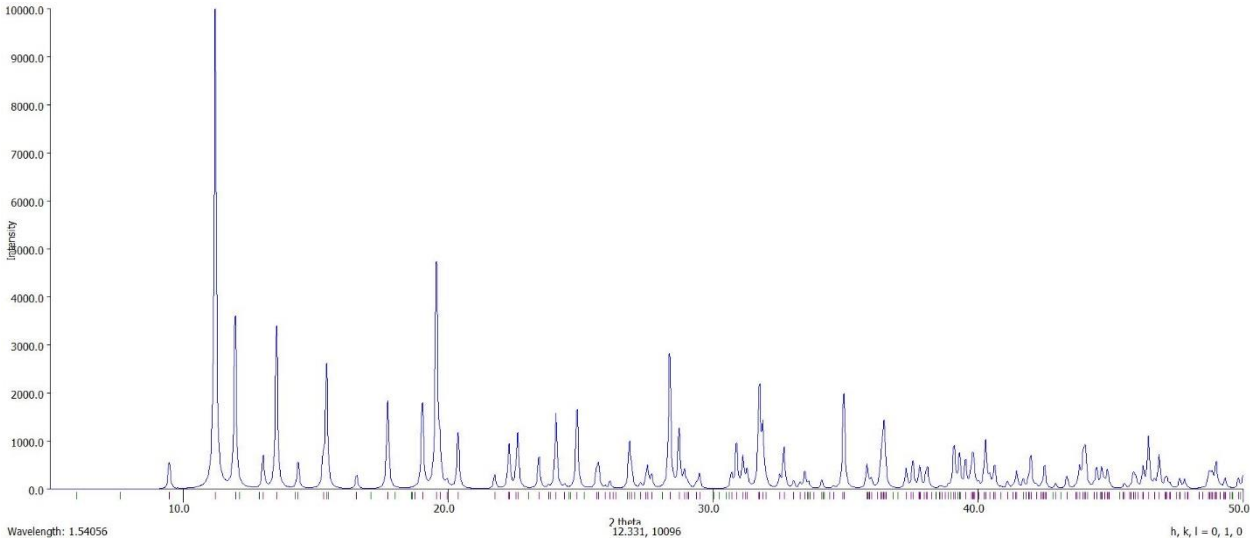

Figure S24.1: Experimental PXRD data for **[ErCl$_5$(py)]•2(H-py) (Er-5).**

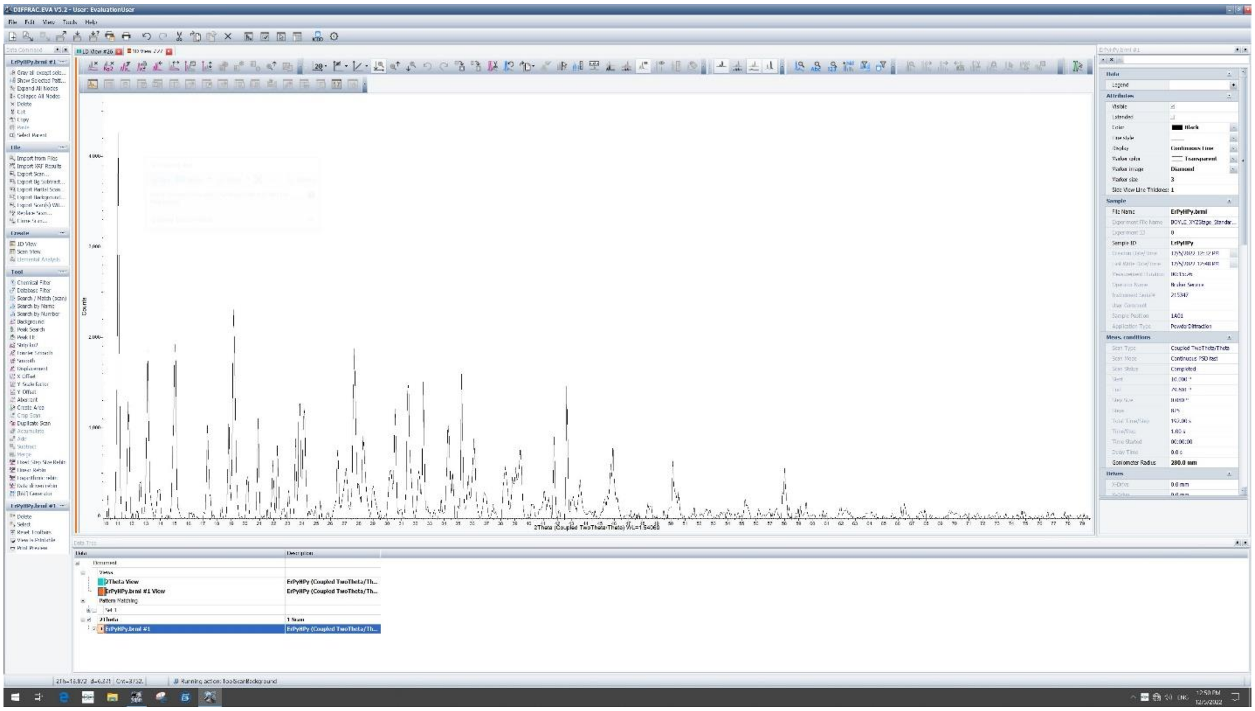

Figure S25: Theoretical PXRD data for **[$TmCl_5$(py)]•2(H-py) (Tm-5).**

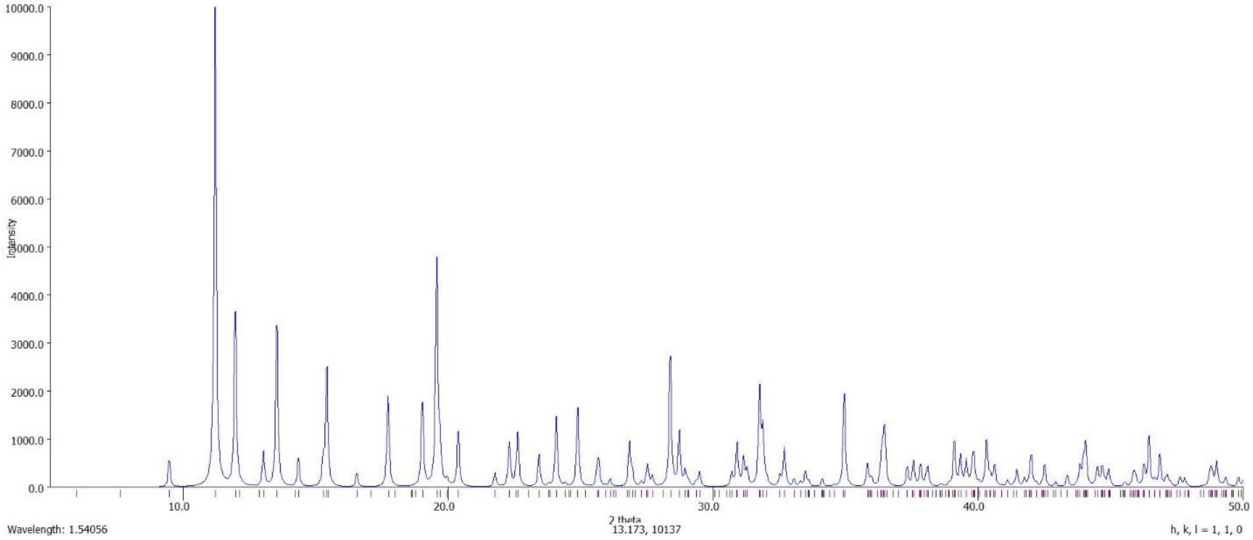

Figure S25.1: Experimental PXRD data for **[$TmCl_5$(py)]•2(H-py) (Tm-5).**

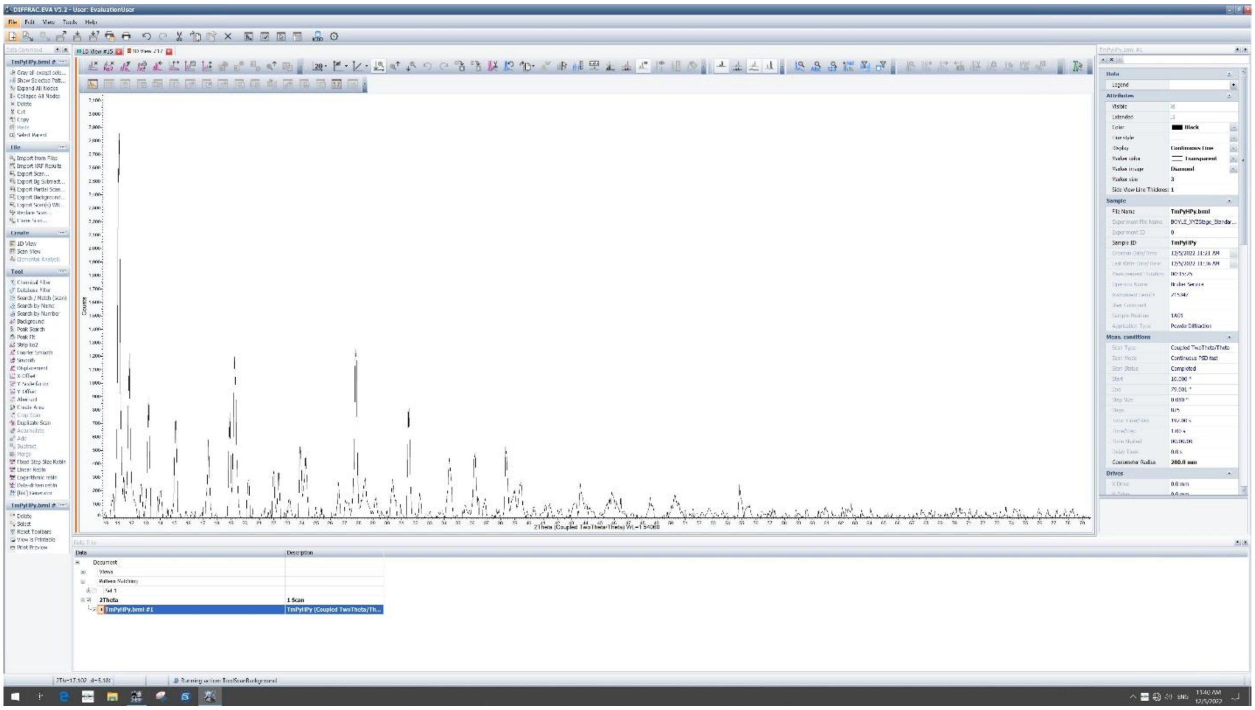

Figure S26: Theoretical PXRD data for **[YbCl$_5$(py)]•2(H-py) (Yb-5).**

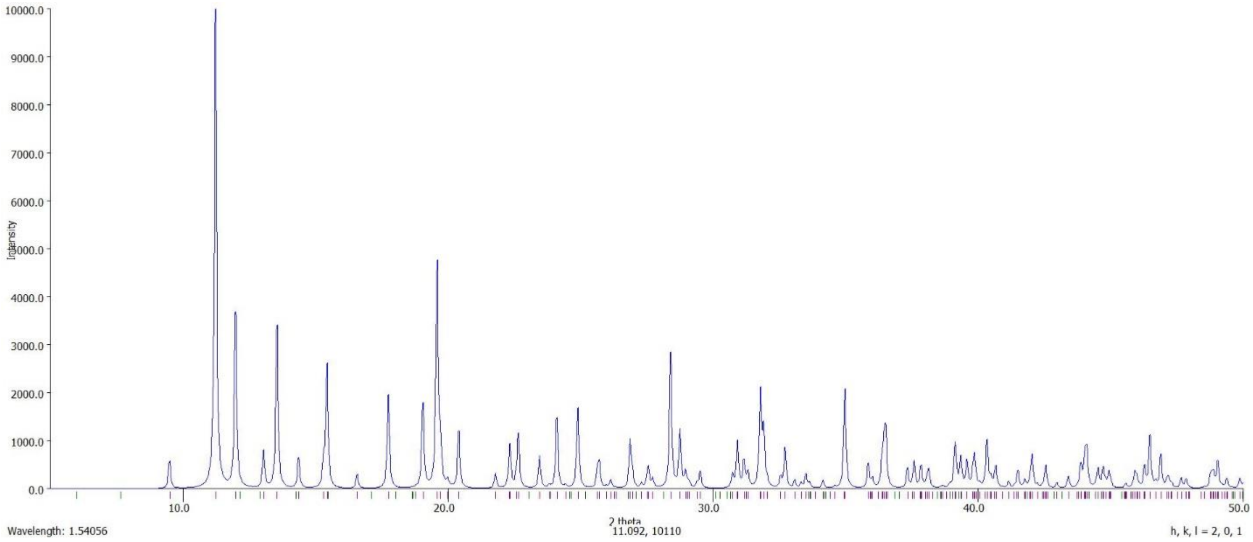

Figure S26.1: Experimental PXRD data for **[YbCl$_5$(py)]•2(H-py) (Yb-5).**

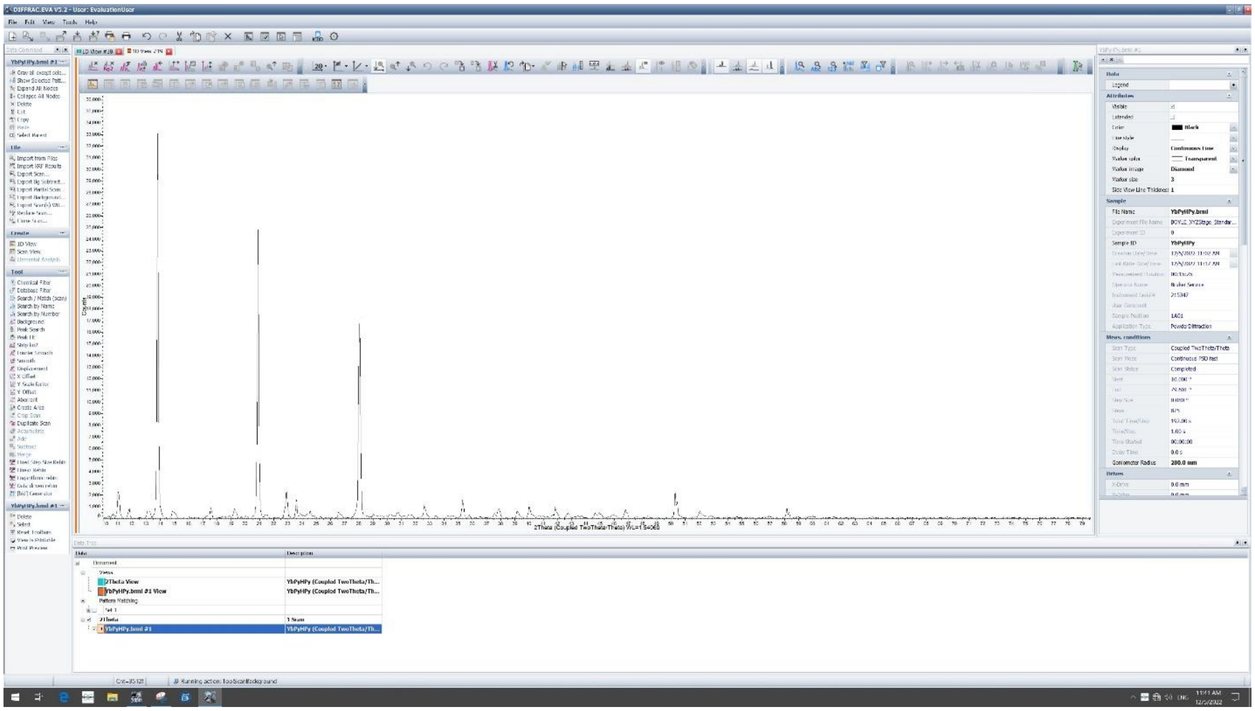

Figure S27: Theoretical PXRD data for **[LuCl$_5$(py)]•2(H-py) (Lu-5).**

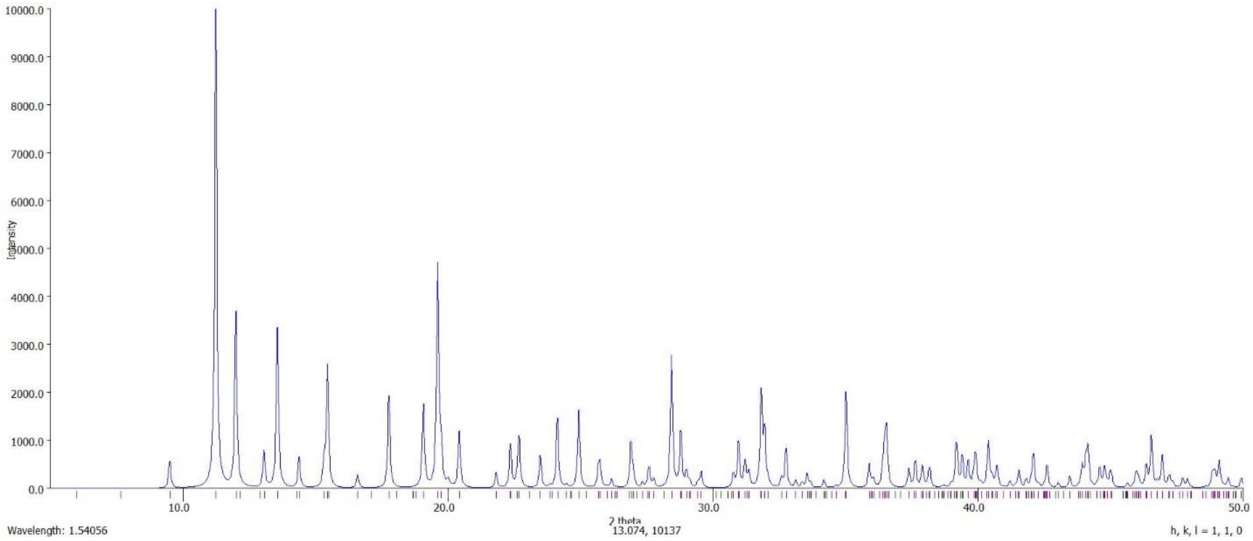

Figure S27.1: Experimental PXRD data for **[LuCl$_5$(py)]•2(H-py) (Lu-5).**

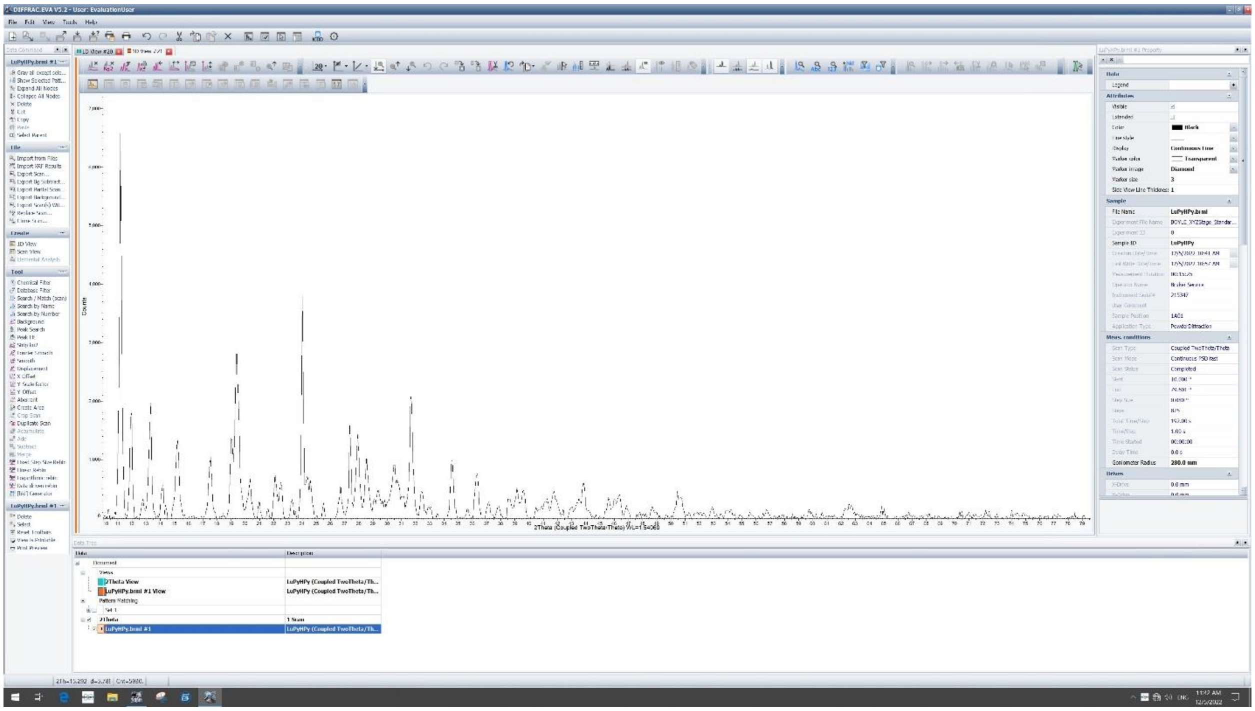

Figure S28: Theoretical PXRD data for **[PrCl$_6$]•py-H-py, 2(H-py) (Pr-6).**

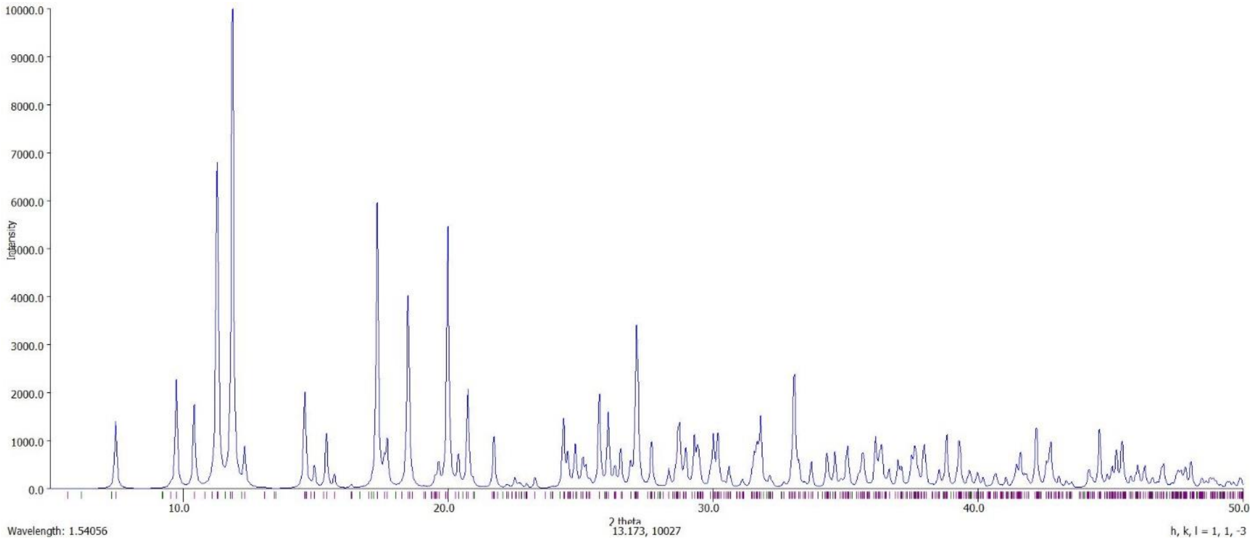

Figure S28.1: Experimental PXRD data for **[PrCl$_6$]•py-H-py, 2(H-py) (Pr-6).**

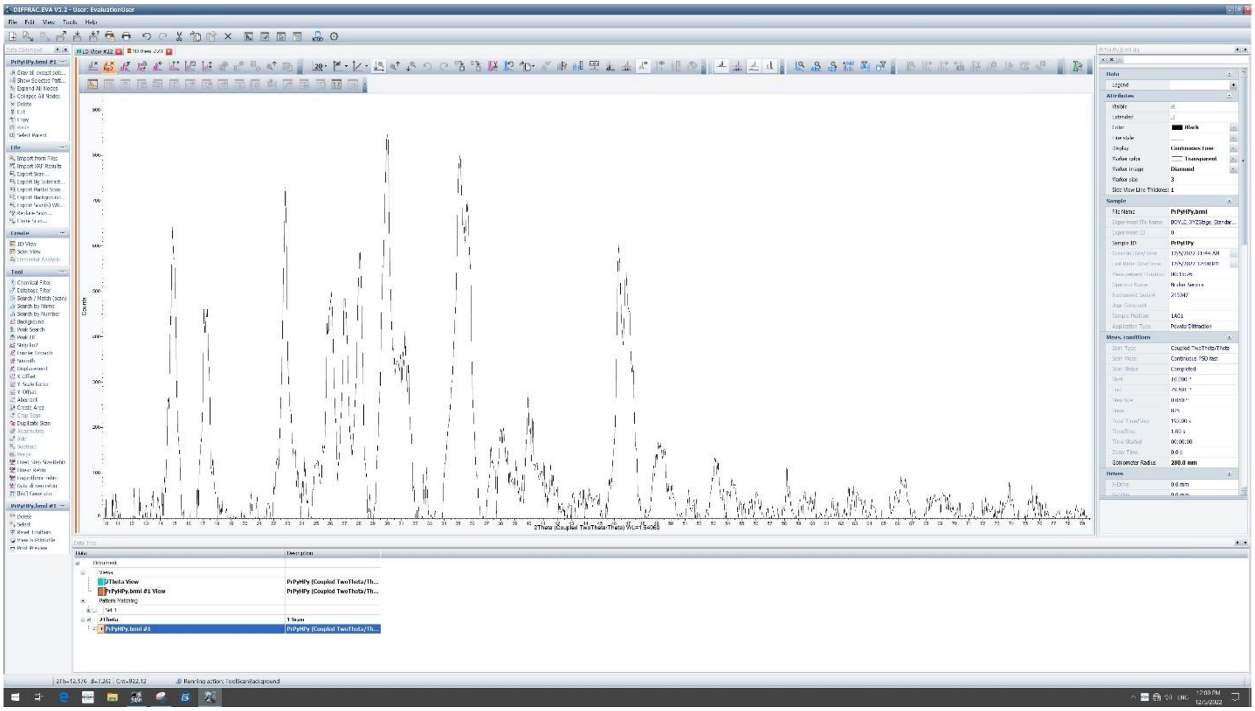

List of Tables.

Table S1. Metrical data for **Ln-6**. All distances are in Å.

Table S2. Metrical data for **Ln-5**. All distances are in Å.

Table S3. Cation-cation contacts in **Ln-6** structures.

Table S4. C-H•••Cl hydrogen bonds in the **Ln-5** structures.

Table S5. C-H•••Cl Hydrogen bonds in the **Pr-6** structure

Table S6. Coulumbic energies (Kcal/mol) for **Nd-6**, **Gd-6**, **Tb-5**, and **Lu-5**.

Table S7. F-test results **for Ln-5** species.

Table S1. Metrical data for **Ln-6**. All distances are in Å.

Ln-Cl	La	Ce	Pr	Nd	Sm	Eu	Gd
Ln(1)-Cl(11)	2.786	2.749	2.726	2.709	2.694	2.689	2.659
Ln(1)-Cl(12)	2.831	2.800	2.756	2.749	2.72	2.708	2.662
Ln(1)-Cl(13)	2.770	2.735	2.699	2.681	2.647	2.639	2.676
Ln(1)-Cl(14)	2.810	2.764	2.707	2.711	2.679	2.667	2.683
Ln(1)-Cl(15)	2.799	2.753	2.750	2.755	2.743	2.73	2.721
Ln(1)-Cl(16)	2.781	2.745	2.751	2.725	2.702	2.698	2.662
Ln(2)-Cl(21)			2.715	2.69	2.672	2.664	2.693
Ln(2)-Cl(22)			2.720	2.714	2.665	2.674	2.694
Ln(2)-Cl(23)			2.798	2.774	2.735	2.728	2.705
Ln(2)-Cl(24)			2.728	2.721	2.661	2.671	2.677
Ln(2)-Cl(25)			2.744	2.732	2.705	2.699	2.622
Ln(2)-Cl(26)			2.750	2.732	2.711	2.702	2.661
**H bonds**							
N(10)-N(20)	2.678	2.678	2.651	2.683	2.666	2.676	2.691
N(30)-N(40)			2.694	2.665	2.66	2.681	2.662
N(50)-Cl(12)	3.181	3.190	3.178	3.145	3.115	3.12	3.139

N(60)-Cl(15)			3.288	3.214	3.18	3.201	3.226
N(70)-Cl(25)			3.154	3.149	3.179	3.182	3.178
N(70)-Cl(15)	3.147	3.151					
N(80)-Cl(23)			3.221	3.208	3.181	3.182	3.180
**Ionic Contacts**							
N(50)-Cl(11)			3.719	3.762	3.706	3.683	3.542
N(50)-Cl(15)			3.780	3.79	3.721	3.713	3.622
N(50-Cl(16)	3.345	3.333					
N(60)-Cl(11)			3.367	3.357	3.333	3.329	3.349
N(60)-Cl(14)			4.257	4.226	4.171	4.167	4.110
N(50)-Cl(14)	4.227	4.186					
N(70)-Cl(24)			3.713	3.693	3.595	3.568	3.672
N(70)-Cl(14)	3.668	3.667					
N(70)-Cl(26)			3.641	3.69	3.600	3.597	3.712
N(70)-Cl(16)	3.564	3.574					
N(80)-Cl(24)			3.358	3.351	3.322	3.335	3.312
N(80)-Cl(26)			4.153	4.129	4.144	4.134	4.147

Table S2. Metrical data for **Ln-5**. All distances are in Å.

**Ln-Cl**	**Tb**	**Dy**	**Ho**	**Er**	**Tm**	**Yb**	**Lu**
Ln(1)-Cl(1)	2.652	2.645	2.635	2.619	2.610	2.620	2.591
Ln(1)-Cl(2)	2.628	2.611	2.607	2.591	2.579	2.571	2.562
Ln(1)-Cl(3)	2.639	2.626	2.622	2.606	2.595	2.597	2.579
Ln(1)-Cl(4)	2.648	2.63	2.628	2.611	2.600	2.586	2.561
Ln(1)-Cl(5)	2.647	2.63	2.631	2.612	2.600	2.580	2.562
Ln-N(py)	2.524	2.517	2.507	2.486	2.475	2.475	2.454
**H bonds**							
N(20)-Cl(1)	3.200	3.182	3.186	3.190	3.186	3.182	3.318
N(20)-Cl(2)	3.167	3.191	3.187	3.191	3.196	3.231	3.188
N(30)-Cl(2)	3.255	3.255	3.252	3.248	3.246	3.198	3.249
N(30)-Cl(4)	3.298	3.3	3.291	3.299	3.311	3.485	3.306
**Ionic Contacts**							
N(20)-Cl(3)	3,408	3.366	3.379	3.368	3.360	3.352	3.353
N(20)-Cl(3)	4.440	4.441	4.426	4.419	4.411	4.391	3,896
N(20)-Cl(4)	3.940	3.932	3.922	3.915	3.909	3.806	3.505
N(30)-Cl(1)	3.598	3.568	3.566	3.547	3.531	3.433	3,505
N(30)-Cl(5)	3.542	3.526	3.517	3.507	3.497	3.493	3,471

Table S3. Cation-cation contacts in **Ln-6** structures.

C-C Contacts	La	Ce	Pr	Nd	Sm	Eu	Gd
C(45)---C(84)			3.492	3.475	3.469	3.46	3.458
C(22)---C(73)	3.663	3.660					
C(12)---C(25)			3.7	3.667	3.644	3.647	3.638
C(11)---C(24)	3.699	3.680					
C(14)----C(73)			3.676	3.638	3.652	3.656	3.668
C(32)---C(52)			3.787	3.77	3.804	3.791	3.79

Table S4. C-H•••Cl hydrogen bonds in the **Ln-5** structures.

C-H•••Cl	C•••Cl Distance (Å)	C-H•••Cl Angle (deg)
C(12)-H(12)•••Cl(5)	3.665	137.8
C(13)-H(13)•••Cl(2)	3.614	162.1
C(14)-H(14)•••Cl(3)	3.571	138.5
C(21)-H(21)•••Cl(4)	3.615	134.3
C(22)-H2(2)•••Cl(5)	3.749	172.4
C(24)-H(24)•••Cl(1)	3.593	126.7
C(25)-H(25)•••Cl(5)	3.607	121.6
C(32-H(32)•••Cl(3)	3.65	153.5
C(33)-H(33)•••Cl(1)	3.596	120.6
C(34)-H(34)•••Cl(3)	3.705	130.5
C(34)-H(34)•••Cl(4)	3.662	150.7

Table S5. C-H•••Cl Hydrogen bonds in the **Pr-6** structure

C-H•••Cl	C-H Distance (Å)	C-H-Cl Angle (deg)
C(11)-H(11)•••Cl(23)	3.666	135.8
C(11)-H(11)•••Cl(26)	3.783	145.5
C(12)-H(12)•••Cl(22)	3.497	149
C(13)-H(13)•••Cl(12)	3.773	143.3
C(13)-H(13)•••Cl(13)	3.724	134.5
C(14)-H(14)•••Cl(16)	3.593	137.4
C(21)-H(21)•••Cl(23)	3.834	136.9
C(22)-H(22)•••Cl(13)	3.571	129.9
C(24)-H(24)•••Cl(12)	3.588	137.5
C(32)-H(32)•••Cl(22)	3.51	136.9
C(33)-H(33)•••Cl(25)	3.731	135.3
C(34)-H(34)•••Cl(16)	3.492	150
C(35)-H(35)•••Cl(14)	3.633	133.2
C(41)-H(41)•••Cl(14)	3.787	125.7
C(42)-H(42)•••Cl(21)	3.572	134.5
C(44)-H(44)•••Cl(25)	3.612	129
C(45)-H(45)•••Cl(15)	3.722	136.1
C(51)-H(51)•••Cl(11)	3.558	134.3
C(52)-H(52)•••Cl(22)	3.516	135.5
C(54)-H(54)•••Cl(11)	3.65	136.8
C(54)-H(54)•••Cl(13)	3.424	135.8
C(55)-H(55)•••Cl(13)	3.652	120.2
C(64)-H(65)•••Cl(14)	3.716	159
C(71)-H(71)•••Cl(21)	3.482	126.8
C(72)-H(72)•••Cl(21)	3.548	123

C(72)-H(72)•••Cl(24)	3.659	151.7
C(74)-H(74)•••Cl(16)	3.536	121.5
C(75)-H(75)•••Cl(24)	3.416	137.3
C(81)-H(81)•••Cl(26)	3.582	157
C(85)-H(85)•••Cl(16)	3.652	136.5

Table S6. Coulumbic energy calculations for: **Nd-6**, **Gd-6**, **Tb-5**, and **Lu-5**.

**Structure**	**Energy (Kcal/mole)**	**Multiplier (num)(charge)**	**Total**
**Nd-6**			
Nd-Cl	-365	6	-2190
cis Cl-4 Cl	348	6	2088
trans Cl---Cl	61	6	366
5% covalent correction[13]			-123
H-bonds			-25
**Total (Kcal/mol)**			**+116**
**Gd-6**			
Gd-Cl	-371	6	-2230
cis Cl-4 Cl	349	6	2094
trans Cl---Cl	62	6	372
5% covalent correction[13]			-123
H-bonds			-25
**Total (Kcal/mol)**			**+88**
**Tb-5**			
Tb-Cl	-376	5	-1880
Cis Cl-4 Cl	355	1	355
Cis Cl-3Cl	266	4	1064
Trans Cl-Cl	63	4	252
5% covalent correction[13]			-74
**Total (Kcal/mol)**			**-283**
**Lu-5**			
Lu-Cl	-387	1	-1935
Cis Cl-4Cl	364	1	364
Cis Cl-3Cl	273	4	1092
Trans Cl-Cl	64	4	256
5% covalent correction[13]			-86
**Total (Kcal/mol)**			**-309**

Table S7. F-test results **for Ln-5** species.

Compound	R Pnma	R $Pna2_1$	F	Significance[a]
**Tb-5**	5.61	1.78	19	> 99%
**Dy-5**	5.76	1.37	51	> 99%
**Ho-5**	6.26	1.65	28	> 99%
**Er-5**	4.83	1.05	43	> 99%
**Tm-5**	5.45	2.27	10	> 99%
**Yb-5**	5.25	2.16	10	> 99%
**Lu-5**	4.67	1.9	11	> 99%

[a]An F-value of 1 is 99% significant

(L Downward, C H Booth, W. W Lukens, and F Bridges AIC Conference Proceedings,882 129 (2007))

checkCIF (basic structural check) running

Checking for embedded fcf data in CIF ...
Found embedded fcf data in CIF. Extracting fcf data from uploaded CIF, please wait . . . .

checkCIF/PLATON (basic structural check)

Structure factors have been supplied for datablock(s) tjbJL217_0m_a

THIS REPORT IS FOR GUIDANCE ONLY. IF USED AS PART OF A REVIEW PROCEDURE FOR PUBLICATION, IT SHOULD NOT REPLACE THE EXPERTISE OF AN EXPERIENCED CRYSTALLOGRAPHIC REFEREE.

No syntax errors found. CIF dictionary

Please wait while processing .... Interpreting this report

Structure factor report

Datablock: tjbJL217_0m_a

```
Bond precision:     C-C = 0.0031 A             Wavelength=0.71073
Cell:        a=9.7104(6)    b=15.6614(10)  c=18.1015(12)
             alpha=90       beta=90        gamma=90
Temperature:100 K
                  Calculated                     Reported
Volume            2752.9(3)                      2752.8(3)
Space group       P 21 21 21                     P 21 21 21
Hall group        P 2ac 2ab                      P 2ac 2ab
Moiety formula    Cl6 La, 3(C5 H6 N), C5 H5 N    ?
Sum formula       C20 H23 Cl6 La N4              C20 H23 Cl6 La N4
Mr                671.03                         671.03
Dx,g cm-3         1.619                          1.619
Z                 4                              4
Mu (mm-1)         2.149                          2.149
F000              1320.0                         1320.0
F000'             1322.64
h,k,lmax          18,29,34                       18,29,34
Nref              20004[ 10855]                  19429
Tmin,Tmax         0.445,0.828                    0.447,0.851
Tmin'             0.332
Correction   method=  #  Reported  T  Limits:  Tmin=0.447
Tmax=0.851 AbsCorr = MULTI-SCAN
```

Data completeness= 1.79/0.97 Theta(max)= 42.655

R(reflections)= 0.0227(18421) wR2(reflections)= 0.0517(19429)

S = 1.146 Npar= 281

The following ALERTS were generated. Each ALERT has the format

test-name_ALERT_alert-type_alert-level.

Click on the hyperlinks for more details of the test.

Alert level C

ABSTY02_ALERT_1_C An _exptl_absorpt_correction_type has been given without
a literature citation. This should be contained in the
_exptl_absorpt_process_details field.
Absorption correction given as multi-scan

STRVA01_ALERT_4_C Flack test results are ambiguous.
From the CIF: _refine_ls_abs_structure_Flack 0.500
From the CIF: _refine_ls_abs_structure_Flack_su 0.006

PLAT250_ALERT_2_C Large U3/U1 Ratio for Average U(i,j) Tensor .... 2.3 Note

PLAT911_ALERT_3_C Missing FCF Refl Between Thmin & STh/L= 0.600 69 Report

PLAT913_ALERT_3_C Missing # of Very Strong Reflections in FCF .... 40 Note

PLAT977_ALERT_2_C Check Negative Difference Density on H10 . -0.36 eA-3

Alert level G

PLAT007_ALERT_5_G Number of Unrefined Donor-H Atoms .... 3 Report

PLAT019_ALERT_1_G _diffrn_measured_fraction_theta_full/*_max < 1.0 0.998 Report

PLAT033_ALERT_4_G Flack x Value Deviates > 3.0 * sigma from Zero . 0.500 Note

PLAT232_ALERT_2_G Hirshfeld Test Diff (M-X) La1 --Cl12 . 12.8 s.u.

And 3 other PLAT232 Alerts

More ...

PLAT794_ALERT_5_G Tentative Bond Valency for La1 (III) . 3.05 Info

PLAT883_ALERT_1_G No Info/Value for _atom_sites_solution_primary . Please Do !

PLAT910_ALERT_3_G Missing # of FCF Reflection(s) Below Theta(Min). 3 Note

PLAT912_ALERT_4_G Missing # of FCF Reflections Above STh/L= 0.600 188 Note

PLAT978_ALERT_2_G Number C-C Bonds with Positive Residual Density. 2 Info

0 **ALERT level A** = Most likely a serious problem - resolve or explain

0 **ALERT level B** = A potentially serious problem, consider carefully

6 **ALERT level C** = Check. Ensure it is not caused by an omission or oversight

12 **ALERT level G** = General information/check it is not something unexpected

3 ALERT type 1 CIF construction/syntax error, inconsistent or missing data

7 ALERT type 2 Indicator that the structure model may be wrong or deficient

3 ALERT type 3 Indicator that the structure quality may be low

3 ALERT type 4 Improvement, methodology, query or suggestion

2 ALERT type 5 Informative message, check

It is advisable to attempt to resolve as many as possible of the alerts in all categories. Often the minor alerts point to easily fixed oversights, errors and omissions in your CIF or refinement strategy, so attention to these fine details can be worthwhile. In order to resolve some of the more serious problems it may be necessary to carry out additional measurements or structure refinements. However, the purpose of your study may justify the reported deviations and the more serious of these should normally be commented upon in the discussion or experimental section of a paper or in the "special_details" fields of the CIF. checkCIF was carefully designed to identify outliers and unusual parameters, but every test has its limitations and alerts that are not important in a particular case may appear. Conversely, the absence of alerts does not guarantee there are no aspects of the results needing attention. It is up to the individual to critically assess their own results and, if necessary, seek expert advice.

Publication of your CIF in IUCr journals

A basic structural check has been run on your CIF. These basic checks will be run on all CIFs submitted for publication in IUCr journals (*Acta Crystallographica*, *Journal of Applied Crystallography*, *Journal of Synchrotron Radiation*); however, if you intend to submit to *Acta Crystallographica Section C* or *E* or *IUCrData*, you should make sure that full publication checks are run on the final version of your CIF prior to submission.

Publication of your CIF in other journals

Please refer to the *Notes for Authors* of the relevant journal for any special instructions relating to CIF submission.

PLATON version of 12/09/2022; check.def file version of 09/08/2022

Datablock tjbJL217_0m_a - ellipsoid plot

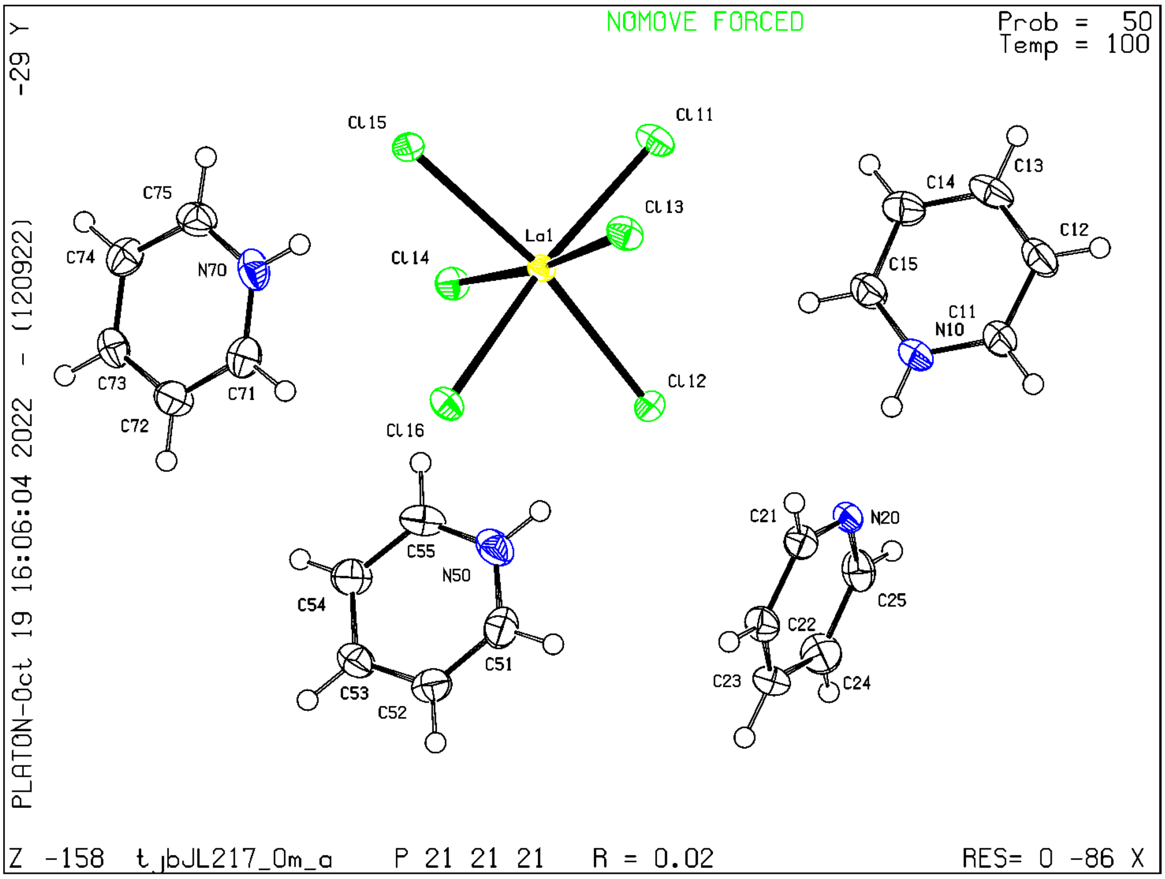

checkCIF (basic structural check) running

Checking for embedded fcf data in CIF ...
Found embedded fcf data in CIF. Extracting fcf data from uploaded CIF, please wait . . . . . .

checkCIF/PLATON (basic structural check)

Structure factors have been supplied for datablock(s) mo_jdbSP215_0m_a

THIS REPORT IS FOR GUIDANCE ONLY. IF USED AS PART OF A REVIEW PROCEDURE FOR PUBLICATION, IT SHOULD NOT REPLACE THE EXPERTISE OF AN EXPERIENCED CRYSTALLOGRAPHIC REFEREE.

No syntax errors found. CIF dictionary

Please wait while processing .... Interpreting this report

Datablock: mo_jdbSP215_0m_a

Bond precision: C-C = 0.0075 A Wavelength=0.71073

Cell: a=9.6382(3) b=15.6027(4) c=18.0596(6)
alpha=90 beta=90 gamma=90

Temperature: 100 K

	Calculated	Reported
Volume	2715.84(14)	2715.84(14)
Space group	P 21 21 21	P 21 21 21
Hall group	P 2ac 2ab	P 2ac 2ab
Moiety formula	Ce Cl6, 3(C5 H6 N), C5 H5 N	?
Sum formula	C20 H23 Ce Cl6 N4	C20 H23 Ce Cl6 N4
Mr	672.24	672.24
Dx,g cm-3	1.644	1.644
Z	4	4
Mu (mm-1)	2.282	2.282
F000	1324.0	1324.0
F000'	1326.79	
h,k,lmax	12,20,23	12,20,23
Nref	6265[3522]	6250
Tmin,Tmax	0.466,0.675	0.234,0.423
Tmin'	0.457	

Correction method= # Reported T Limits: Tmin=0.234 Tmax=0.423 AbsCorr = MULTI-SCAN

Data completeness= 1.77/1.00 Theta(max)= 27.517

R(reflections)= 0.0222(6245) wR2(reflections)= 0.0615(6250)

S = 1.088 Npar= 281

The following ALERTS were generated. Each ALERT has the format

test-name_ALERT_alert-type_alert-level.

Click on the hyperlinks for more details of the test.

Alert level B

PLAT094_ALERT_2_B Ratio of Maximum / Minimum Residual Density .... 4.99 Report

Alert level C

ABSTY02_ALERT_1_C An _exptl_absorpt_correction_type has been given without
a literature citation. This should be contained in the
_exptl_absorpt_process_details field.
Absorption correction given as multi-scan

STRVA01_ALERT_4_C Flack test results are ambiguous.

From the CIF: _refine_ls_abs_structure_Flack 0.503

From the CIF: _refine_ls_abs_structure_Flack_su 0.012

PLAT250_ALERT_2_C Large U3/U1 Ratio for Average U(i,j) Tensor .... 2.2 Note

PLAT911_ALERT_3_C Missing FCF Refl Between Thmin & STh/L= 0.600 5 Report

PLAT971_ALERT_2_C Check Calcd Resid. Dens. 0.63Ang From Ce1 2.34 eA-3

PLAT977_ALERT_2_C Check Negative Difference Density on H10 . -0.32 eA-3

Alert level G

PLAT007_ALERT_5_G Number of Unrefined Donor-H Atoms .... 3 Report

PLAT033_ALERT_4_G Flack x Value Deviates > 3.0 * sigma from Zero . 0.503 Note

PLAT115_ALERT_5_G ADDSYM Detects Noncrystallographic Inversion ... 83% Check

PLAT232_ALERT_2_G Hirshfeld Test Diff (M-X) Ce1 --Cl11 . 6.0 s.u.

And 5 other PLAT232 Alerts

More ...

PLAT794_ALERT_5_G Tentative Bond Valency for Ce1 (III) . 3.16 Info

PLAT883_ALERT_1_G No Info/Value for _atom_sites_solution_primary . Please Do !

PLAT910_ALERT_3_G Missing # of FCF Reflection(s) Below Theta(Min). 1 Note

PLAT912_ALERT_4_G Missing # of FCF Reflections Above STh/L= 0.600 4 Note

PLAT933_ALERT_2_G Number of HKL-OMIT Records in Embedded .res File 3 Note

PLAT978_ALERT_2_G Number C-C Bonds with Positive Residual Density. 4 Info

0 **ALERT level A** = Most likely a serious problem - resolve or explain

1 **ALERT level B** = A potentially serious problem, consider carefully

6 **ALERT level C** = Check. Ensure it is not caused by an omission or oversight

15 **ALERT level G** = General information/check it is not something unexpected

2 ALERT type 1 CIF construction/syntax error, inconsistent or missing data

12 ALERT type 2 Indicator that the structure model may be wrong or deficient

2 ALERT type 3 Indicator that the structure quality may be low

3 ALERT type 4 Improvement, methodology, query or suggestion

3 ALERT type 5 Informative message, check

It is advisable to attempt to resolve as many as possible of the alerts in all categories. Often the minor alerts point to easily fixed oversights, errors and omissions in your CIF or refinement strategy, so attention to these fine details can be worthwhile. In order to resolve some of the more serious problems it may be necessary to carry out additional measurements or structure refinements. However, the purpose of your study may justify the reported deviations and the more serious of these should normally be commented upon in the discussion or experimental section of a paper or in the "special_details" fields of the CIF. checkCIF was carefully designed to identify outliers and unusual parameters, but every test has its limitations and alerts that are not important in a particular case may appear. Conversely, the absence of alerts does not guarantee there are no aspects of the results needing attention. It is up to the individual to critically assess their own results and, if necessary, seek expert advice.

Publication of your CIF in IUCr journals

A basic structural check has been run on your CIF. These basic checks will be run on all CIFs submitted for publication in IUCr journals (*Acta Crystallographica*, *Journal of Applied Crystallography*, *Journal of Synchrotron Radiation*); however, if you intend to submit to *Acta Crystallographica Section C* or *E* or *IUCrData*, you should make sure that full publication checks are run on the final version of your CIF prior to submission.

Publication of your CIF in other journals

Please refer to the *Notes for Authors* of the relevant journal for any special instructions relating to CIF submission.

PLATON version of 12/09/2022; check.def file version of 09/08/2022

Datablock mo_jdbSP215_0m_a - ellipsoid plot

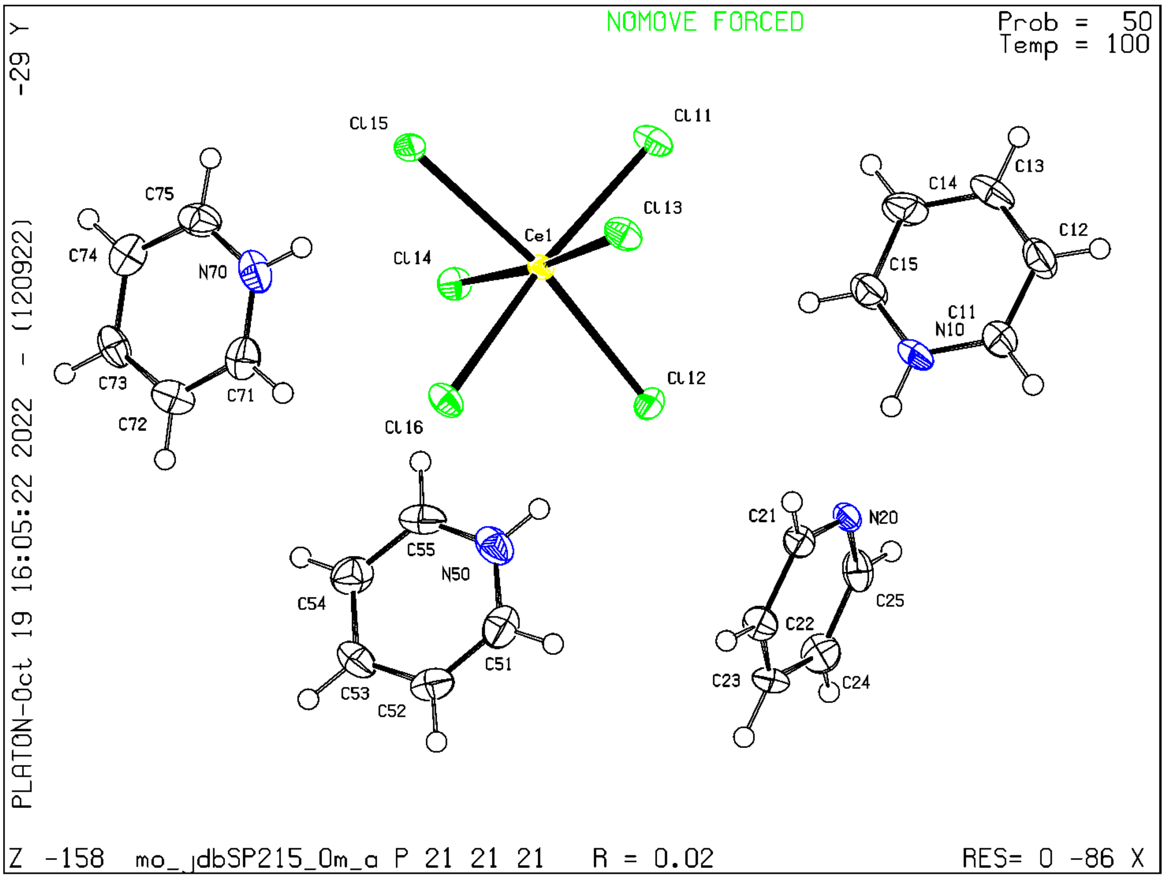

checkCIF (basic structural check) running

Checking for embedded fcf data in CIF ...

Found embedded fcf data in CIF. Extracting fcf data from uploaded CIF, please wait . . . .

checkCIF/PLATON (basic structural check)

Structure factors have been supplied for datablock(s) PrCl6

THIS REPORT IS FOR GUIDANCE ONLY. IF USED AS PART OF A REVIEW PROCEDURE FOR PUBLICATION, IT SHOULD NOT REPLACE THE EXPERTISE OF AN EXPERIENCED CRYSTALLOGRAPHIC REFEREE.

No syntax errors found. CIF dictionary

Please wait while processing .... Interpreting this report

Structure factor report

Datablock: PrCl6

Bond precision:	C-C = 0.0178 A		Wavelength=0.71073
Cell:	a=15.6181(11)	b=9.5868(8)	c=36.244(3)
	alpha=90	beta=89.999(3)	gamma=90
Temperature:	100 K		

	Calculated	Reported
Volume	5426.7(7)	5426.8(7)
Space group	P 21/c	P 21/c
Hall group	-P 2ybc	-P 2ybc
Moiety formula	Cl6 Pr, 3(C5 H6 N), C5 H5 N	?
Sum formula	C20 H23 Cl6 N4 Pr	C20 H23 Cl6 N4 Pr
Mr	673.03	673.03
Dx,g cm-3	1.648	1.648
Z	8	8
Mu (mm-1)	2.402	2.402
F000	2656.0	2656.0
F000'	2661.85	
h,k,lmax	24,14,55	23,14,55
Nref	20599	20468
Tmin,Tmax	0.563,0.725	0.793,0.892
Tmin'	0.371	

Correction method= # Reported T Limits: Tmin=0.793 Tmax=0.892 AbsCorr = MULTI-SCAN

Data completeness= 0.994 Theta(max)= 33.099

R(reflections)= 0.0555(14618) wR2(reflections)= 0.1271(20468)

S = 1.080 Npar= 560

The following ALERTS were generated. Each ALERT has the format

test-name_ALERT_alert-type_alert-level.

Click on the hyperlinks for more details of the test.

Alert level B

PLAT241_ALERT_2_B High 'MainMol' Ueq as Compared to Neighbors of C53 Check

Alert level C

ABSTY02_ALERT_1_C An _exptl_absorpt_correction_type has been given without
a literature citation. This should be contained in the
_exptl_absorpt_process_details field.
Absorption correction given as multi-scan
PLAT112_ALERT_2_C ADDSYM Detects New (Pseudo) Symm. Elem c/2 91 %Fit
PLAT157_ALERT_4_C Non-standard Monoclinic Beta Angle less 90 Deg 90.00 Degree
PLAT241_ALERT_2_C High 'MainMol' Ueq as Compared to Neighbors of C51 Check
PLAT242_ALERT_2_C Low 'MainMol' Ueq as Compared to Neighbors of Pr1 Check
And 3 other PLAT242 Alerts
More ...
PLAT250_ALERT_2_C Large U3/U1 Ratio for Average U(i,j) Tensor .... 3.0 Note
And 2 other PLAT250 Alerts
More ...
PLAT342_ALERT_3_C Low Bond Precision on C-C Bonds .... 0.01784 Ang.
PLAT905_ALERT_3_C Negative K value in the Analysis of Variance ... -0.221 Report
PLAT918_ALERT_3_C Reflection(s) with I(obs) much Smaller I(calc) . 1 Check

Alert level G

PLAT007_ALERT_5_G Number of Unrefined Donor-H Atoms .... 6 Report
PLAT083_ALERT_2_G SHELXL Second Parameter in WGHT Unusually Large 40.51 Why ?
PLAT232_ALERT_2_G Hirshfeld Test Diff (M-X) Pr1 --Cl14 . 5.6 s.u.
PLAT794_ALERT_5_G Tentative Bond Valency for Pr1 (III) . 3.22 Info
PLAT794_ALERT_5_G Tentative Bond Valency for Pr2 (III) . 3.12 Info
PLAT870_ALERT_4_G ALERTS Related to Twinning Effects Suppressed .. ! Info
PLAT883_ALERT_1_G No Info/Value for _atom_sites_solution_primary . Please Do !
PLAT910_ALERT_3_G Missing # of FCF Reflection(s) Below Theta(Min). 4 Note
PLAT912_ALERT_4_G Missing # of FCF Reflections Above STh/L= 0.600 127 Note
PLAT913_ALERT_3_G Missing # of Very Strong Reflections in FCF .... 1 Note
PLAT931_ALERT_5_G CIFcalcFCF Twin Law (0 0 1) Est.d BASF 0.48 Check
PLAT965_ALERT_2_G The SHELXL WEIGHT Optimisation has not Converged Please Check

0 **ALERT level A** = Most likely a serious problem - resolve or explain
1 **ALERT level B** = A potentially serious problem, consider carefully
14 **ALERT level C** = Check. Ensure it is not caused by an omission or oversight
12 **ALERT level G** = General information/check it is not something unexpected

2 ALERT type 1 CIF construction/syntax error, inconsistent or missing data

13 ALERT type 2 Indicator that the structure model may be wrong or deficient
5 ALERT type 3 Indicator that the structure quality may be low
3 ALERT type 4 Improvement, methodology, query or suggestion
4 ALERT type 5 Informative message, check

It is advisable to attempt to resolve as many as possible of the alerts in all categories. Often the minor alerts point to easily fixed oversights, errors and omissions in your CIF or refinement strategy, so attention to these fine details can be worthwhile. In order to resolve some of the more serious problems it may be necessary to carry out additional measurements or structure refinements. However, the purpose of your study may justify the reported deviations and the more serious of these should normally be commented upon in the discussion or experimental section of a paper or in the "special_details" fields of the CIF. checkCIF was carefully designed to identify outliers and unusual parameters, but every test has its limitations and alerts that are not important in a particular case may appear. Conversely, the absence of alerts does not guarantee there are no aspects of the results needing attention. It is up to the individual to critically assess their own results and, if necessary, seek expert advice.

Publication of your CIF in IUCr journals

A basic structural check has been run on your CIF. These basic checks will be run on all CIFs submitted for publication in IUCr journals (*Acta Crystallographica*, *Journal of Applied Crystallography*, *Journal of Synchrotron Radiation*); however, if you intend to submit to *Acta Crystallographica Section C* or *E* or *IUCrData*, you should make sure that full publication checks are run on the final version of your CIF prior to submission.

Publication of your CIF in other journals

Please refer to the *Notes for Authors* of the relevant journal for any special instructions relating to CIF submission.

PLATON version of 12/09/2022; check.def file version of 09/08/2022

Datablock PrCl6 - ellipsoid plot

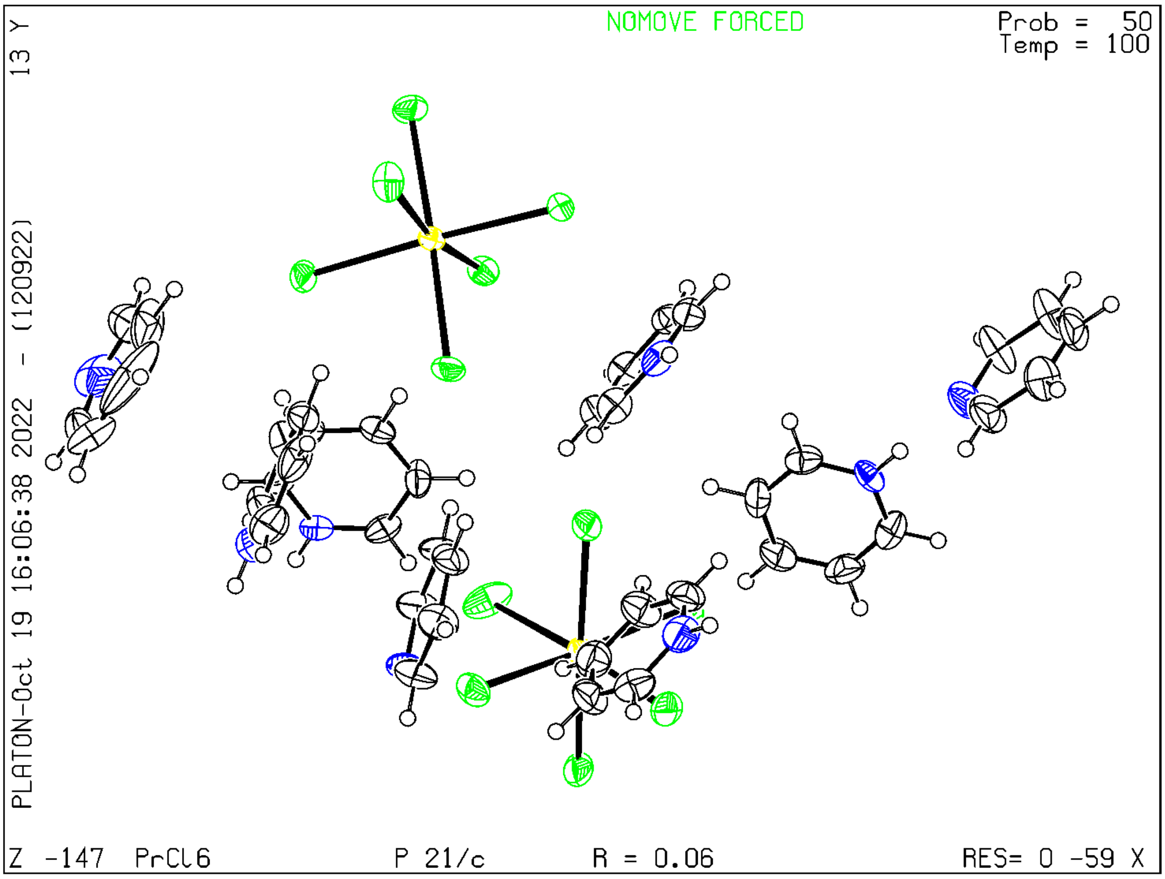

checkCIF (basic structural check) running

Checking for embedded fcf data in CIF ...
Found embedded fcf data in CIF. Extracting fcf data from uploaded CIF, please wait . . . .

checkCIF/PLATON (basic structural check)

Structure factors have been supplied for datablock(s) tjbAP226_0m_a

THIS REPORT IS FOR GUIDANCE ONLY. IF USED AS PART OF A REVIEW PROCEDURE FOR PUBLICATION, IT SHOULD NOT REPLACE THE EXPERTISE OF AN EXPERIENCED CRYSTALLOGRAPHIC REFEREE.

No syntax errors found. CIF dictionary

Please wait while processing .... Interpreting this report

Datablock: tjbAP226_0m_a

Bond precision:	C-C = 0.0204 A		Wavelength=0.71073
Cell:	a=15.561(2)	b=9.5667(15)	c=36.204(5)
	alpha=90	beta=90.306(5)	gamma=90
Temperature:	100 K		

	Calculated	Reported
Volume	5389.5(13)	5389.3(14)
Space group	P 21/c	P 21/c
Hall group	-P 2ybc	-P 2ybc
Moiety formula	Cl6 Nd, 3(C5 H6 N), C5 H5 N	?
Sum formula	C20 H23 Cl6 N4 Nd	C20 H23 Cl6 N4 Nd
Mr	676.36	676.36
Dx,g cm-3	1.667	1.667
Z	8	8
Mu (mm-1)	2.537	2.537
F000	2664.0	2664.0
F000'	2670.04	
h,k,lmax	24,15,57	24,15,57
Nref	22574	19339
Tmin,Tmax	0.636,0.728	0.681,0.746
Tmin'	0.428	

Correction method= # Reported T Limits: Tmin=0.681 Tmax=0.746 AbsCorr = ?

Data completeness= 0.857 Theta(max)= 34.344

R(reflections)= 0.0814(14271) wR2(reflections)= 0.1959(19339)

S = 1.191 Npar= 561

The following ALERTS were generated. Each ALERT has the format

test-name_ALERT_alert-type_alert-level.

Click on the hyperlinks for more details of the test.

Alert level B

PLAT342_ALERT_3_B Low Bond Precision on C-C Bonds .... 0.02037 Ang.

Alert level C

PLAT052_ALERT_1_C Info on Absorption Correction Method Not Given Please Do !
PLAT057_ALERT_3_C Correction for Absorption Required RT(exp) ... 1.15 Do !
PLAT241_ALERT_2_C High 'MainMol' Ueq as Compared to Neighbors of C53 Check
PLAT242_ALERT_2_C Low 'MainMol' Ueq as Compared to Neighbors of C55 Check

PLAT250_ALERT_2_C Large U3/U1 Ratio for Average U(i,j) Tensor .... 2.4 Note

And 2 other PLAT250 Alerts

More ...

PLAT906_ALERT_3_C Large K Value in the Analysis of Variance ...... 4.369 Check

PLAT911_ALERT_3_C Missing FCF Refl Between Thmin & STh/L= 0.600 238 Report

Alert level G

PLAT007_ALERT_5_G Number of Unrefined Donor-H Atoms .... 6 Report

PLAT083_ALERT_2_G SHELXL Second Parameter in WGHT Unusually Large 80.17 Why ?

PLAT794_ALERT_5_G Tentative Bond Valency for Nd1 (III) . 3.23 Info

PLAT794_ALERT_5_G Tentative Bond Valency for Nd2 (III) . 3.19 Info

PLAT870_ALERT_4_G ALERTS Related to Twinning Effects Suppressed .. ! Info

PLAT883_ALERT_1_G No Info/Value for _atom_sites_solution_primary . Please Do !

PLAT910_ALERT_3_G Missing # of FCF Reflection(s) Below Theta(Min). 4 Note

PLAT912_ALERT_4_G Missing # of FCF Reflections Above STh/L= 0.600 2993 Note

PLAT913_ALERT_3_G Missing # of Very Strong Reflections in FCF .... 2 Note

PLAT931_ALERT_5_G CIFcalcFCF Twin Law [0 0 1] Est.d BASF 0.30 Check

PLAT965_ALERT_2_G The SHELXL WEIGHT Optimisation has not Converged Please Check

0 **ALERT level A** = Most likely a serious problem - resolve or explain

1 **ALERT level B** = A potentially serious problem, consider carefully

9 **ALERT level C** = Check. Ensure it is not caused by an omission or oversight

11 **ALERT level G** = General information/check it is not something unexpected

2 ALERT type 1 CIF construction/syntax error, inconsistent or missing data

7 ALERT type 2 Indicator that the structure model may be wrong or deficient

6 ALERT type 3 Indicator that the structure quality may be low

2 ALERT type 4 Improvement, methodology, query or suggestion

4 ALERT type 5 Informative message, check

It is advisable to attempt to resolve as many as possible of the alerts in all categories. Often the minor alerts point to easily fixed oversights, errors and omissions in your CIF or refinement strategy, so attention to these fine details can be worthwhile. In order to resolve some of the more serious problems it may be necessary to carry out additional measurements or structure refinements. However, the purpose of your study may justify the reported deviations and the more serious of these should normally be commented upon in the discussion or experimental section of a paper or in the "special_details" fields of the CIF. checkCIF was carefully designed to identify outliers and unusual parameters, but every test has its limitations and alerts that are not important in a particular case may appear. Conversely, the absence of alerts does not guarantee there are no aspects of the results needing attention. It is up to the individual to critically assess their own results and, if necessary, seek expert advice.

Publication of your CIF in IUCr journals

A basic structural check has been run on your CIF. These basic checks will be run on all CIFs submitted for publication in IUCr journals (*Acta Crystallographica*, *Journal of Applied Crystallography*, *Journal of Synchrotron Radiation*); however, if you intend to submit to *Acta Crystallographica Section C* or *E* or *IUCrData*, you should make sure that full publication checks are run on the final version of your CIF prior to submission.

Publication of your CIF in other journals
Please refer to the *Notes for Authors* of the relevant journal for any special instructions relating to CIF submission.

PLATON version of 12/09/2022; check.def file version of 09/08/2022

Datablock tjbAP226_0m_a - ellipsoid plot

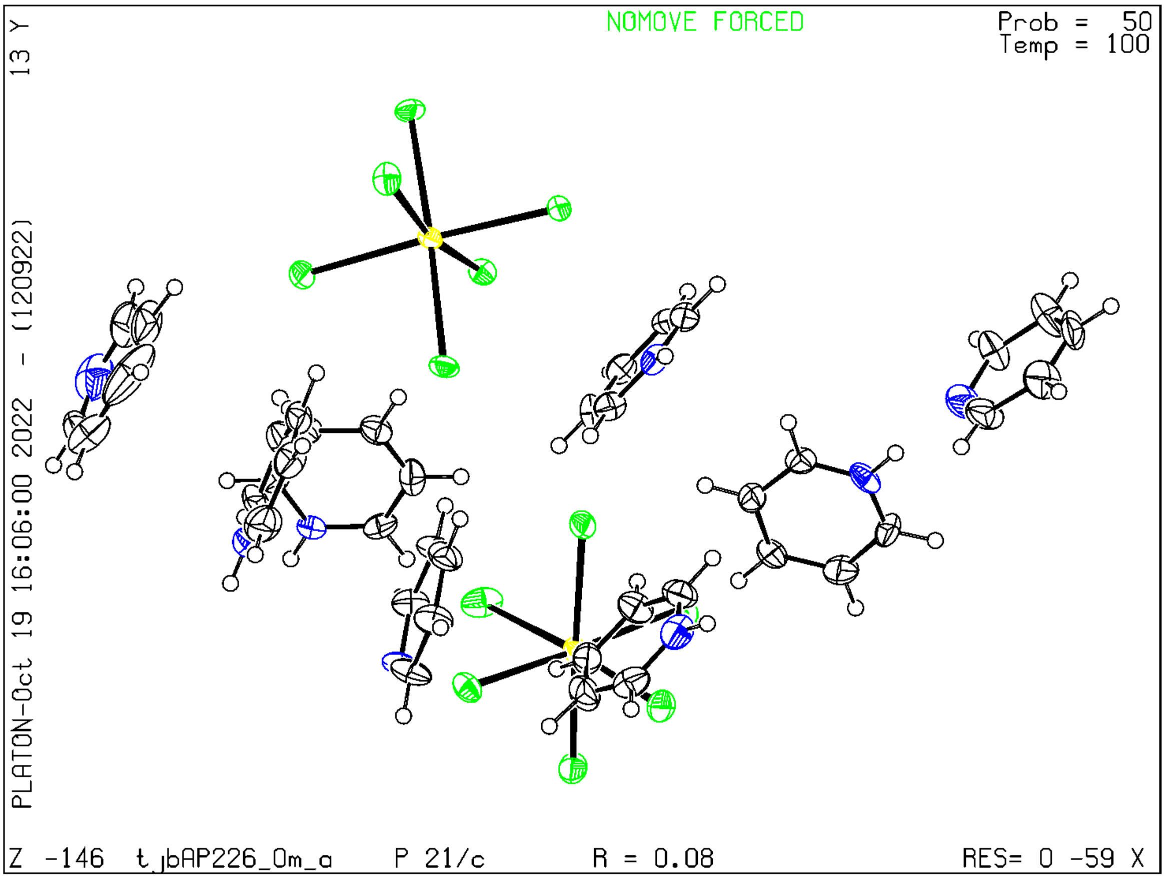

checkCIF (basic structural check) running

Checking for embedded fcf data in CIF ...
Found embedded fcf data in CIF. Extracting fcf data from uploaded CIF, please wait . . . .

checkCIF/PLATON (basic structural check)

Structure factors have been supplied for datablock(s) tjbap224_0m_a_sx

THIS REPORT IS FOR GUIDANCE ONLY. IF USED AS PART OF A REVIEW PROCEDURE FOR PUBLICATION, IT SHOULD NOT REPLACE THE EXPERTISE OF AN EXPERIENCED CRYSTALLOGRAPHIC REFEREE.

No syntax errors found. CIF dictionary

Please wait while processing .... Interpreting this report

Structure factor report

Datablock: tjbap224_0m_a_sx

Bond precision:	C-C = 0.0081 A		Wavelength=0.71073
Cell:	a=15.5230(14)	b=9.5386(9)	c=36.265(4)
	alpha=90	beta=90.244(4)	gamma=90
Temperature:	100 K		

	Calculated	Reported
Volume	5369.6(9)	5369.6(9)
Space group	P 21/c	P 21/c
Hall group	-P 2ybc	-P 2ybc
Moiety formula	Cl6 Sm, 3(C5 H6 N), C5 H5 N	?
Sum formula	C20 H23 Cl6 N4 Sm	C20 H23 Cl6 N4 Sm
Mr	682.48	682.47
Dx,g cm-3	1.689	1.688
Z	8	8
Mu (mm-1)	2.800	2.800
F000	2680.0	2680.0
F000'	2686.29	
h,k,lmax	25,15,59	25,15,59
Nref	24794	22665
Tmin,Tmax	0.460,0.683	0.208,0.327
Tmin'	0.255	

Correction method= # Reported T Limits: Tmin=0.208 Tmax=0.327 AbsCorr = MULTI-SCAN

Data completeness= 0.914	Theta(max)= 35.633
R(reflections)= 0.0445(20093)	wR2(reflections)= 0.1078(22665)
S = 1.179	Npar= 560

The following ALERTS were generated. Each ALERT has the format

test-name_ALERT_alert-type_alert-level.

Click on the hyperlinks for more details of the test.

Alert level C

ABSTY02_ALERT_1_C An _exptl_absorpt_correction_type has been given without a literature citation. This should be contained in the

_exptl_absorpt_process_details field.

Absorption correction given as multi-scan

PLAT250_ALERT_2_C Large U3/U1 Ratio for Average U(i,j) Tensor .... 2.7 Note

PLAT250_ALERT_2_C Large U3/U1 Ratio for Average U(i,j) Tensor .... 2.6 Note

PLAT342_ALERT_3_C Low Bond Precision on C-C Bonds .... 0.00813 Ang.

PLAT911_ALERT_3_C Missing FCF Refl Between Thmin & STh/L= 0.600 21 Report

Alert level G

PLAT007_ALERT_5_G Number of Unrefined Donor-H Atoms .... 6 Report

PLAT083_ALERT_2_G SHELXL Second Parameter in WGHT Unusually Large 26.11 Why ?

PLAT870_ALERT_4_G ALERTS Related to Twinning Effects Suppressed .. ! Info

PLAT883_ALERT_1_G No Info/Value for _atom_sites_solution_primary . Please Do !

PLAT910_ALERT_3_G Missing # of FCF Reflection(s) Below Theta(Min). 4 Note

PLAT912_ALERT_4_G Missing # of FCF Reflections Above STh/L= 0.600 2104 Note

PLAT913_ALERT_3_G Missing # of Very Strong Reflections in FCF .... 2 Note

PLAT931_ALERT_5_G CIFcalcFCF Twin Law [0 0 1] Est.d BASF 0.26 Check

PLAT965_ALERT_2_G The SHELXL WEIGHT Optimisation has not Converged Please Check

0 **ALERT level A** = Most likely a serious problem - resolve or explain

0 **ALERT level B** = A potentially serious problem, consider carefully

5 **ALERT level C** = Check. Ensure it is not caused by an omission or oversight

9 **ALERT level G** = General information/check it is not something unexpected

2 ALERT type 1 CIF construction/syntax error, inconsistent or missing data

4 ALERT type 2 Indicator that the structure model may be wrong or deficient

4 ALERT type 3 Indicator that the structure quality may be low

2 ALERT type 4 Improvement, methodology, query or suggestion

2 ALERT type 5 Informative message, check

It is advisable to attempt to resolve as many as possible of the alerts in all categories. Often the minor alerts point to easily fixed oversights, errors and omissions in your CIF or refinement strategy, so attention to these fine details can be worthwhile. In order to resolve some of the more serious problems it may be necessary to carry out additional measurements or structure refinements. However, the purpose of your study may justify the reported deviations and the more serious of these should normally be commented upon in the discussion or experimental section of a paper or in the "special_details" fields of the CIF. checkCIF was carefully designed to identify outliers and unusual parameters, but every test has its limitations and alerts that are not important in a particular case may appear. Conversely, the absence of alerts does not guarantee there are no aspects of the results needing attention. It is up to the individual to critically assess their own results and, if necessary, seek expert advice.

Publication of your CIF in IUCr journals

A basic structural check has been run on your CIF. These basic checks will be run on all CIFs submitted for publication in IUCr journals (*Acta Crystallographica*, *Journal of Applied Crystallography*, *Journal of Synchrotron Radiation*); however, if you intend to submit to *Acta Crystallographica Section C* or *E* or *IUCrData*, you should make sure that full publication checks are run on the final version of your CIF prior to submission.

Publication of your CIF in other journals

Please refer to the *Notes for Authors* of the relevant journal for any special instructions relating to CIF submission.

PLATON version of 12/09/2022; check.def file version of 09/08/2022

Datablock tjbap224_0m_a_sx - ellipsoid plot

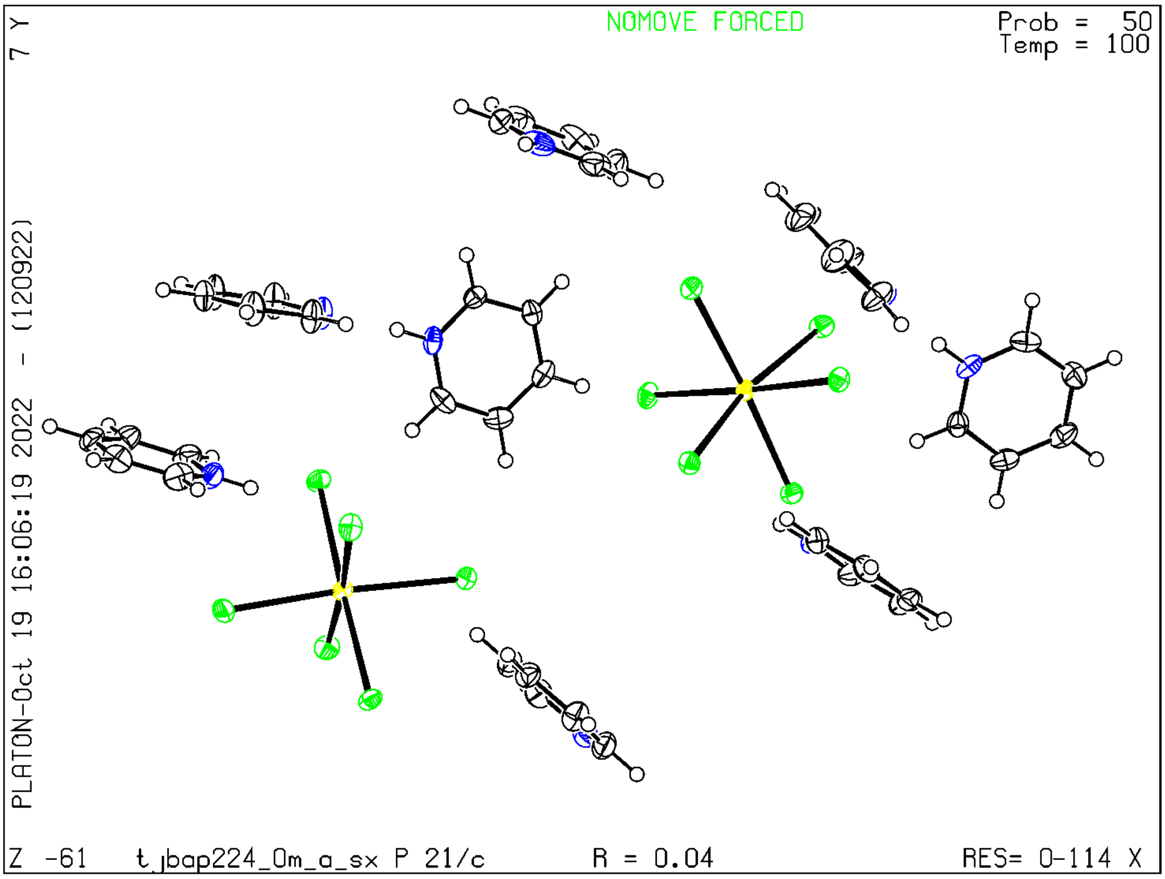

checkCIF (basic structural check) running

Checking for embedded fcf data in CIF ...
Found embedded fcf data in CIF. Extracting fcf data from uploaded CIF, please wait . . . .

checkCIF/PLATON (basic structural check)

Structure factors have been supplied for datablock(s) P21SEE

THIS REPORT IS FOR GUIDANCE ONLY. IF USED AS PART OF A REVIEW PROCEDURE FOR PUBLICATION, IT SHOULD NOT REPLACE THE EXPERTISE OF AN EXPERIENCED CRYSTALLOGRAPHIC REFEREE.

No syntax errors found. CIF dictionary

Please wait while processing .... Interpreting this report

Structure factor report

Datablock: P21SEE

```
Bond precision:     C-C = 0.0102 A              Wavelength=0.71073
Cell:          a=15.534(3)    b=9.5290(19)   c=36.287(7)
               alpha=90       beta=90.091(7) gamma=90
Temperature:100 K
                    Calculated                     Reported
Volume              5371.3(18)                     5371.5(18)
Space group         P 21/c                         P 21/c
Hall group          -P 2ybc                        -P 2ybc
Moiety formula      Cl6 Eu, 3(C5 H6 N), C5 H5 N    ?
Sum formula         C20 H23 Cl6 Eu N4              C20 H23 Cl6 Eu N4
Mr                  684.09                         684.08
Dx,g cm-3           1.692                          1.692
Z                   8                              8
Mu (mm-1)           2.948                          2.948
F000                2688.0                         2688.0
F000'               2694.35
h,k,lmax            28,17,66                       23,15,65
Nref                33963                          23961
Tmin,Tmax           0.451,0.564                    0.262,0.627
Tmin'               0.129
Correction  method=  #  Reported  T  Limits:  Tmin=0.262
Tmax=0.627 AbsCorr = MULTI-SCAN
Data completeness= 0.706       Theta(max)= 40.319
R(reflections)= 0.0481( 18463)           wR2(reflections)=
                                         0.1511( 23961)
S = 1.101              Npar= 561
```

The following ALERTS were generated. Each ALERT has the format

test-name_ALERT_alert-type_alert-level.

Click on the hyperlinks for more details of the test.

Alert level C

ABSTY02_ALERT_1_C An _exptl_absorpt_correction_type has been given without a literature citation. This should be contained in the _exptl_absorpt_process_details field.

Absorption correction given as multi-scan

DIFMX02_ALERT_1_C The maximum difference density is > 0.1*ZMAX*0.75
The relevant atom site should be identified.

PLAT097_ALERT_2_C Large Reported Max. (Positive) Residual Density 5.39 eA-3

PLAT250_ALERT_2_C Large U3/U1 Ratio for Average U(i,j) Tensor .... 2.3 Note

PLAT250_ALERT_2_C Large U3/U1 Ratio for Average U(i,j) Tensor .... 2.2 Note

PLAT342_ALERT_3_C Low Bond Precision on C-C Bonds .... 0.01025 Ang.

PLAT905_ALERT_3_C Negative K value in the Analysis of Variance ... -0.457 Report

PLAT911_ALERT_3_C Missing FCF Refl Between Thmin & STh/L= 0.600 16 Report

Alert level G

PLAT007_ALERT_5_G Number of Unrefined Donor-H Atoms .... 6 Report

PLAT063_ALERT_4_G Crystal Size Possibly too Large for Beam Size .. 0.69 mm

PLAT083_ALERT_2_G SHELXL Second Parameter in WGHT Unusually Large 29.43 Why ?

PLAT794_ALERT_5_G Tentative Bond Valency for Eu1 (III) . 3.43 Info

PLAT794_ALERT_5_G Tentative Bond Valency for Eu2 (III) . 3.41 Info

PLAT870_ALERT_4_G ALERTS Related to Twinning Effects Suppressed .. ! Info

PLAT883_ALERT_1_G No Info/Value for _atom_sites_solution_primary . Please Do !

PLAT910_ALERT_3_G Missing # of FCF Reflection(s) Below Theta(Min). 4 Note

PLAT912_ALERT_4_G Missing # of FCF Reflections Above STh/L= 0.600 8050 Note

PLAT913_ALERT_3_G Missing # of Very Strong Reflections in FCF .... 2 Note

PLAT931_ALERT_5_G CIFcalcFCF Twin Law (0 0 1) Est.d BASF 0.37 Check

PLAT950_ALERT_5_G Calculated (ThMax) and CIF-Reported Hmax Differ 5 Units

PLAT951_ALERT_5_G Calculated (ThMax) and CIF-Reported Kmax Differ 2 Units

PLAT956_ALERT_1_G Calculated (ThMax) and Actual (FCF) Hmax Differ 5 Units

PLAT957_ALERT_1_G Calculated (ThMax) and Actual (FCF) Kmax Differ 2 Units

PLAT965_ALERT_2_G The SHELXL WEIGHT Optimisation has not Converged Please Check

0 **ALERT level A** = Most likely a serious problem - resolve or explain
0 **ALERT level B** = A potentially serious problem, consider carefully
8 **ALERT level C** = Check. Ensure it is not caused by an omission or oversight
16 **ALERT level G** = General information/check it is not something unexpected

5 ALERT type 1 CIF construction/syntax error, inconsistent or missing data
5 ALERT type 2 Indicator that the structure model may be wrong or deficient
5 ALERT type 3 Indicator that the structure quality may be low
3 ALERT type 4 Improvement, methodology, query or suggestion
6 ALERT type 5 Informative message, check

It is advisable to attempt to resolve as many as possible of the alerts in all categories. Often the minor alerts point to easily fixed oversights, errors and omissions in your CIF or refinement strategy, so attention to these fine details can be worthwhile. In order to resolve some of the more serious problems it may be necessary to

carry out additional measurements or structure refinements. However, the purpose of your study may justify the reported deviations and the more serious of these should normally be commented upon in the discussion or experimental section of a paper or in the "special_details" fields of the CIF. checkCIF was carefully designed to identify outliers and unusual parameters, but every test has its limitations and alerts that are not important in a particular case may appear. Conversely, the absence of alerts does not guarantee there are no aspects of the results needing attention. It is up to the individual to critically assess their own results and, if necessary, seek expert advice.

Publication of your CIF in IUCr journals

A basic structural check has been run on your CIF. These basic checks will be run on all CIFs submitted for publication in IUCr journals (*Acta Crystallographica*, *Journal of Applied Crystallography*, *Journal of Synchrotron Radiation*); however, if you intend to submit to *Acta Crystallographica Section C* or *E* or *IUCrData*, you should make sure that full publication checks are run on the final version of your CIF prior to submission.

Publication of your CIF in other journals

Please refer to the *Notes for Authors* of the relevant journal for any special instructions relating to CIF submission.

PLATON version of 12/09/2022; check.def file version of 09/08/2022

Datablock P21SEE - ellipsoid plot

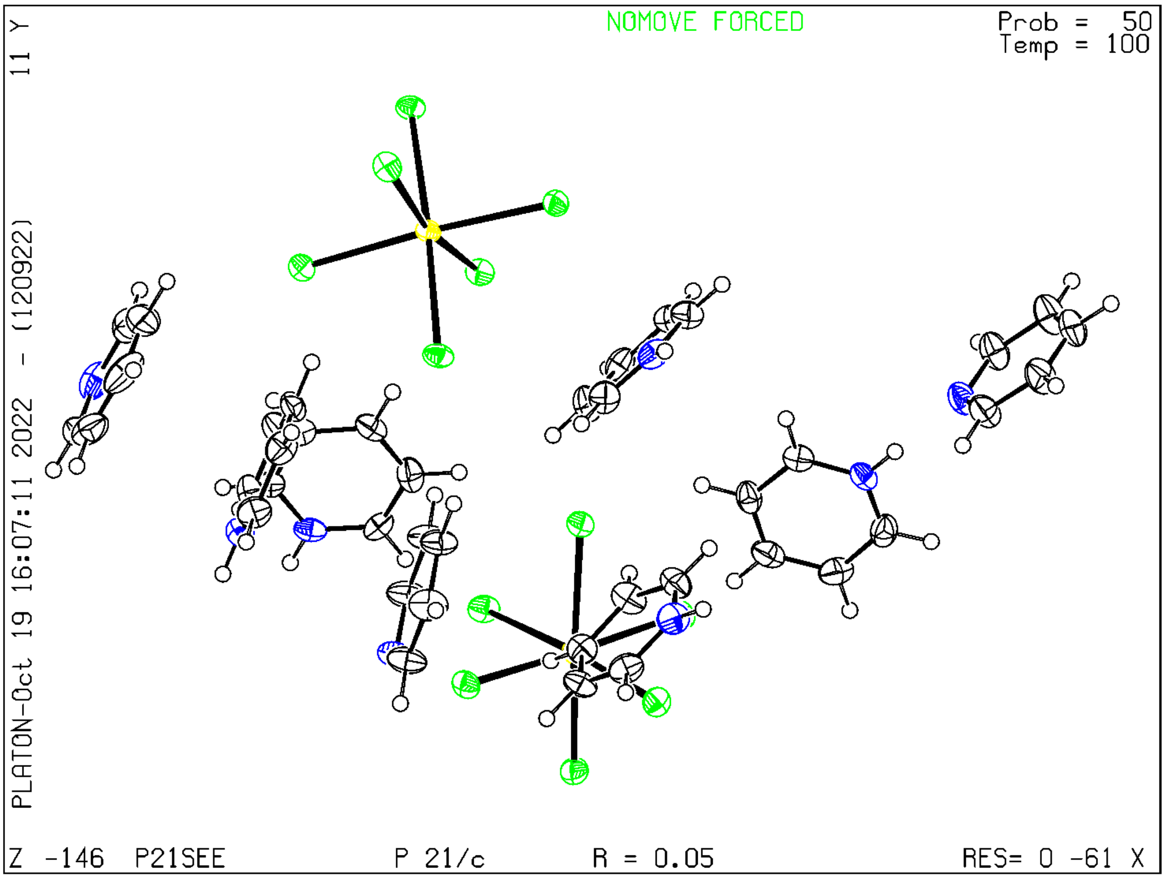

checkCIF (basic structural check) running

Checking for embedded fcf data in CIF ...
Found embedded fcf data in CIF. Extracting fcf data from uploaded CIF, please wait . . . .

checkCIF/PLATON (basic structural check)

Structure factors have been supplied for datablock(s) GdCl3Py4_a

THIS REPORT IS FOR GUIDANCE ONLY. IF USED AS PART OF A REVIEW PROCEDURE FOR PUBLICATION, IT SHOULD NOT REPLACE THE EXPERTISE OF AN EXPERIENCED CRYSTALLOGRAPHIC REFEREE.

No syntax errors found. CIF dictionary

Please wait while processing .... Interpreting this report

Structure factor report

Datablock: GdCl3Py4_a

```
Bond precision:     C-C = 0.0036 A              Wavelength=0.71073
Cell:          a=9.7480(15)    b=16.884(2)     c=15.765(2)
               alpha=90        beta=103.813(5) gamma=90
Temperature:100 K
```

	Calculated	Reported
Volume	2519.7(6)	2519.6(6)
Space group	P 21/n	P 21/n
Hall group	-P 2yn	-P 2yn
Moiety formula	2(C20 H20 Cl3 Gd N4), C5 H5 N	?
Sum formula	C45 H45 Cl6 Gd2 N9	C22.50 H22.50 Cl3 Gd N4.50
Mr	1239.10	619.55
Dx,g cm-3	1.633	1.633
Z	2	4
Mu (mm-1)	2.968	2.968
F000	1216.0	1216.0
F000'	1217.36	
h,k,lmax	13,23,21	13,23,21
Nref	6834	6812
Tmin,Tmax	0.355,0.761	0.141,0.346
Tmin'	0.146	

```
Correction method= # Reported T Limits: Tmin=0.141
Tmax=0.346 AbsCorr = MULTI-SCAN
Data completeness= 0.997       Theta(max)= 29.192
R(reflections)= 0.0204( 6298)          wR2(reflections)=
                                       0.0470( 6812)
S = 1.229              Npar= 295
```

The following ALERTS were generated. Each ALERT has the format

test-name_ALERT_alert-type_alert-level.

Click on the hyperlinks for more details of the test.

Alert level A

PLAT430_ALERT_2_A Short Inter D...A Contact N1 ..N1 . 2.49 Ang.
1-x,2-y,-z = 3_675 Check

Author Response: "The noted distance is due to a pyridine solvate that sits on an inver

Alert level B

PLAT934_ALERT_3_B Number of (Iobs-Icalc)/Sigma(W) > 10 Outliers .. 2 Check

Alert level C

ABSTY02_ALERT_1_C An _exptl_absorpt_correction_type has been given without
a literature citation. This should be contained in the
_exptl_absorpt_process_details field.
Absorption correction given as multi-scan

PLAT977_ALERT_2_C Check Negative Difference Density on H35 . -0.35 eA-3

Alert level G

PLAT003_ALERT_2_G Number of Uiso or Uij Restrained non-H Atoms ... 1 Report

PLAT045_ALERT_1_G Calculated and Reported Z Differ by a Factor ... 0.500 Check

PLAT063_ALERT_4_G Crystal Size Possibly too Large for Beam Size .. 0.63 mm

PLAT186_ALERT_4_G The CIF-Embedded .res File Contains ISOR Records 1 Report

PLAT232_ALERT_2_G Hirshfeld Test Diff (M-X) Gd1 --Cl1 . 6.0 s.u.

And 3 other PLAT232 Alerts

More ...

PLAT300_ALERT_4_G Atom Site Occupancy of N1 Constrained at 0.5 Check

And 10 other PLAT300 Alerts

More ...

PLAT302_ALERT_4_G Anion/Solvent/Minor-Residue Disorder (Resd 2) 100% Note

PLAT304_ALERT_4_G Non-Integer Number of Atoms in ..... (Resd 2) 5.50 Check

PLAT432_ALERT_2_G Short Inter X...Y Contact N1 ..C3 . 0.46 Ang.
1-x,2-y,-z = 3_675 Check

PLAT432_ALERT_2_G Short Inter X...Y Contact N1 ..C4 . 1.23 Ang.
1-x,2-y,-z = 3_675 Check

PLAT432_ALERT_2_G Short Inter X...Y Contact N1 ..C2 . 1.38 Ang.
1-x,2-y,-z = 3_675 Check

PLAT432_ALERT_2_G Short Inter X...Y Contact N1 ..C5 . 2.12 Ang.
1-x,2-y,-z = 3_675 Check
PLAT432_ALERT_2_G Short Inter X...Y Contact N1 ..C1 . 2.22 Ang.
1-x,2-y,-z = 3_675 Check
PLAT432_ALERT_2_G Short Inter X...Y Contact C1 ..C4 . 0.46 Ang.
1-x,2-y,-z = 3_675 Check
PLAT432_ALERT_2_G Short Inter X...Y Contact C1 ..C5 . 1.13 Ang.
1-x,2-y,-z = 3_675 Check
PLAT432_ALERT_2_G Short Inter X...Y Contact C1 ..C3 . 1.67 Ang.
1-x,2-y,-z = 3_675 Check
PLAT432_ALERT_2_G Short Inter X...Y Contact C1 ..C2 . 2.53 Ang.
1-x,2-y,-z = 3_675 Check
PLAT432_ALERT_2_G Short Inter X...Y Contact C1 ..C1 . 2.74 Ang.
1-x,2-y,-z = 3_675 Check
PLAT432_ALERT_2_G Short Inter X...Y Contact C2 ..C5 . 0.46 Ang.
1-x,2-y,-z = 3_675 Check
PLAT432_ALERT_2_G Short Inter X...Y Contact C2 ..C4 . 1.73 Ang.
1-x,2-y,-z = 3_675 Check
PLAT432_ALERT_2_G Short Inter X...Y Contact C2 ..C3 . 2.74 Ang.
1-x,2-y,-z = 3_675 Check
PLAT432_ALERT_2_G Short Inter X...Y Contact C2 ..C2 . 3.03 Ang.
1-x,2-y,-z = 3_675 Check
PLAT432_ALERT_2_G Short Inter X...Y Contact C3 ..C5 . 1.54 Ang.
1-x,2-y,-z = 3_675 Check
PLAT432_ALERT_2_G Short Inter X...Y Contact C3 ..C4 . 2.66 Ang.
1-x,2-y,-z = 3_675 Check
PLAT432_ALERT_2_G Short Inter X...Y Contact C3 ..C3 . 3.11 Ang.
1-x,2-y,-z = 3_675 Check
PLAT432_ALERT_2_G Short Inter X...Y Contact C4 ..C5 . 2.37 Ang.
1-x,2-y,-z = 3_675 Check
PLAT432_ALERT_2_G Short Inter X...Y Contact C4 ..C4 . 2.90 Ang.
1-x,2-y,-z = 3_675 Check
PLAT432_ALERT_2_G Short Inter X...Y Contact C5 ..C5 . 2.59 Ang.
1-x,2-y,-z = 3_675 Check
PLAT779_ALERT_4_G Suspect or Irrelevant (Bond) Angle(s) in CIF ... 29.00 Deg.
H2 -C2 -H5 1_555 1_555 3_675 ...... # 103 Check
PLAT779_ALERT_4_G Suspect or Irrelevant (Bond) Angle(s) in CIF ... 27.60 Deg.
H4 -C4 -H1 1_555 1_555 3_675 ...... # 112 Check
PLAT802_ALERT_4_G CIF Input Record(s) with more than 80 Characters 1 Info
PLAT860_ALERT_3_G Number of Least-Squares Restraints .... 6 Note
PLAT883_ALERT_1_G No Info/Value for _atom_sites_solution_primary . Please Do !
PLAT910_ALERT_3_G Missing # of FCF Reflection(s) Below Theta(Min). 1 Note

PLAT912_ALERT_4_G Missing # of FCF Reflections Above STh/L= 0.600 20 Note
PLAT978_ALERT_2_G Number C-C Bonds with Positive Residual Density. 7 Info

1 **ALERT level A** = Most likely a serious problem - resolve or explain
1 **ALERT level B** = A potentially serious problem, consider carefully
2 **ALERT level C** = Check. Ensure it is not caused by an omission or oversight
49 **ALERT level G** = General information/check it is not something unexpected

3 ALERT type 1 CIF construction/syntax error, inconsistent or missing data
28 ALERT type 2 Indicator that the structure model may be wrong or deficient
3 ALERT type 3 Indicator that the structure quality may be low
19 ALERT type 4 Improvement, methodology, query or suggestion
0 ALERT type 5 Informative message, check

It is advisable to attempt to resolve as many as possible of the alerts in all categories. Often the minor alerts point to easily fixed oversights, errors and omissions in your CIF or refinement strategy, so attention to these fine details can be worthwhile. In order to resolve some of the more serious problems it may be necessary to carry out additional measurements or structure refinements. However, the purpose of your study may justify the reported deviations and the more serious of these should normally be commented upon in the discussion or experimental section of a paper or in the "special_details" fields of the CIF. checkCIF was carefully designed to identify outliers and unusual parameters, but every test has its limitations and alerts that are not important in a particular case may appear. Conversely, the absence of alerts does not guarantee there are no aspects of the results needing attention. It is up to the individual to critically assess their own results and, if necessary, seek expert advice.

Publication of your CIF in IUCr journals

A basic structural check has been run on your CIF. These basic checks will be run on all CIFs submitted for publication in IUCr journals (*Acta Crystallographica*, *Journal of Applied Crystallography*, *Journal of Synchrotron Radiation*); however, if you intend to submit to *Acta Crystallographica Section C* or *E* or *IUCrData*, you should make sure that full publication checks are run on the final version of your CIF prior to submission.

Publication of your CIF in other journals

Please refer to the *Notes for Authors* of the relevant journal for any special instructions relating to CIF submission.

PLATON version of 12/09/2022; check.def file version of 09/08/2022

Datablock GdCl3Py4_a - ellipsoid plot

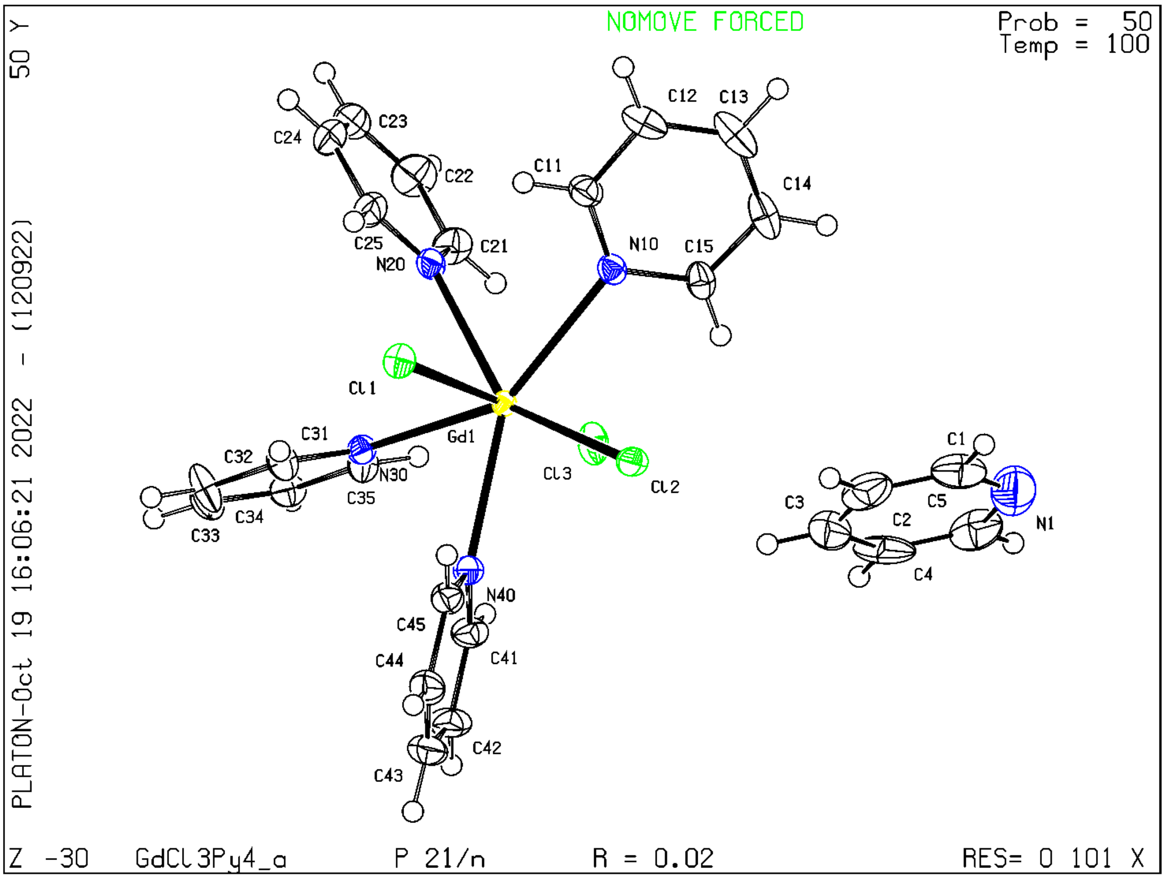

checkCIF (basic structural check) running

Checking for embedded fcf data in CIF ...
Found embedded fcf data in CIF. Extracting fcf data from uploaded CIF, please wait . . . .

checkCIF/PLATON (basic structural check)

Structure factors have been supplied for datablock(s) tbcl5_sx

THIS REPORT IS FOR GUIDANCE ONLY. IF USED AS PART OF A REVIEW PROCEDURE FOR PUBLICATION, IT SHOULD NOT REPLACE THE EXPERTISE OF AN EXPERIENCED CRYSTALLOGRAPHIC REFEREE.

No syntax errors found. CIF dictionary

Please wait while processing .... Interpreting this report

Structure factor report

Datablock: tbcl5_sx

Bond precision: C-C = 0.0085 A Wavelength=0.71073

Cell: a=18.6934(14) b=7.3235(7) c=14.8157(13)
alpha=90 beta=90 gamma=90

Temperature: 100 K

	Calculated	Reported
Volume	2028.3(3)	2028.3(3)
Space group	P n a 21	P n a 21
Hall group	P 2c -2n	P 2c -2n
Moiety formula	C5 H5 Cl5 N Tb, 2(C5 H6 N)	?
Sum formula	C15 H17 Cl5 N3 Tb	C15 H17 Cl5 N3 Tb
Mr	575.50	575.48
Dx,g cm-3	1.885	1.885
Z	4	4
Mu (mm-1)	4.148	4.148
F000	1112.0	1112.0
F000'	1114.50	
h,k,lmax	24,9,19	24,9,19
Nref	4658[2420]	4604
Tmin,Tmax	0.242,0.492	0.224,0.526
Tmin'	0.117	

Correction method= # Reported T Limits: Tmin=0.224 Tmax=0.526 AbsCorr = MULTI-SCAN

Data completeness= 1.90/0.99 Theta(max)= 27.481

R(reflections)= 0.0178(4385) wR2(reflections)= 0.0371(4604)

S = 1.029 Npar= 226

The following ALERTS were generated. Each ALERT has the format

test-name_ALERT_alert-type_alert-level.

Click on the hyperlinks for more details of the test.

Alert level C

ABSTY02_ALERT_1_C An _exptl_absorpt_correction_type has been given without a literature citation. This should be contained in the _exptl_absorpt_process_details field.
Absorption correction given as multi-scan

STRVA01_ALERT_4_C Flack test results are ambiguous.
From the CIF: _refine_ls_abs_structure_Flack 0.500
From the CIF: _refine_ls_abs_structure_Flack_su 0.009

PLAT342_ALERT_3_C Low Bond Precision on C-C Bonds .... 0.0085 Ang.

PLAT352_ALERT_3_C Short N-H (X0.87,N1.01A) N20 - H20 . 0.67 Ang.
PLAT352_ALERT_3_C Short N-H (X0.87,N1.01A) N30 - H30 . 0.73 Ang.
PLAT911_ALERT_3_C Missing FCF Refl Between Thmin & STh/L= 0.600 6 Report

Alert level G

PLAT111_ALERT_2_G ADDSYM Detects New (Pseudo) Centre of Symmetry . 100 %Fit
PLAT113_ALERT_2_G ADDSYM Suggests Possible Pseudo/New Space Group Pnma Check
Check Model Parameter Symmmetry for Reflection Data Support
PLAT794_ALERT_5_G Tentative Bond Valency for Tb1 (III) . 3.21 Info
PLAT883_ALERT_1_G No Info/Value for _atom_sites_solution_primary . Please Do !
PLAT912_ALERT_4_G Missing # of FCF Reflections Above STh/L= 0.600 2 Note
PLAT913_ALERT_3_G Missing # of Very Strong Reflections in FCF .... 2 Note
PLAT978_ALERT_2_G Number C-C Bonds with Positive Residual Density. 3 Info

0 **ALERT level A** = Most likely a serious problem - resolve or explain
0 **ALERT level B** = A potentially serious problem, consider carefully
6 **ALERT level C** = Check. Ensure it is not caused by an omission or oversight
7 **ALERT level G** = General information/check it is not something unexpected

2 ALERT type 1 CIF construction/syntax error, inconsistent or missing data
3 ALERT type 2 Indicator that the structure model may be wrong or deficient
5 ALERT type 3 Indicator that the structure quality may be low
2 ALERT type 4 Improvement, methodology, query or suggestion
1 ALERT type 5 Informative message, check

It is advisable to attempt to resolve as many as possible of the alerts in all categories. Often the minor alerts point to easily fixed oversights, errors and omissions in your CIF or refinement strategy, so attention to these fine details can be worthwhile. In order to resolve some of the more serious problems it may be necessary to carry out additional measurements or structure refinements. However, the purpose of your study may justify the reported deviations and the more serious of these should normally be commented upon in the discussion or experimental section of a paper or in the "special_details" fields of the CIF. checkCIF was carefully designed to identify outliers and unusual parameters, but every test has its limitations and alerts that are not important in a particular case may appear. Conversely, the absence of alerts does not guarantee there are no aspects of the results needing attention. It is up to the individual to critically assess their own results and, if necessary, seek expert advice.

Publication of your CIF in IUCr journals

A basic structural check has been run on your CIF. These basic checks will be run on all CIFs submitted for publication in IUCr journals (*Acta Crystallographica*, *Journal of Applied Crystallography*, *Journal of Synchrotron Radiation*); however, if you intend to submit to *Acta Crystallographica Section C* or *E* or *IUCrData*, you should make sure that full publication checks are run on the final version of your CIF prior to submission.

Publication of your CIF in other journals

Please refer to the *Notes for Authors* of the relevant journal for any special instructions relating to CIF submission.

PLATON version of 12/09/2022; check.def file version of 09/08/2022

Datablock tbcl5_sx - ellipsoid plot

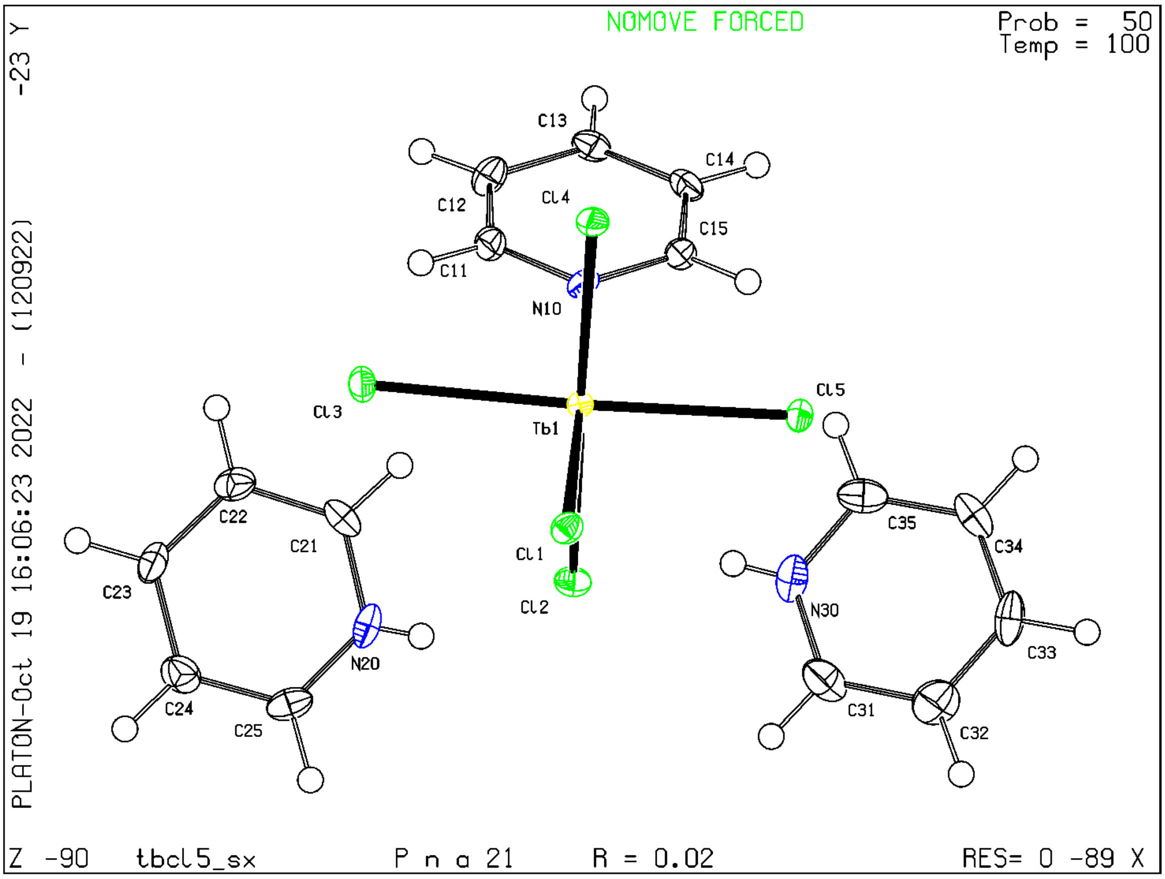

checkCIF (basic structural check) running

Checking for embedded fcf data in CIF ...
Found embedded fcf data in CIF. Extracting fcf data from uploaded CIF, please wait . . . . .

checkCIF/PLATON (basic structural check)

Structure factors have been supplied for datablock(s) DyCl5_d

THIS REPORT IS FOR GUIDANCE ONLY. IF USED AS PART OF A REVIEW PROCEDURE FOR PUBLICATION, IT SHOULD NOT REPLACE THE EXPERTISE OF AN EXPERIENCED CRYSTALLOGRAPHIC REFEREE.

No syntax errors found. CIF dictionary

Please wait while processing .... Interpreting this report

Structure factor report

Datablock: DyCl5_d

Bond precision:	C-C = 0.0048 A		Wavelength=0.71073
Cell:	a=18.693(3)	b=7.3078(10)	c=14.789(2)
	alpha=90	beta=90	gamma=90
Temperature: 100 K			

	Calculated	Reported
Volume	2020.3(5)	2020.3(5)
Space group	P n a 21	P n a 21
Hall group	P 2c -2n	P 2c -2n
Moiety formula	C5 H5 Cl5 Dy N, 2(C5 H6 N)	?
Sum formula	C15 H17 Cl5 Dy N3	C15 H17 Cl5 Dy N3
Mr	579.07	579.06
Dx,g cm-3	1.904	1.904
Z	4	4
Mu (mm-1)	4.362	4.362
F000	1116.0	1116.0
F000'	1118.44	
h,k,lmax	26,10,21	26,10,21
Nref	6195[3207]	6181
Tmin,Tmax	0.391,0.442	0.621,0.746
Tmin'	0.362	

Correction method= # Reported T Limits: Tmin=0.621 Tmax=0.746 AbsCorr = MULTI-SCAN

Data completeness= 1.93/1.00 Theta(max)= 30.547

R(reflections)= 0.0137(6125) wR2(reflections)= 0.0330(6181)

S = 1.157 Npar= 226

The following ALERTS were generated. Each ALERT has the format

test-name_ALERT_alert-type_alert-level.

Click on the hyperlinks for more details of the test.

Alert level C

ABSTY02_ALERT_1_C An _exptl_absorpt_correction_type has been given without a literature citation. This should be contained in the _exptl_absorpt_process_details field.

Absorption correction given as multi-scan

STRVA01_ALERT_4_C Flack test results are ambiguous.

From the CIF: _refine_ls_abs_structure_Flack 0.459

From the CIF: _refine_ls_abs_structure_Flack_su 0.006

PLAT352_ALERT_3_C Short N-H (X0.87,N1.01A) N30 - H30 . 0.73 Ang.

Alert level G

PLAT111_ALERT_2_G ADDSYM Detects New (Pseudo) Centre of Symmetry . 100 %Fit
PLAT113_ALERT_2_G ADDSYM Suggests Possible Pseudo/New Space Group Pnma Check
Check Model Parameter Symmmetry for Reflection Data Support
PLAT232_ALERT_2_G Hirshfeld Test Diff (M-X) Dy1 --Cl4 . 6.5 s.u.
PLAT883_ALERT_1_G No Info/Value for _atom_sites_solution_primary . Please Do !
PLAT912_ALERT_4_G Missing # of FCF Reflections Above STh/L= 0.600 2 Note
PLAT978_ALERT_2_G Number C-C Bonds with Positive Residual Density. 4 Info

0 **ALERT level A** = Most likely a serious problem - resolve or explain
0 **ALERT level B** = A potentially serious problem, consider carefully
3 **ALERT level C** = Check. Ensure it is not caused by an omission or oversight
6 **ALERT level G** = General information/check it is not something unexpected

2 ALERT type 1 CIF construction/syntax error, inconsistent or missing data
4 ALERT type 2 Indicator that the structure model may be wrong or deficient
1 ALERT type 3 Indicator that the structure quality may be low
2 ALERT type 4 Improvement, methodology, query or suggestion
0 ALERT type 5 Informative message, check

It is advisable to attempt to resolve as many as possible of the alerts in all categories. Often the minor alerts point to easily fixed oversights, errors and omissions in your CIF or refinement strategy, so attention to these fine details can be worthwhile. In order to resolve some of the more serious problems it may be necessary to carry out additional measurements or structure refinements. However, the purpose of your study may justify the reported deviations and the more serious of these should normally be commented upon in the discussion or experimental section of a paper or in the "special_details" fields of the CIF. checkCIF was carefully designed to identify outliers and unusual parameters, but every test has its limitations and alerts that are not important in a particular case may appear. Conversely, the absence of alerts does not guarantee there are no aspects of the results needing attention. It is up to the individual to critically assess their own results and, if necessary, seek expert advice.

Publication of your CIF in IUCr journals

A basic structural check has been run on your CIF. These basic checks will be run on all CIFs submitted for publication in IUCr journals (*Acta Crystallographica*, *Journal of Applied Crystallography*, *Journal of Synchrotron Radiation*); however, if you intend to submit to *Acta Crystallographica Section C* or *E* or *IUCrData*, you should make sure that full publication checks are run on the final version of your CIF prior to submission.

Publication of your CIF in other journals

Please refer to the *Notes for Authors* of the relevant journal for any special instructions relating to CIF submission.

PLATON version of 28/11/2022; check.def file version of 28/11/2022

Datablock DyCl5_d - ellipsoid plot

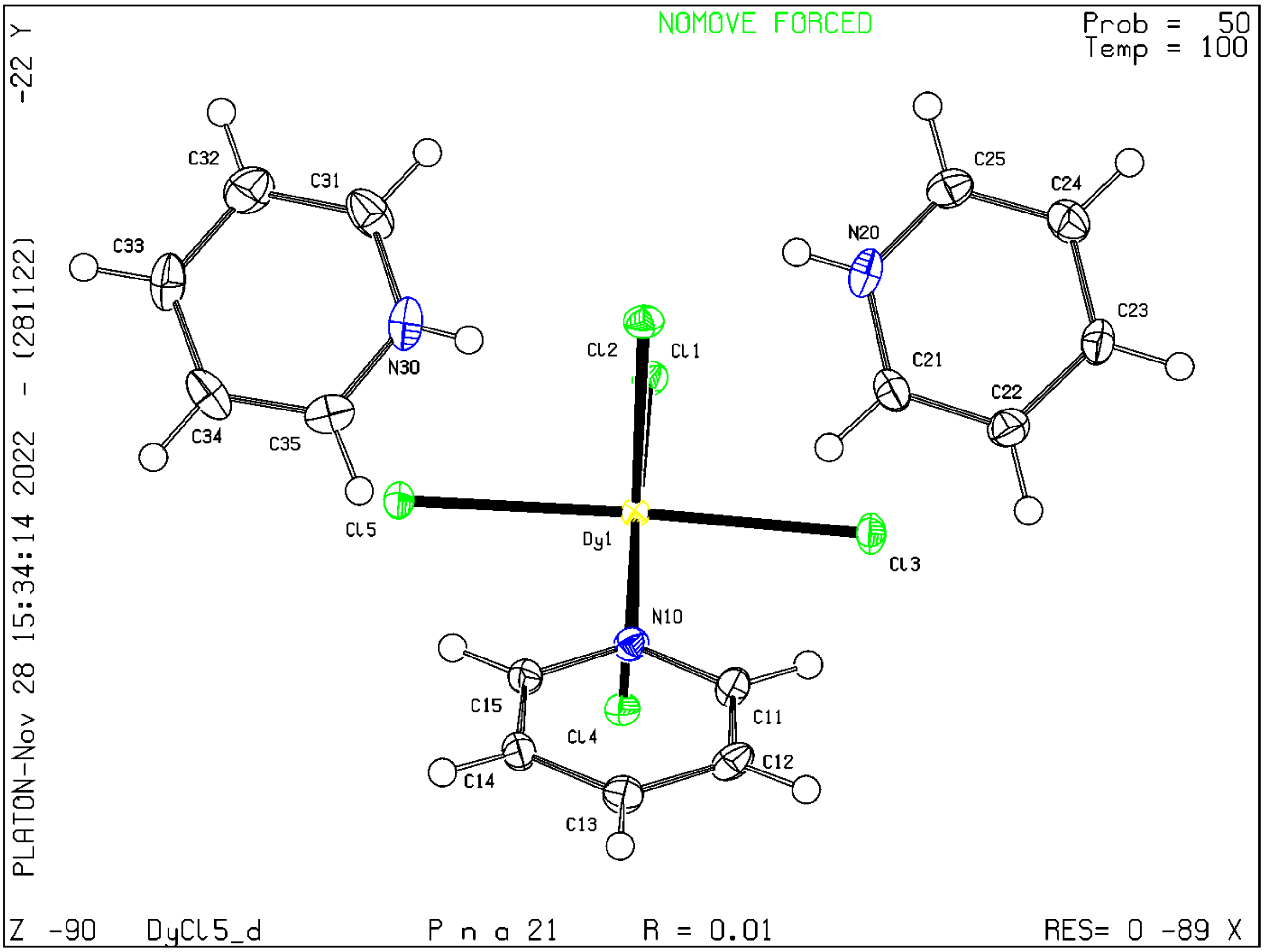

checkCIF (basic structural check) running

Checking for embedded fcf data in CIF ...
Found embedded fcf data in CIF. Extracting fcf data from uploaded CIF, please wait . . . . . .

checkCIF/PLATON (basic structural check)

Structure factors have been supplied for datablock(s) HoCl5_d

THIS REPORT IS FOR GUIDANCE ONLY. IF USED AS PART OF A REVIEW PROCEDURE FOR PUBLICATION, IT SHOULD NOT REPLACE THE EXPERTISE OF AN EXPERIENCED CRYSTALLOGRAPHIC REFEREE.

No syntax errors found. CIF dictionary

Please wait while processing .... Interpreting this report

Datablock: HoCl5_d

Bond precision:	C-C = 0.0042 A		Wavelength=0.71073
Cell:	a=18.6738(17)	b=7.2976(6)	c=14.7913(13)
	alpha=90	beta=90	gamma=90
Temperature:	100 K		

	Calculated	Reported
Volume	2015.7(3)	2015.7(3)
Space group	P n a 21	P n a 21
Hall group	P 2c -2n	P 2c -2n
Moiety formula	C5 H5 Cl5 Ho N, 2(C5 H6 N)	?
Sum formula	C15 H17 Cl5 Ho N3	C15 H17 Cl5 Ho N3
Mr	581.50	581.49
Dx,g cm-3	1.916	1.916
Z	4	4
Mu (mm-1)	4.590	4.590
F000	1120.0	1120.0
F000'	1122.33	
h,k,lmax	32,12,25	31,12,25
Nref	10776[5547]	10092
Tmin,Tmax	0.194,0.350	0.240,0.437
Tmin'	0.111	

Correction method= # Reported T Limits: Tmin=0.240 Tmax=0.437 AbsCorr = MULTI-SCAN

Data completeness= 1.82/0.94 Theta(max)= 37.702

R(reflections)= 0.0165(9882) wR2(reflections)= 0.0379(10092)

S = 1.106 Npar= 226

The following ALERTS were generated. Each ALERT has the format

test-name_ALERT_alert-type_alert-level.

Click on the hyperlinks for more details of the test.

Alert level C

ABSTY02_ALERT_1_C An _exptl_absorpt_correction_type has been given without a literature citation. This should be contained in the _exptl_absorpt_process_details field.

Absorption correction given as multi-scan

STRVA01_ALERT_4_C Flack test results are ambiguous.

From the CIF: _refine_ls_abs_structure_Flack 0.465

From the CIF: _refine_ls_abs_structure_Flack_su 0.005

PLAT911_ALERT_3_C Missing FCF Refl Between Thmin & STh/L= 0.600 7 Report
PLAT913_ALERT_3_C Missing # of Very Strong Reflections in FCF .... 4 Note

Alert level G

PLAT111_ALERT_2_G ADDSYM Detects New (Pseudo) Centre of Symmetry . 100 %Fit
PLAT113_ALERT_2_G ADDSYM Suggests Possible Pseudo/New Space Group Pnma Check
Check Model Parameter Symmmetry for Reflection Data Support
PLAT232_ALERT_2_G Hirshfeld Test Diff (M-X) Ho --Cl1 . 10.2 s.u.
And 4 other PLAT232 Alerts
More ...
PLAT883_ALERT_1_G No Info/Value for _atom_sites_solution_primary . Please Do !
PLAT912_ALERT_4_G Missing # of FCF Reflections Above STh/L= 0.600 193 Note
PLAT978_ALERT_2_G Number C-C Bonds with Positive Residual Density. 4 Info

0 **ALERT level A** = Most likely a serious problem - resolve or explain
0 **ALERT level B** = A potentially serious problem, consider carefully
4 **ALERT level C** = Check. Ensure it is not caused by an omission or oversight
10 **ALERT level G** = General information/check it is not something unexpected

2 ALERT type 1 CIF construction/syntax error, inconsistent or missing data
8 ALERT type 2 Indicator that the structure model may be wrong or deficient
2 ALERT type 3 Indicator that the structure quality may be low
2 ALERT type 4 Improvement, methodology, query or suggestion
0 ALERT type 5 Informative message, check

It is advisable to attempt to resolve as many as possible of the alerts in all categories. Often the minor alerts point to easily fixed oversights, errors and omissions in your CIF or refinement strategy, so attention to these fine details can be worthwhile. In order to resolve some of the more serious problems it may be necessary to carry out additional measurements or structure refinements. However, the purpose of your study may justify the reported deviations and the more serious of these should normally be commented upon in the discussion or experimental section of a paper or in the "special_details" fields of the CIF. checkCIF was carefully designed to identify outliers and unusual parameters, but every test has its limitations and alerts that are not important in a particular case may appear. Conversely, the absence of alerts does not guarantee there are no aspects of the results needing attention. It is up to the individual to critically assess their own results and, if necessary, seek expert advice.

Publication of your CIF in IUCr journals

A basic structural check has been run on your CIF. These basic checks will be run on all CIFs submitted for publication in IUCr journals (*Acta Crystallographica*, *Journal of Applied Crystallography*, *Journal of Synchrotron Radiation*); however, if you intend to submit to *Acta Crystallographica Section C* or *E* or *IUCrData*, you should make sure that full publication checks are run on the final version of your CIF prior to submission.

Publication of your CIF in other journals

Please refer to the *Notes for Authors* of the relevant journal for any special instructions relating to CIF submission.

PLATON version of 12/09/2022; check.def file version of 09/08/2022

Datablock HoCl5_d - ellipsoid plot

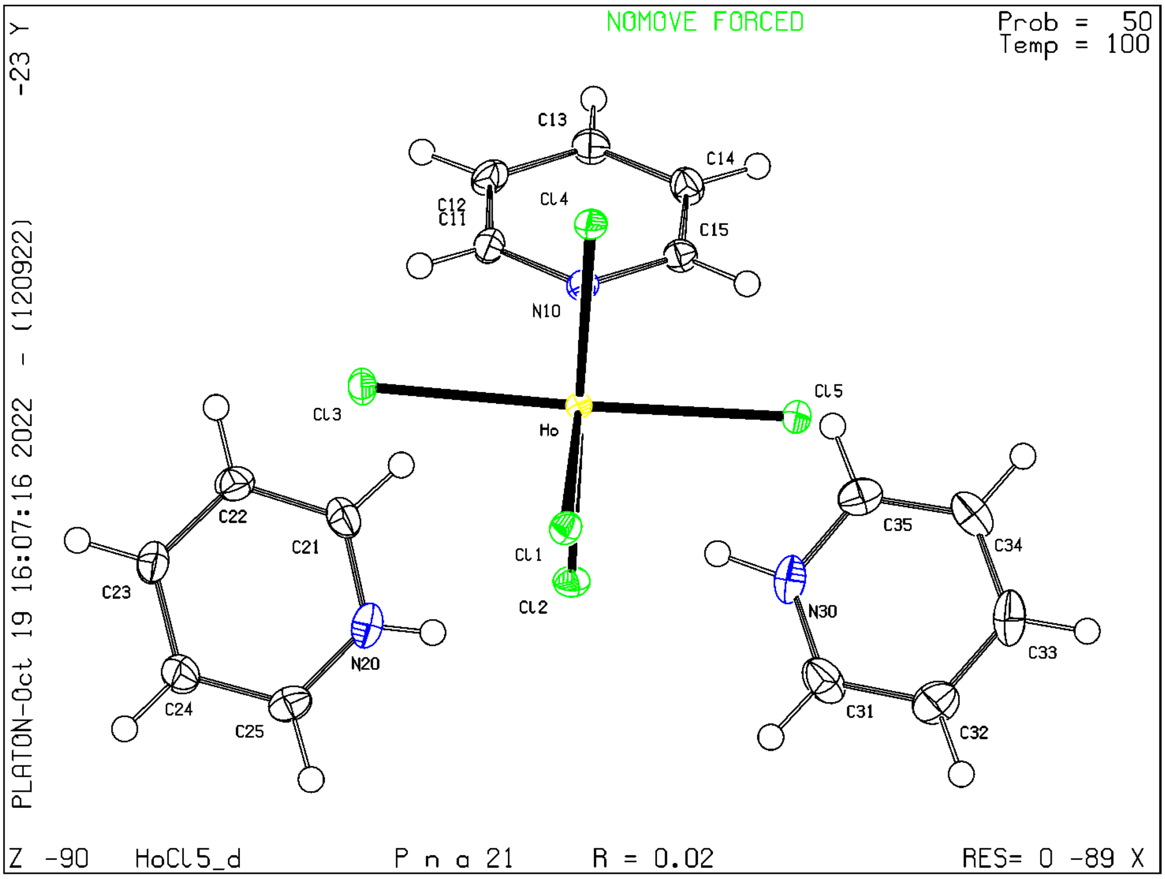

checkCIF (basic structural check) running

Checking for embedded fcf data in CIF ...
Found embedded fcf data in CIF. Extracting fcf data from uploaded CIF, please wait . . . . .

checkCIF/PLATON (basic structural check)

Structure factors have been supplied for datablock(s) ErCl5_d

THIS REPORT IS FOR GUIDANCE ONLY. IF USED AS PART OF A REVIEW PROCEDURE FOR PUBLICATION, IT SHOULD NOT REPLACE THE EXPERTISE OF AN EXPERIENCED CRYSTALLOGRAPHIC REFEREE.

No syntax errors found. CIF dictionary

Please wait while processing .... Interpreting this report

Structure factor report

Datablock: ErCl5_d

Bond precision: C-C = 0.0052 A Wavelength=0.71073

Cell: a=18.6315(7) b=7.2898(3) c=14.7705(5)
alpha=90 beta=90 gamma=90

Temperature: 100 K

	Calculated	Reported
Volume	2006.13(13)	2006.13(13)
Space group	P n a 21	P n a 21
Hall group	P 2c -2n	P 2c -2n
Moiety formula	C5 H5 Cl5 Er N, 2(C5 H6 N)	?
Sum formula	C15 H17 Cl5 Er N3	C15 H17 Cl5 Er N3
Mr	583.83	583.82
Dx,g cm-3	1.933	1.933
Z	4	4
Mu (mm-1)	4.852	4.852
F000	1124.0	1124.0
F000'	1126.20	
h,k,lmax	24,9,19	24,9,19
Nref	4660[2421]	4605
Tmin,Tmax	0.156,0.309	0.384,0.604
Tmin'	0.078	

Correction method= # Reported T Limits: Tmin=0.384 Tmax=0.604 AbsCorr = MULTI-SCAN

Data completeness= 1.90/0.99 Theta(max)= 27.590

R(reflections)= 0.0105(4587) wR2(reflections)= 0.0268(4605)

S = 1.120 Npar= 226

The following ALERTS were generated. Each ALERT has the format

test-name_ALERT_alert-type_alert-level.

Click on the hyperlinks for more details of the test.

Alert level C

ABSTY02_ALERT_1_C An _exptl_absorpt_correction_type has been given without a literature citation. This should be contained in the _exptl_absorpt_process_details field.
Absorption correction given as multi-scan

STRVA01_ALERT_4_C Flack test results are ambiguous.
From the CIF: _refine_ls_abs_structure_Flack 0.486
From the CIF: _refine_ls_abs_structure_Flack_su 0.005

PLAT352_ALERT_3_C Short N-H (X0.87,N1.01A) N30 - H30 . 0.70 Ang.

PLAT911_ALERT_3_C Missing FCF Refl Between Thmin & STh/L= 0.600 2 Report

Alert level G

PLAT111_ALERT_2_G ADDSYM Detects New (Pseudo) Centre of Symmetry . 100 %Fit

PLAT113_ALERT_2_G ADDSYM Suggests Possible Pseudo/New Space Group Pnma Check

Check Model Parameter Symmmetry for Reflection Data Support

PLAT883_ALERT_1_G No Info/Value for _atom_sites_solution_primary . Please Do !

PLAT912_ALERT_4_G Missing # of FCF Reflections Above STh/L= 0.600 3 Note

PLAT978_ALERT_2_G Number C-C Bonds with Positive Residual Density. 6 Info

0 **ALERT level A** = Most likely a serious problem - resolve or explain

0 **ALERT level B** = A potentially serious problem, consider carefully

4 **ALERT level C** = Check. Ensure it is not caused by an omission or oversight

5 **ALERT level G** = General information/check it is not something unexpected

2 ALERT type 1 CIF construction/syntax error, inconsistent or missing data

3 ALERT type 2 Indicator that the structure model may be wrong or deficient

2 ALERT type 3 Indicator that the structure quality may be low

2 ALERT type 4 Improvement, methodology, query or suggestion

0 ALERT type 5 Informative message, check

It is advisable to attempt to resolve as many as possible of the alerts in all categories. Often the minor alerts point to easily fixed oversights, errors and omissions in your CIF or refinement strategy, so attention to these fine details can be worthwhile. In order to resolve some of the more serious problems it may be necessary to carry out additional measurements or structure refinements. However, the purpose of your study may justify the reported deviations and the more serious of these should normally be commented upon in the discussion or experimental section of a paper or in the "special_details" fields of the CIF. checkCIF was carefully designed to identify outliers and unusual parameters, but every test has its limitations and alerts that are not important in a particular case may appear. Conversely, the absence of alerts does not guarantee there are no aspects of the results needing attention. It is up to the individual to critically assess their own results and, if necessary, seek expert advice.

Publication of your CIF in IUCr journals

A basic structural check has been run on your CIF. These basic checks will be run on all CIFs submitted for publication in IUCr journals (*Acta Crystallographica*, *Journal of Applied Crystallography*, *Journal of Synchrotron Radiation*); however, if you intend to submit to *Acta Crystallographica Section C* or *E* or *IUCrData*, you should make sure that full publication checks are run on the final version of your CIF prior to submission.

Publication of your CIF in other journals

Please refer to the *Notes for Authors* of the relevant journal for any special instructions relating to CIF submission.

PLATON version of 28/11/2022; check.def file version of 28/11/2022

Datablock ErCl5_d - ellipsoid plot

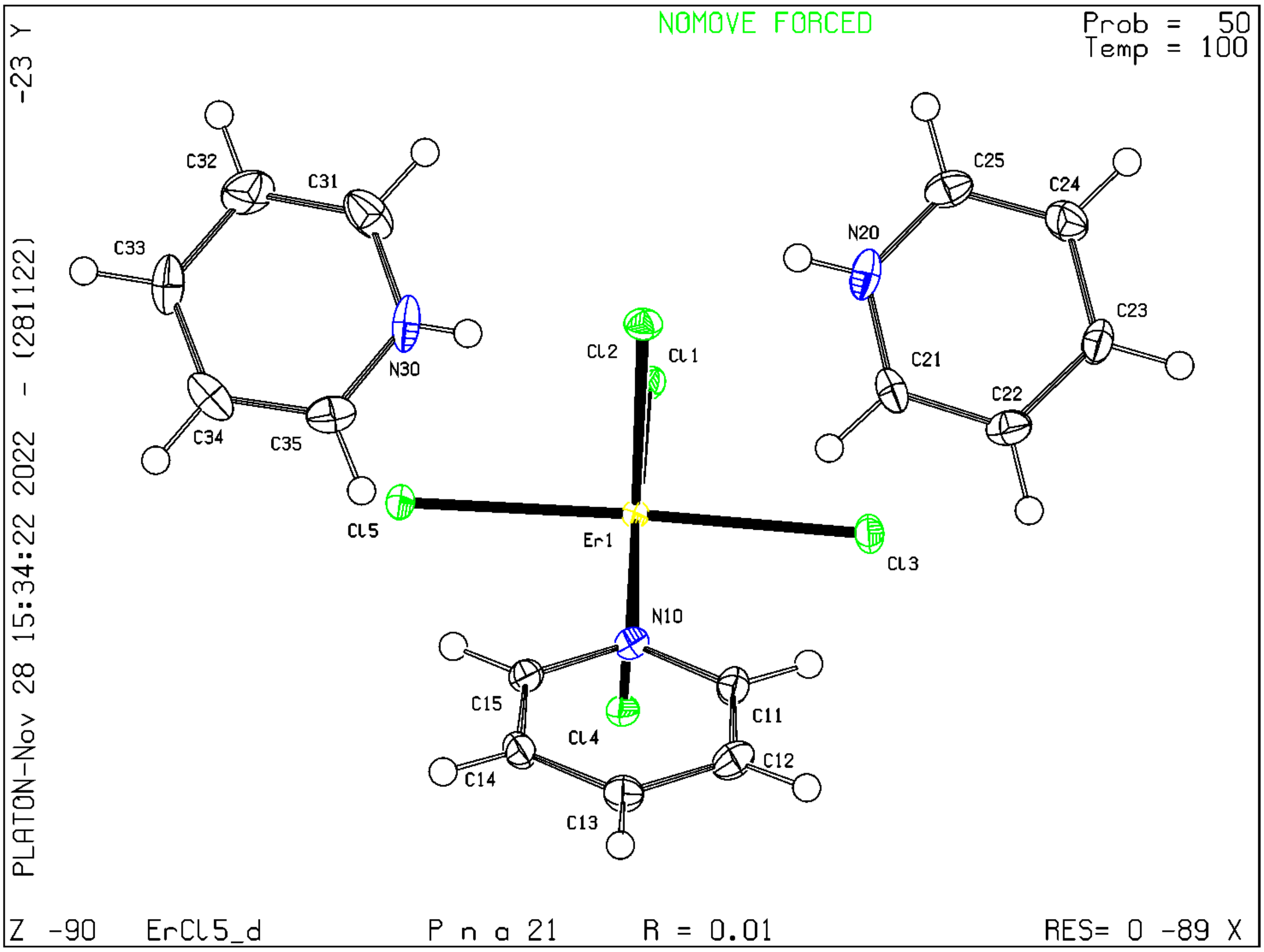

checkCIF (basic structural check) running

Checking for embedded fcf data in CIF ...
Found embedded fcf data in CIF. Extracting fcf data from uploaded CIF, please wait . . . .

checkCIF/PLATON (basic structural check)

Structure factors have been supplied for datablock(s) TmCl5_d

THIS REPORT IS FOR GUIDANCE ONLY. IF USED AS PART OF A REVIEW PROCEDURE FOR PUBLICATION, IT SHOULD NOT REPLACE THE EXPERTISE OF AN EXPERIENCED CRYSTALLOGRAPHIC REFEREE.

No syntax errors found. CIF dictionary

Please wait while processing .... Interpreting this report

Structure factor report

Datablock: TmCl5_d

Bond precision:	C-C = 0.0082 A		Wavelength=0.71073
Cell:	a=18.6333(11)	b=7.2815(4)	c=14.7648(9)
	alpha=90	beta=90	gamma=90
Temperature:	100 K		

	Calculated	Reported
Volume	2003.3(2)	2003.3(2)
Space group	P n a 21	P n a 21
Hall group	P 2c -2n	P 2c -2n
Moiety formula	C5 H5 Cl5 N Tm, 2(C5 H6 N)	?
Sum formula	C15 H17 Cl5 N3 Tm	C15 H17 Cl5 N3 Tm
Mr	585.50	585.49
Dx,g cm-3	1.941	1.941
Z	4	4
Mu (mm-1)	5.098	5.098
F000	1128.0	1128.0
F000'	1130.00	
h,k,lmax	30,11,23	30,11,23
Nref	8958[4619]	8216
Tmin,Tmax	0.118,0.316	0.209,0.392
Tmin'	0.089	

Correction method= # Reported T Limits: Tmin=0.209 Tmax=0.392 AbsCorr = MULTI-SCAN

Data completeness= 1.78/0.92 Theta(max)= 35.196

R(reflections)= 0.0227(7713) wR2(reflections)= 0.0528(8216)

S = 1.232 Npar= 226

The following ALERTS were generated. Each ALERT has the format

test-name_ALERT_alert-type_alert-level.

Click on the hyperlinks for more details of the test.

Alert level C

ABSTY02_ALERT_1_C An _exptl_absorpt_correction_type has been given without a literature citation. This should be contained in the _exptl_absorpt_process_details field.

Absorption correction given as multi-scan

STRVA01_ALERT_4_C Flack test results are ambiguous.

From the CIF: _refine_ls_abs_structure_Flack 0.350

From the CIF: _refine_ls_abs_structure_Flack_su 0.008
PLAT342_ALERT_3_C Low Bond Precision on C-C Bonds .... 0.00817 Ang.
PLAT352_ALERT_3_C Short N-H (X0.87,N1.01A) N20 - H20 . 0.74 Ang.
PLAT352_ALERT_3_C Short N-H (X0.87,N1.01A) N30 - H30 . 0.74 Ang.
PLAT911_ALERT_3_C Missing FCF Refl Between Thmin & STh/L= 0.600 4 Report
PLAT975_ALERT_2_C Check Calcd Resid. Dens. 0.78Ang From N10 . 0.52 eA-3

Alert level G

PLAT111_ALERT_2_G ADDSYM Detects New (Pseudo) Centre of Symmetry . 100 %Fit
PLAT113_ALERT_2_G ADDSYM Suggests Possible Pseudo/New Space Group Pnma Check
Check Model Parameter Symmmetry for Reflection Data Support
PLAT794_ALERT_5_G Tentative Bond Valency for Tm1 (III) . 3.19 Info
PLAT883_ALERT_1_G No Info/Value for _atom_sites_solution_primary . Please Do !
PLAT912_ALERT_4_G Missing # of FCF Reflections Above STh/L= 0.600 221 Note
PLAT913_ALERT_3_G Missing # of Very Strong Reflections in FCF .... 2 Note
PLAT978_ALERT_2_G Number C-C Bonds with Positive Residual Density. 0 Info

0 **ALERT level A** = Most likely a serious problem - resolve or explain
0 **ALERT level B** = A potentially serious problem, consider carefully
7 **ALERT level C** = Check. Ensure it is not caused by an omission or oversight
7 **ALERT level G** = General information/check it is not something unexpected

2 ALERT type 1 CIF construction/syntax error, inconsistent or missing data
4 ALERT type 2 Indicator that the structure model may be wrong or deficient
5 ALERT type 3 Indicator that the structure quality may be low
2 ALERT type 4 Improvement, methodology, query or suggestion
1 ALERT type 5 Informative message, check

It is advisable to attempt to resolve as many as possible of the alerts in all categories. Often the minor alerts point to easily fixed oversights, errors and omissions in your CIF or refinement strategy, so attention to these fine details can be worthwhile. In order to resolve some of the more serious problems it may be necessary to carry out additional measurements or structure refinements. However, the purpose of your study may justify the reported deviations and the more serious of these should normally be commented upon in the discussion or experimental section of a paper or in the "special_details" fields of the CIF. checkCIF was carefully designed to identify outliers and unusual parameters, but every test has its limitations and alerts that are not important in a particular case may appear. Conversely, the absence of alerts does not guarantee there are no aspects of the results needing attention. It is up to the individual to critically assess their own results and, if necessary, seek expert advice.

Publication of your CIF in IUCr journals

A basic structural check has been run on your CIF. These basic checks will be run on all CIFs submitted for publication in IUCr journals (*Acta Crystallographica*, *Journal of Applied Crystallography*, *Journal of Synchrotron Radiation*); however, if you intend to submit to *Acta Crystallographica Section C* or *E* or *IUCrData*, you should make sure that full publication checks are run on the final version of your CIF prior to submission.

Publication of your CIF in other journals

Please refer to the *Notes for Authors* of the relevant journal for any special instructions relating to CIF submission.

PLATON version of 12/09/2022; check.def file version of 09/08/2022

Datablock TmCl5_d - ellipsoid plot

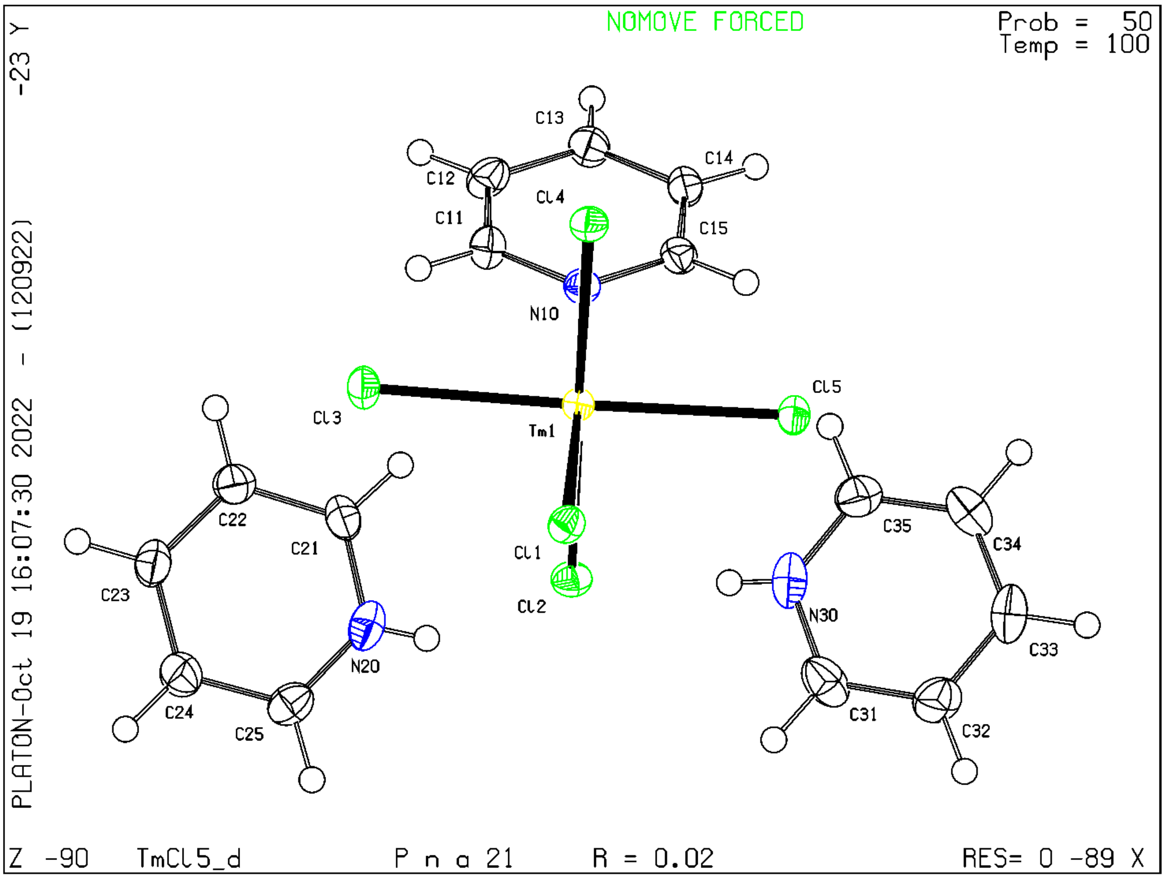

checkCIF (basic structural check) running

Checking for embedded fcf data in CIF ...
Found embedded fcf data in CIF. Extracting fcf data from uploaded CIF, please wait . . . . . .

checkCIF/PLATON (basic structural check)

Structure factors have been supplied for datablock(s) YbCl5Py

THIS REPORT IS FOR GUIDANCE ONLY. IF USED AS PART OF A REVIEW PROCEDURE FOR PUBLICATION, IT SHOULD NOT REPLACE THE EXPERTISE OF AN EXPERIENCED CRYSTALLOGRAPHIC REFEREE.

No syntax errors found. CIF dictionary

Please wait while processing .... Interpreting this report

Datablock: YbCl5Py

Bond precision:	C-C = 0.0077 A		Wavelength=0.71073
Cell:	a=18.6135(11)	b=7.2804(4)	c=14.7520(9)
	alpha=90	beta=90	gamma=90
Temperature:	100 K		

	Calculated	Reported
Volume	1999.1(2)	1999.1(2)
Space group	P n a 21	P n a 21
Hall group	P 2c -2n	P 2c -2n
Moiety formula	C5 H5 Cl5 N Yb, 2(C5 H6 N)	?
Sum formula	C15 H17 Cl5 N3 Yb	C15 H17 Cl5 N3 Yb
Mr	589.61	589.60
Dx,g cm-3	1.959	1.959
Z	4	4
Mu (mm-1)	5.349	5.349
F000	1132.0	1132.0
F000'	1133.73	
h,k,lmax	29,11,23	28,11,23
Nref	8402[4335]	7693
Tmin,Tmax	0.138,0.187	0.450,0.747
Tmin'	0.070	

Correction method= # Reported T Limits: Tmin=0.450 Tmax=0.747 AbsCorr = MULTI-SCAN

Data completeness= 1.77/0.92 Theta(max)= 34.376

R(reflections)= 0.0216(7376) wR2(reflections)= 0.0481(7693)

S = 1.123 Npar= 218

The following ALERTS were generated. Each ALERT has the format

test-name_ALERT_alert-type_alert-level.

Click on the hyperlinks for more details of the test.

Alert level B

PLAT974_ALERT_2_B Check Calcd Negative Resid. Density on Yb1 -1.54 eA-3

Alert level C

ABSTY02_ALERT_1_C An _exptl_absorpt_correction_type has been given without a literature citation. This should be contained in the _exptl_absorpt_process_details field.

Absorption correction given as multi-scan

STRVA01_ALERT_4_C Flack test results are ambiguous.

From the CIF: _refine_ls_abs_structure_Flack 0.457

From the CIF: _refine_ls_abs_structure_Flack_su 0.007

PLAT911_ALERT_3_C Missing FCF Refl Between Thmin & STh/L= 0.600 6 Report

Alert level G

PLAT007_ALERT_5_G Number of Unrefined Donor-H Atoms .... 2 Report

PLAT111_ALERT_2_G ADDSYM Detects New (Pseudo) Centre of Symmetry . 100 %Fit

PLAT113_ALERT_2_G ADDSYM Suggests Possible Pseudo/New Space Group Pnma Check

Check Model Parameter Symmmetry for Reflection Data Support

PLAT794_ALERT_5_G Tentative Bond Valency for Yb1 (III) . 3.18 Info

PLAT883_ALERT_1_G No Info/Value for _atom_sites_solution_primary . Please Do !

PLAT912_ALERT_4_G Missing # of FCF Reflections Above STh/L= 0.600 204 Note

PLAT913_ALERT_3_G Missing # of Very Strong Reflections in FCF .... 1 Note

PLAT978_ALERT_2_G Number C-C Bonds with Positive Residual Density. 0 Info

0 **ALERT level A** = Most likely a serious problem - resolve or explain

1 **ALERT level B** = A potentially serious problem, consider carefully

3 **ALERT level C** = Check. Ensure it is not caused by an omission or oversight

8 **ALERT level G** = General information/check it is not something unexpected

2 ALERT type 1 CIF construction/syntax error, inconsistent or missing data

4 ALERT type 2 Indicator that the structure model may be wrong or deficient

2 ALERT type 3 Indicator that the structure quality may be low

2 ALERT type 4 Improvement, methodology, query or suggestion

2 ALERT type 5 Informative message, check

It is advisable to attempt to resolve as many as possible of the alerts in all categories. Often the minor alerts point to easily fixed oversights, errors and omissions in your CIF or refinement strategy, so attention to these fine details can be worthwhile. In order to resolve some of the more serious problems it may be necessary to carry out additional measurements or structure refinements. However, the purpose of your study may justify the reported deviations and the more serious of these should normally be commented upon in the discussion or experimental section of a paper or in the "special_details" fields of the CIF. checkCIF was carefully designed to identify outliers and unusual parameters, but every test has its limitations and alerts that are not important in a particular case may appear. Conversely, the absence of alerts does not guarantee there are no aspects of the results needing attention. It is up to the individual to critically assess their own results and, if necessary, seek expert advice.

Publication of your CIF in IUCr journals

A basic structural check has been run on your CIF. These basic checks will be run on all CIFs submitted for publication in IUCr journals (*Acta Crystallographica*, *Journal of Applied Crystallography*, *Journal of Synchrotron Radiation*); however, if you intend to submit to *Acta Crystallographica Section C* or *E* or *IUCrData*, you should make sure that full publication checks are run on the final version of your CIF prior to submission.

Publication of your CIF in other journals

Please refer to the *Notes for Authors* of the relevant journal for any special instructions relating to CIF submission.

PLATON version of 28/11/2022; check.def file version of 28/11/2022

Datablock YbCl5Py - ellipsoid plot

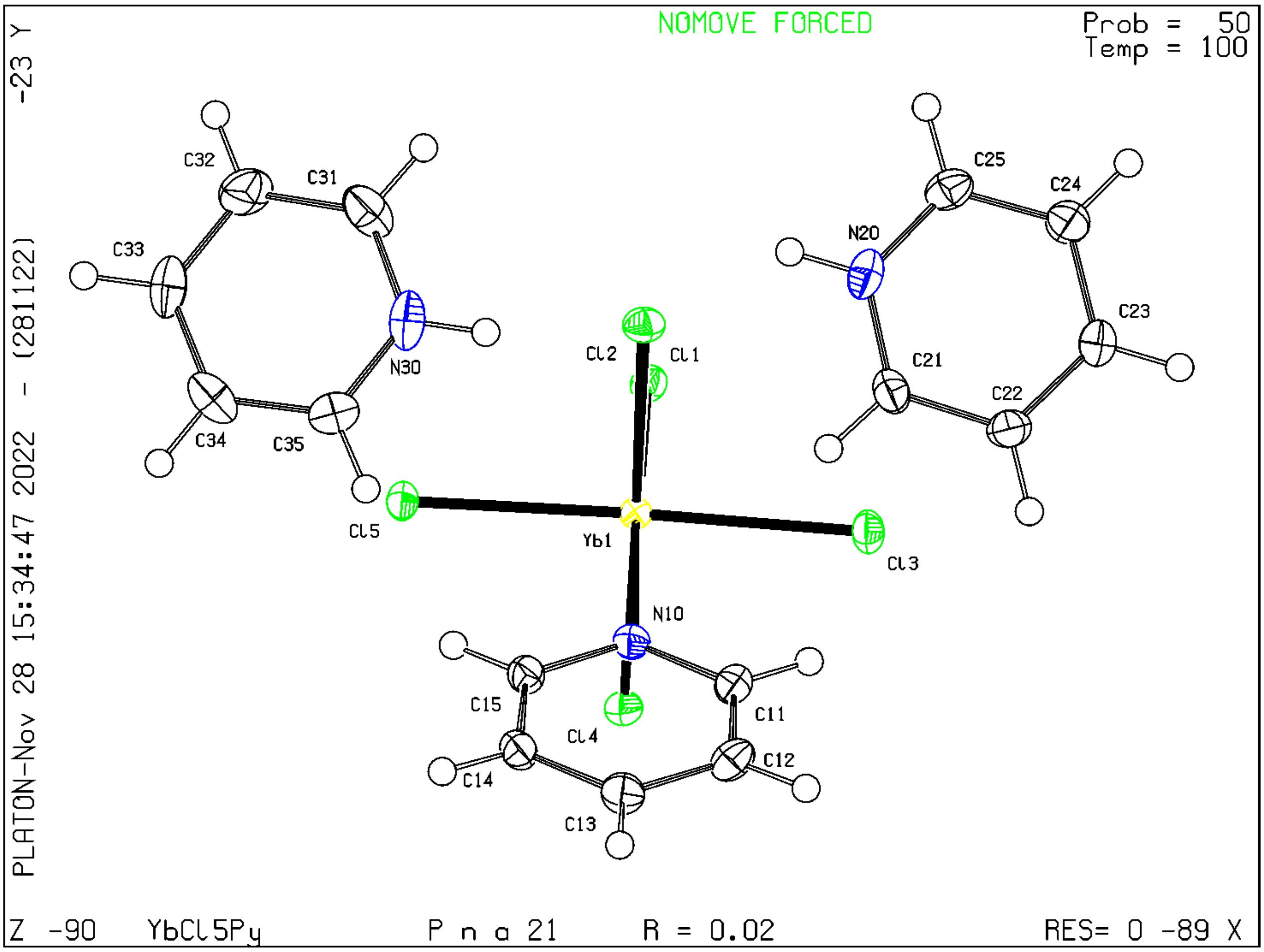

checkCIF (basic structural check) running

Checking for embedded fcf data in CIF ...
Found embedded fcf data in CIF. Extracting fcf data from uploaded CIF, please wait . . . . . .

checkCIF/PLATON (basic structural check)

Structure factors have been supplied for datablock(s) lucl5_d_sx

THIS REPORT IS FOR GUIDANCE ONLY. IF USED AS PART OF A REVIEW PROCEDURE FOR PUBLICATION, IT SHOULD NOT REPLACE THE EXPERTISE OF AN EXPERIENCED CRYSTALLOGRAPHIC REFEREE.

No syntax errors found. CIF dictionary

Please wait while processing .... Interpreting this report

Datablock: lucl5_d_sx

Bond precision:	C-C = 0.0067 A		Wavelength=0.71073
Cell:	a=18.5935(3)	b=7.2703(1)	c=14.7377(3)
	alpha=90	beta=90	gamma=90
Temperature:	100 K		

	Calculated	Reported
Volume	1992.25(6)	1992.25(6)
Space group	P n a 21	P n a 21
Hall group	P 2c -2n	P 2c -2n
Moiety formula	C5 H5 Cl5 Lu N, 2(C5 H6 N)	?
Sum formula	C15 H17 Cl5 Lu N3	C15 H17 Cl5 Lu N3
Mr	591.54	591.54
Dx,g cm-3	1.972	1.972
Z	4	4
Mu (mm-1)	5.628	5.628
F000	1136.0	1136.0
F000'	1137.40	
h,k,lmax	29,11,23	27,10,23
Nref	7987[4123]	7093
Tmin,Tmax	0.346,0.545	0.682,0.746
Tmin'	0.308	

Correction method= # Reported T Limits: Tmin=0.682 Tmax=0.746 AbsCorr = MULTI-SCAN

Data completeness= 1.72/0.89 Theta(max)= 33.765

R(reflections)= 0.0190(6366) wR2(reflections)= 0.0356(7093)

S = 1.045 Npar= 226

The following ALERTS were generated. Each ALERT has the format

test-name_ALERT_alert-type_alert-level.

Click on the hyperlinks for more details of the test.

Alert level C

ABSTY02_ALERT_1_C An _exptl_absorpt_correction_type has been given without a literature citation. This should be contained in the _exptl_absorpt_process_details field.
Absorption correction given as multi-scan

STRVA01_ALERT_4_C Flack test results are ambiguous.
From the CIF: _refine_ls_abs_structure_Flack 0.488
From the CIF: _refine_ls_abs_structure_Flack_su 0.006
PLAT911_ALERT_3_C Missing FCF Refl Between Thmin & STh/L= 0.600 3 Report
PLAT924_ALERT_1_C The Reported and Calculated Rho(min) Differ by . 1.22 eA-3
PLAT974_ALERT_2_C Check Calcd Negative Resid. Density on Lu1 -1.37 eA-3

Alert level G

PLAT111_ALERT_2_G ADDSYM Detects New (Pseudo) Centre of Symmetry . 100 %Fit
PLAT113_ALERT_2_G ADDSYM Suggests Possible Pseudo/New Space Group Pnma Check
Check Model Parameter Symmmetry for Reflection Data Support
PLAT794_ALERT_5_G Tentative Bond Valency for Lu1 (III) . 3.17 Info
PLAT883_ALERT_1_G No Info/Value for _atom_sites_solution_primary . Please Do !
PLAT912_ALERT_4_G Missing # of FCF Reflections Above STh/L= 0.600 208 Note
PLAT913_ALERT_3_G Missing # of Very Strong Reflections in FCF .... 1 Note
PLAT950_ALERT_5_G Calculated (ThMax) and CIF-Reported Hmax Differ 2 Units
PLAT955_ALERT_1_G Reported (CIF) and Actual (FCF) Lmax Differ by . 1 Units
PLAT956_ALERT_1_G Calculated (ThMax) and Actual (FCF) Hmax Differ 2 Units
PLAT978_ALERT_2_G Number C-C Bonds with Positive Residual Density. 3 Info

0 **ALERT level A** = Most likely a serious problem - resolve or explain
0 **ALERT level B** = A potentially serious problem, consider carefully
5 **ALERT level C** = Check. Ensure it is not caused by an omission or oversight
10 **ALERT level G** = General information/check it is not something unexpected

5 ALERT type 1 CIF construction/syntax error, inconsistent or missing data
4 ALERT type 2 Indicator that the structure model may be wrong or deficient
2 ALERT type 3 Indicator that the structure quality may be low
2 ALERT type 4 Improvement, methodology, query or suggestion
2 ALERT type 5 Informative message, check

It is advisable to attempt to resolve as many as possible of the alerts in all categories. Often the minor alerts point to easily fixed oversights, errors and omissions in your CIF or refinement strategy, so attention to these fine details can be worthwhile. In order to resolve some of the more serious problems it may be necessary to carry out additional measurements or structure refinements. However, the purpose of your study may justify the reported deviations and the more serious of these should normally be commented upon in the discussion or experimental section of a paper or in the "special_details" fields of the CIF. checkCIF was carefully designed to identify outliers and unusual parameters, but every test has its limitations and alerts that are not important in a particular case may appear. Conversely, the absence of alerts does not guarantee there are no aspects of the results needing attention. It is up to the individual to critically assess their own results and, if necessary, seek expert advice.

Publication of your CIF in IUCr journals

A basic structural check has been run on your CIF. These basic checks will be run on all CIFs submitted for publication in IUCr journals (*Acta Crystallographica*, *Journal of Applied Crystallography*, *Journal of Synchrotron*

Radiation); however, if you intend to submit to *Acta Crystallographica Section C* or *E* or *IUCrData*, you should make sure that full publication checks are run on the final version of your CIF prior to submission.

Publication of your CIF in other journals

Please refer to the *Notes for Authors* of the relevant journal for any special instructions relating to CIF submission.

PLATON version of 28/11/2022; check.def file version of 28/11/2022

Datablock lucl5_d_sx - ellipsoid plot

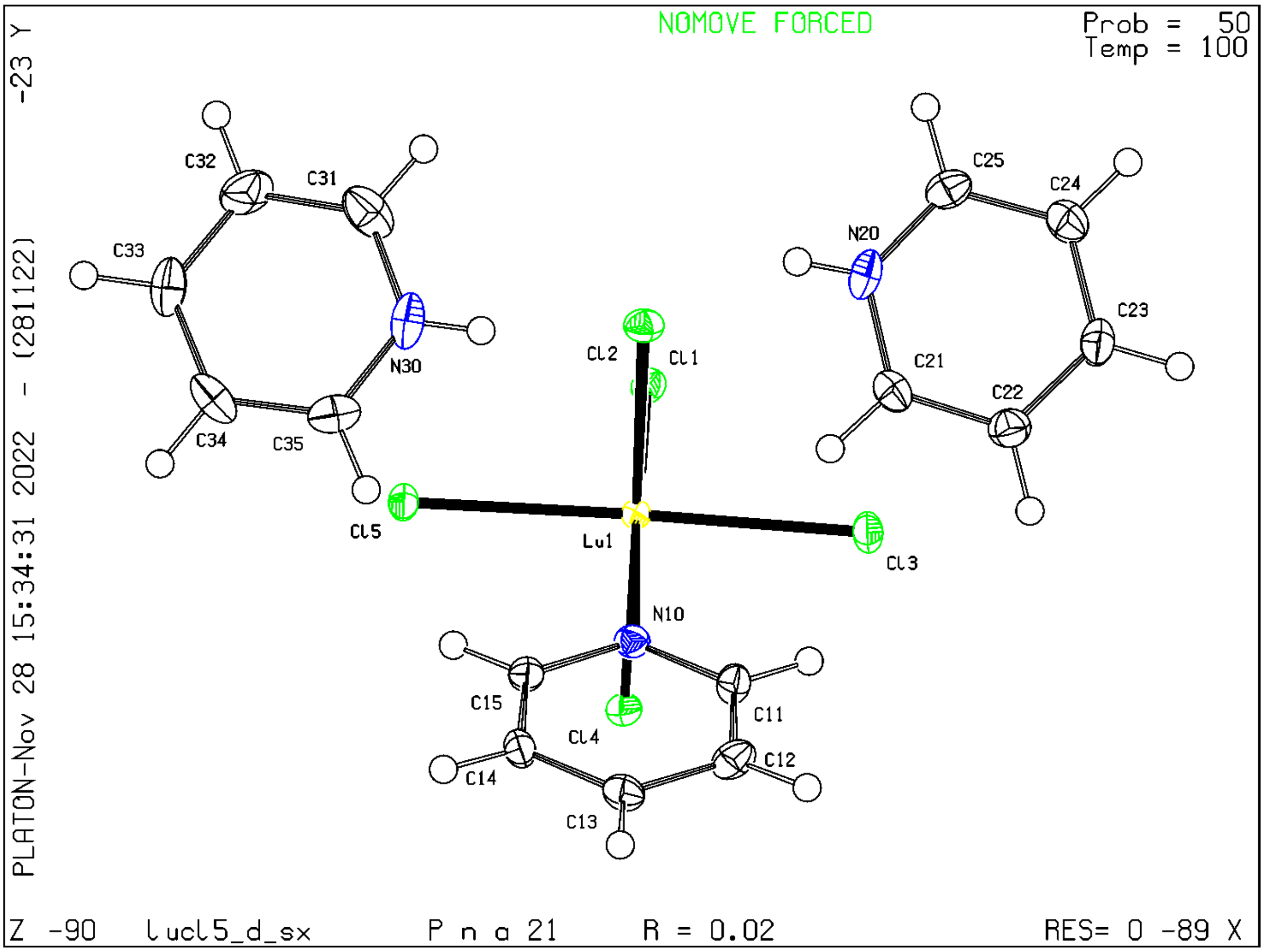

Supplement: Supplementary file 1 [file molecules-28-00283-s001.zip › molecules-2045170-supplementary.pdf]
